# Deciphering the Self-Catalytic Mechanisms of Polymerization and Transesterification in Polybenzoxazine Vitrimers

**DOI:** 10.1021/jacs.4c02153

**Published:** 2024-05-02

**Authors:** Antoine Adjaoud, Benoit Marcolini, Reiner Dieden, Laura Puchot, Pierre Verge

**Affiliations:** † Luxembourg Institute of Science and Technology, 5 Avenue des Hauts-Fourneaux, Esch-sur-Alzette L-4362, Luxembourg; ‡ University of Luxembourg, 2 Avenue de Université, Esch-sur-Alzette L-4365, Luxembourg

## Abstract

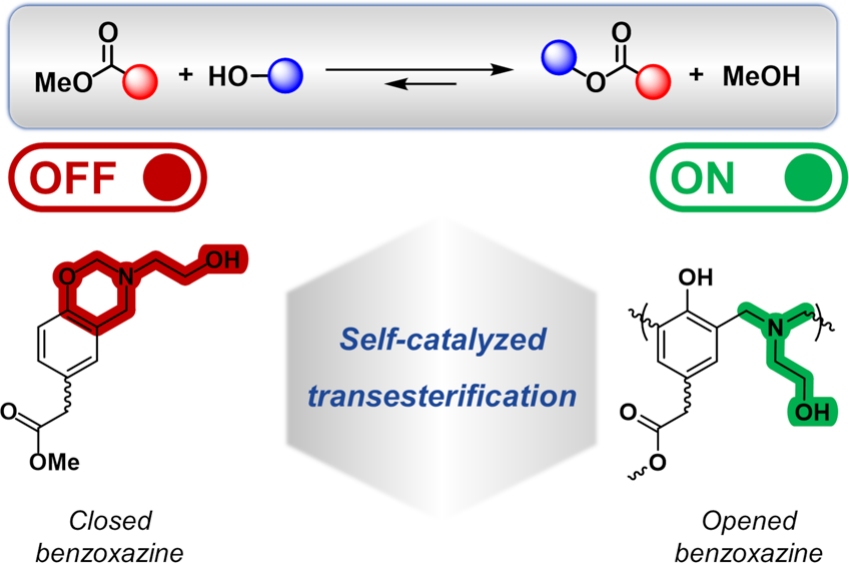

The use of internal catalysts has emerged as a pivotal design principle to facilitate dynamic exchanges within covalent adaptable networks (CANs). Polybenzoxazines, specifically, have shown considerable potential in generating vitrimers through thermally induced transesterification reactions catalyzed internally by tertiary amines. This study aims to investigate the chemical complexities of transesterification reactions within benzoxazine vitrimers. To achieve this, model molecules using various phenolic acids and amino-alcohol derivatives were synthesized as precursors. The structure of these model molecules was fully elucidated by using nuclear magnetic resonance (NMR). Differential scanning calorimetry (DSC) and rheology experiments evidenced the accelerated network formation of the precursors due to the presence of aliphatic −OH groups. Thermogravimetric analysis coupled with microcomputed gas chromatography (TGA-μGC) was used to provide evidence of transesterification reactions. The results showed that the spatial proximity between tertiary amine and hydroxyl groups significantly enhances the rate exchange, attributed to a neighboring group participation (NGP) effect. Interestingly, kinetic experiments using complementary NMR techniques revealed the thermal latency of the tertiary amine of benzoxazine toward transesterification reactions as its opening is needed to trigger the dynamic exchange. The study highlights the crucial role of steric hindrance and tertiary amine basicity in promoting the dynamic exchange in an internally catalyzed system.

## Introduction

The issue related to the limited end-of-life options for thermosets has given rise to new research thematically in the field of polymers. Among the existing strategies, the incorporation of dynamic covalent bonds in cross-linked networks offers an alternative end-of-life scenario for thermosetting resins.^1^ Vitrimers, a subclass of covalent adaptable networks (CANs), can change their topology without losing their network integrity.^2^ Pioneered by Leibler and colleagues,^3−5^ research works dedicated to the design of vitrimers relying on transesterifications reactions (TERs) focus the majority of attention.^6^ Transesterification is a thermoactivated process that involves the alcoholysis of ester bonds following an associative path. The intermolecular addition is followed by the expulsion of the alkoxide, leading to the formation of functional groups with either the initial or swapped substituents. Transesterification typically requires the use of a highly active catalyst, such as a strong Lewis acid or base, transition metal complex, or organic salt, to trigger dynamic exchange on a reasonable time and temperature scale. The concentration and type of external catalyst can significantly influence the kinetics of the bond exchange, with higher catalyst loadings resulting in fastest exchanges.^4,5,7^ However, the use of external catalysts is suspected to lead to phase separation, leaching, deactivation, or side reactions^8^ and could result in the premature degradation of the polymer matrix, constituting a potential obstacle to industrial applications.^2^

One of the most promising approaches to address these issues is the embedding of the catalyst within a cross-linked network to reduce the activation energy required for the dynamic exchange to occur,^9,10^ leading to so-called self-catalytic CANs. Excess amounts of aliphatic^11,12^ or carboxylic^13^ groups can accelerate the kinetics of transesterification reactions through hydrogen bonding. The catalytic effect can also be enhanced by proximity-induced activation, known as neighboring group participation (NGP),^9,10^ which enhances the rate of transesterification. The electron-withdrawing effect,^14,15^ intramolecular cyclization process,^16,17^ and proton transfer exchange^18,19^ are examples of NGP-assisted transesterification. Covalently bonded tertiary amine groups (NR_3_) acting as base catalysts for dynamic exchange have recently been introduced to develop self-catalytic vitrimers.^20,21^ NR_3_ groups can be incorporated within a network by using amino glycidyl precursors^21−24^ and primary^20,24−27^ or tertiary amine-containing^28,29^ curing agents or also through aza-Michael addition.^15,30,31^ The position of the catalytic group within the polymer backbone has a significant effect on the kinetics of exchange. Hayashi and Inaba suggested that attaching NR_3_ groups on the side groups of the network strands^29^ rather than at the cross-link points^22^ can significantly enhance the exchange rate of transesterification. Du Prez et al. demonstrated that positioning NR_3_ groups in the β-position relative to the alcohol groups accelerates the kinetic of transesterification.^18^ In another study, Bories-Azeau and Armes et al. showed that the basicity of the immobilized NR_3_ catalyst governs the relative propensity of NR_3_-containing methacrylates to undergo transesterification.^32^ A higher rate was observed for the less hindered NR_3_ groups, suggesting that their catalytic efficiency is influenced by their chemical environment.

The unique structural features of polybenzoxazines (PBZs), which include in situ-generated NR_3_ embedded in a cross-linked network, make them promising candidates for internally catalyzed vitrimers.^33,34^ PBZs are obtained from the thermally accelerated ring-opening polymerization (ROP) of benzoxazine monomers and undergo curing through an autocatalytic cationic mechanism without the need for any hardener or external catalyst.^35^ The concept of NGP also extends to the chemistry of benzoxazines and their autocatalytic polymerization.^36^ In 2010, Endo et al. introduced the concept of the “acceleration of the polymerization of benzoxazine by neighboring group participation of nucleophilic moiety”.^37^ The higher reactivity and faster polymerization of *N*-(2-hydroxyethyl)-containing benzoxazine originate from the NGP of the hydroxyl group. In a similar fashion, Lochab and colleagues elegantly demonstrated the formation of the thiazolidine ring in *N*-(2-thiolyethyl)-containing benzoxazine, facilitating the benzoxazine ring-opening reaction at lower temperatures.^38^ The versatile design of benzoxazine resins allows for the integration of dynamic functionalities, such as ester bonds, within their network. Our group has demonstrated that thermally induced transesterification reactions can endow PBZs with a macroscopic flow and recyclability aptitudes but also contribute to accelerating their polymerization.^39−41^ However, to the best of our knowledge, the chemical occurrence of transesterification in PBZ vitrimers and the role of NR_3_ in the activation of the dynamic exchange have never been explicitly investigated and are underexploited.

To better understand the polymerization and kinetics of bond exchange, a set of model compounds were synthesized. By adjusting the functionality and reactivity of each molecule, we were able to determine the mechanisms of ring opening polymerization and transesterification within PBZ vitrimers. These model molecules were created by reacting various phenolic acids (phloretic acid, diphenolic acid, and 4-hydroxybenzoic acid) with methanol through Fischer esterification to form monoester derivatives. These derivatives were then reacted with various amino-alcohol derivatives (monoethanolamine, diethylene glycol amine, and triethylene glycol amine) in a Mannich-like condensation reaction. The structure of the model compounds was confirmed by nuclear magnetic resonance (NMR) analysis, and the kinetics of benzoxazine ROP was followed by differential scanning calorimetry (DSC) analysis and rheology. Transesterification was confirmed through thermogravimetric analyses coupled with microcomputed gas chromatography (TGA-μGC) on the model compounds. During these tests, the product of transesterification, methanol, was identified well and quantified on a GC chromatogram. ^1^H and ^1^H–^15^N heteronuclear multiple bond correlation (HMBC) NMR analyses were also employed to gain a deeper understanding of the NGP of the NR_3_ generated from benzoxazine ROP. These analyses unveil the thermal latency of the dynamic exchanges that necessitate the initiation of the benzoxazine ring opening process.

## Results and Discussion

### Synthesis and Structural Characterization of Benzoxazine Vitrimer Model Molecules

**Figure 1:**
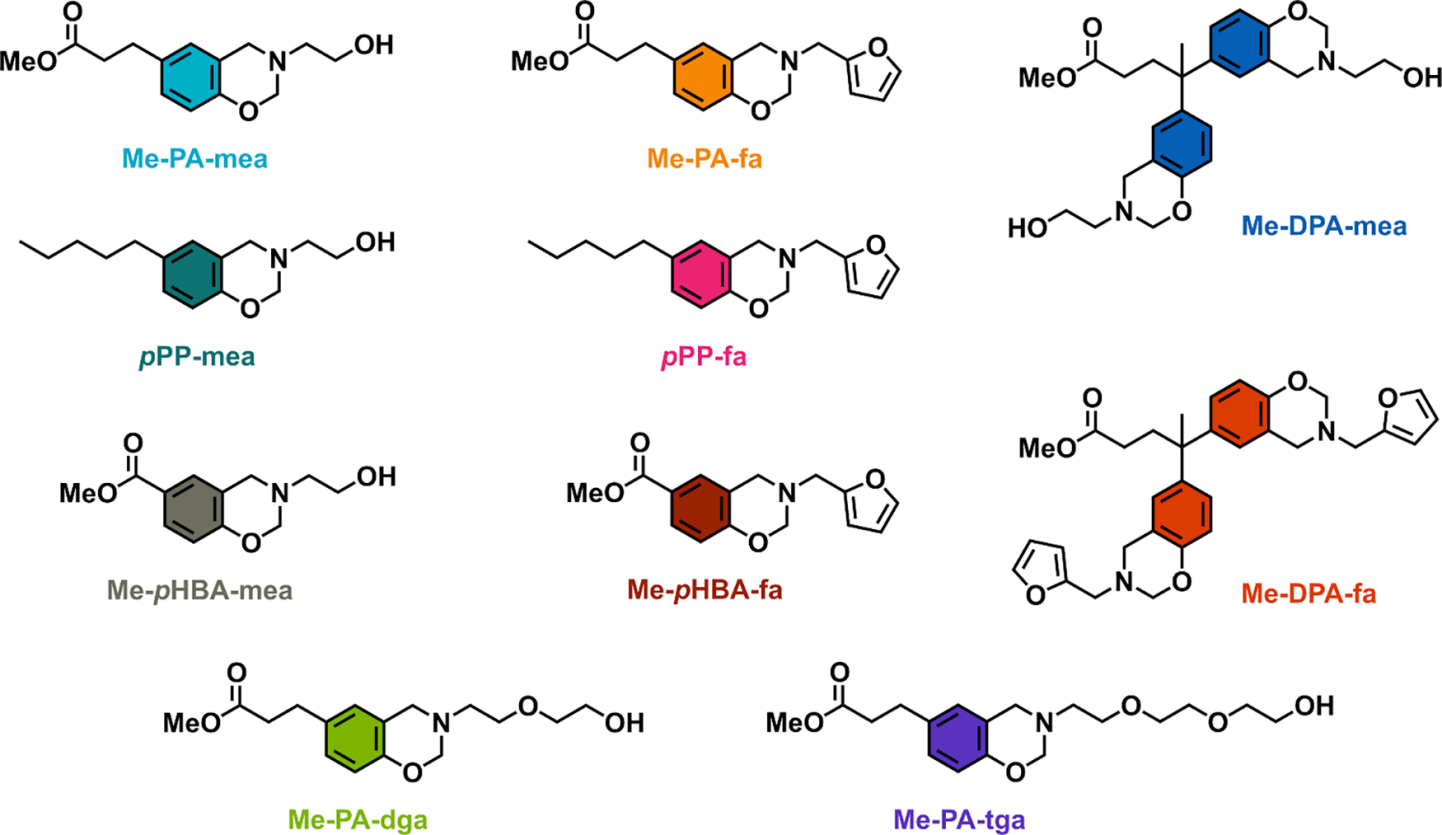
Structure of benzoxazine vitrimer model molecules.

Several studies have reported the use of various polybenzoxazine-based vitrimers that utilize TERs.^39−41^ These materials exhibit a combination of high thermal and mechanical properties, as well as unique dynamic behavior. The configuration of the dynamic networks in all these systems is similar, with the aliphatic −OH situated at the β-position of the NR_3_ in the benzoxazine ring. In these systems, the involvement of the phenoxy −OH groups in TERs and the mechanism of NGP are still unclear, and there is a lack of experimental evidence to understand their role. Additionally, PBZ vitrimers relying on TERs have been found to have a low curing temperature compared to conventional benzoxazines, and the origin of this accelerating effect also needs to be clarified. To simulate the chemical reactions that take place in these systems, a range of model molecules have been synthesized to differentiate and assess the role of each substituent. Their structures and their key attributes are reported in Figure 1and Table 1, respectively.

**Table 1:** Structural Features of Benzoxazine Vitrimer Model Molecules^a^

model molecule	aliphatic ester	aromatic ester	aliphatic −OH^a^
Me-PA-mea/Me-DPA-mea	X		X^a^
Me-PA-dga	X		X
Me-PA-tga	X		X
Me-PA-fa/Me-DPA-fa	X		
Me-*p*HBA-mea		X	X^a^
Me-*p*HBA-fa		X	
*p*PP-PA-mea			X^a^
*p*PP-PA-fa			

a Aliphatic −OH in the β-position relative to NR_3_.

The molecules were synthesized following the two-step process reported in previous works with minor modifications (Scheme S1). Monofunctional phloretic acid, diphenolic acid, and *para*-hydroxy benzoic acid derivatives (PA, DPA, and *p*HBA, respectively) were reacted with methanol through Fischer esterification to obtain phenolic monoester derivatives in high purity (Figures S1–S3). The reaction was carried out for 12 h in the presence of an acid catalyst (*para*-toluene sulfonic acid) at the reflux temperature of methanol. The ring-closure process of benzoxazine rings was performed in a second step through solventless Mannich-like condensation with a stoichiometric amount of paraformaldehyde and amine precursors.

**Figure 2:**
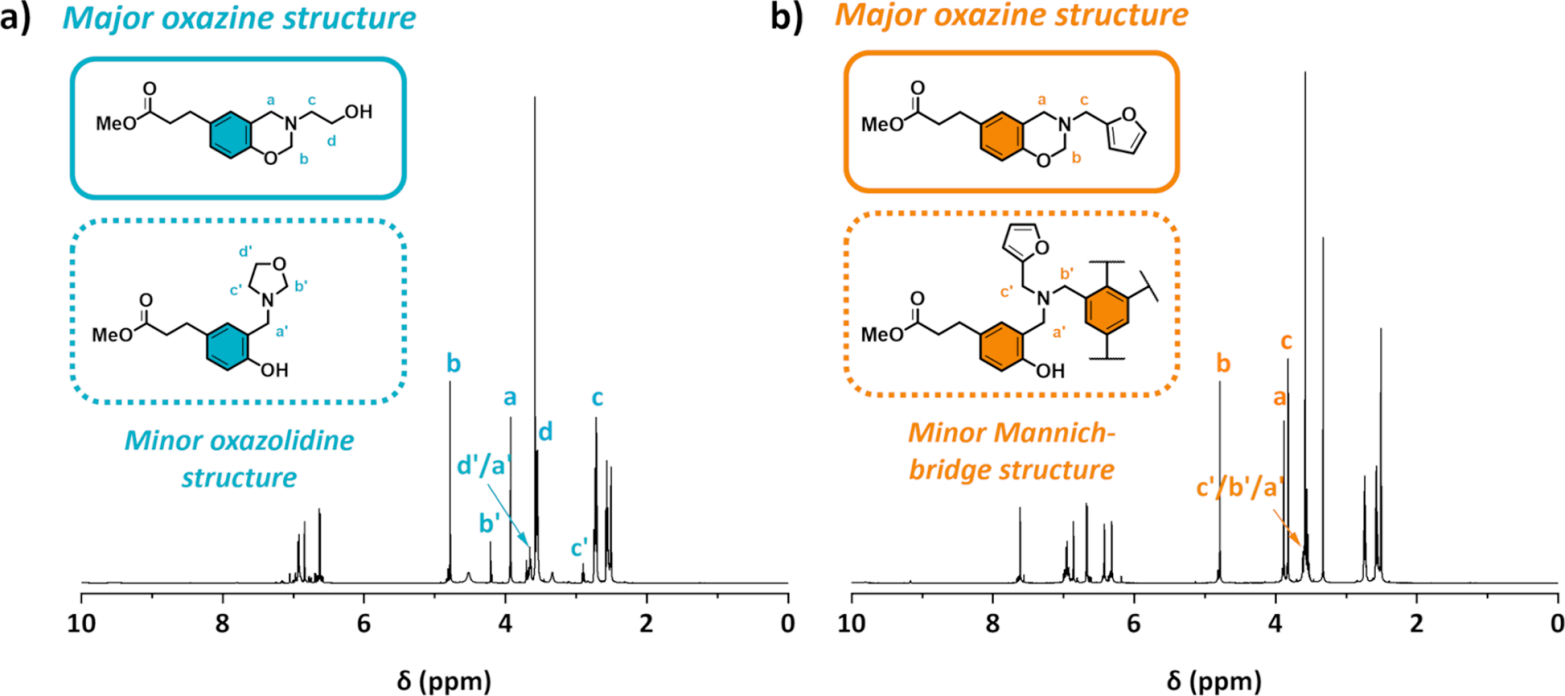
^1^H NMR spectrum of (a) Me-PA-mea and (b) Me-PA-fa with identification of major and minor structures.

Three amino-alcohol derivatives were used: monoethanolamine (mea), diethylene glycol amine (dga), and triethylene glycol amine (tga). Me-PA-mea is a precursor containing both an aliphatic ester and −OH group in the β-position of the tertiary amine. Me-PA-dga and Me-PA-tga have both aliphatic esters and −OH groups, but these are not in the β-position of the tertiary amine. The ester of the precursor Me-*p*HBA-mea is aromatic. Next to this, model molecules lacking the aliphatic −OH group were synthesized from furfurylamine (fa). Additional model molecules were prepared from the Mannich-like condensation of 4-pentylphenol (*p*PP) in similar conditions. *p*PP-PA-mea and *p*PP-PA-fa molecules have been meticulously developed as ester-free reference standards. They feature a phenolic *para*-substitution of a similar length to that of monoester analog (i.e., Me-PA-mea and Me-PA-fa). Me-DPA-mea and Me-DPA-fa are difunctional precursors (with two benzoxazine moieties per ester bonds). They were used as model molecules for kinetic ^1^H and ^1^H–^15^N NMR analyses reported later in this article to confirm the NGP-assisted transesterification mechanisms. The structure of the model molecules was confirmed by ^1^H and ^13^C NMR (Figures S4–S13). The formation of benzoxazine cyclic ring structures was confirmed by the disappearance of the phenolic peak and the appearance of two signals at δ ≈ 3.9 and δ ≈ 4.8 ppm corresponding to the methylene bridges in the oxazine ring (Ar–CH_2_*–N and N–CH_2_*–O, respectively). The integration of these peaks reaches nearly 75% (Table S2) and is indicative of the formation of substructures, which depends on the type of amine as shown in Figure 2.

According to the literature, β-amino-alcohol adduct can lead to the formation of oxazolidine motifs when they are used in a Manich-like reaction with phenol.^37^ The formation of such structures can be explained by the formation of a zwitterionic intermediate during the synthesis. The presence of oxazolidine was identified for molecules synthesized with monoethanolamine (i.e., Me-PA-mea, Me-DPA-mea, Me-*p*HBA-mea, and *p*PP-mea). Figure 2a depicts the ^1^H NMR of Me-PA-mea, where the peaks characteristic of oxazolidine protons are located at δ = 2.90, 3.70, and 4.21 ppm. No traces of oxazolidine were detected for model molecules synthesized from the molecules prepared with longer amino alcohol (i.e., Me-PA-dga and Me-PA-tga). For aminofuran derivative precursors (i.e., Me-PA-fa, Me-DPA-fa, Me-*p*HBA-fa, and *p*PP-fa), a minor structure is revealed, corresponding to ring-opened oxazine (Figure 2b). The characteristic cross-peaks in the HSQC NMR spectrum of minor oxazolidine-like and Mannich-bridged structures are marked in Figures S14 and S15, respectively.

### Monitoring of Benzoxazine Ring-Opening Polymerization

While the curing of benzoxazine monomers is known to require high temperatures to initiate the ring-opening reaction (typically around 200 °C), we have previously reported that the ROP of benzoxazine vitrimers can occur at temperatures as low as 120 °C under catalyst-free conditions.^39−41^ Alcohol groups are known to facilitate the ring-opening reaction through protonation of the oxazine ring initiating subsequent polymerization reactions.^42^ Endo et al. suggested that the alkoxide species in β-amino-alcohol is a strong nucleophile as the ionic intermediate is stabilized through intramolecular cyclization reaction.^37^

The DSC thermograms of the model molecules are displayed in Figures S16–S19. The first exothermic peak detected in the range of 115–200 °C corresponds to the ring opening of the benzoxazine groups. A second exothermic peak is detected in the range of 225–250 °C and is associated with the thermal degradation of the benzoxazine precursor. The onset temperature (*T*_onset_) of each precursor is reported in Table S3. Model molecules with aliphatic −OH in the β-position of NR_3_ display the lower onset of thermal polymerization (*T*_onset_ ≈ 115–135 °C). The acceleration of the ring-opening reaction is less pronounced when NR_3_ and −OH groups are further distant (*T*_onset_ = 146 and 183 °C for Me-PA-dga and Me-PA-tga, respectively). This phenomenon could not be explained solely by the lower concentration of the aliphatic −OH groups. Indeed, previous reports indicate that alcohol-containing benzoxazine monomers with a higher concentration of −OH groups do not exhibit such rapid polymerization behavior.^43,44^ When β-amino alcohol moieties are present, the accelerated ring-opening reaction of the benzoxazine monomer originates from the neighboring group participation of aliphatic −OH. Indeed, the phenolic or aliphatic −OH groups from oxazolidine and oxazine structures, respectively, can activate polymerization at lower temperatures (Scheme S1). However, in the absence of aliphatic −OH groups, the ring-opening reaction of benzoxazine requires higher temperatures, more in the range of conventional benzoxazines (*T*_onset_ > 160 °C).

**Figure 3:**
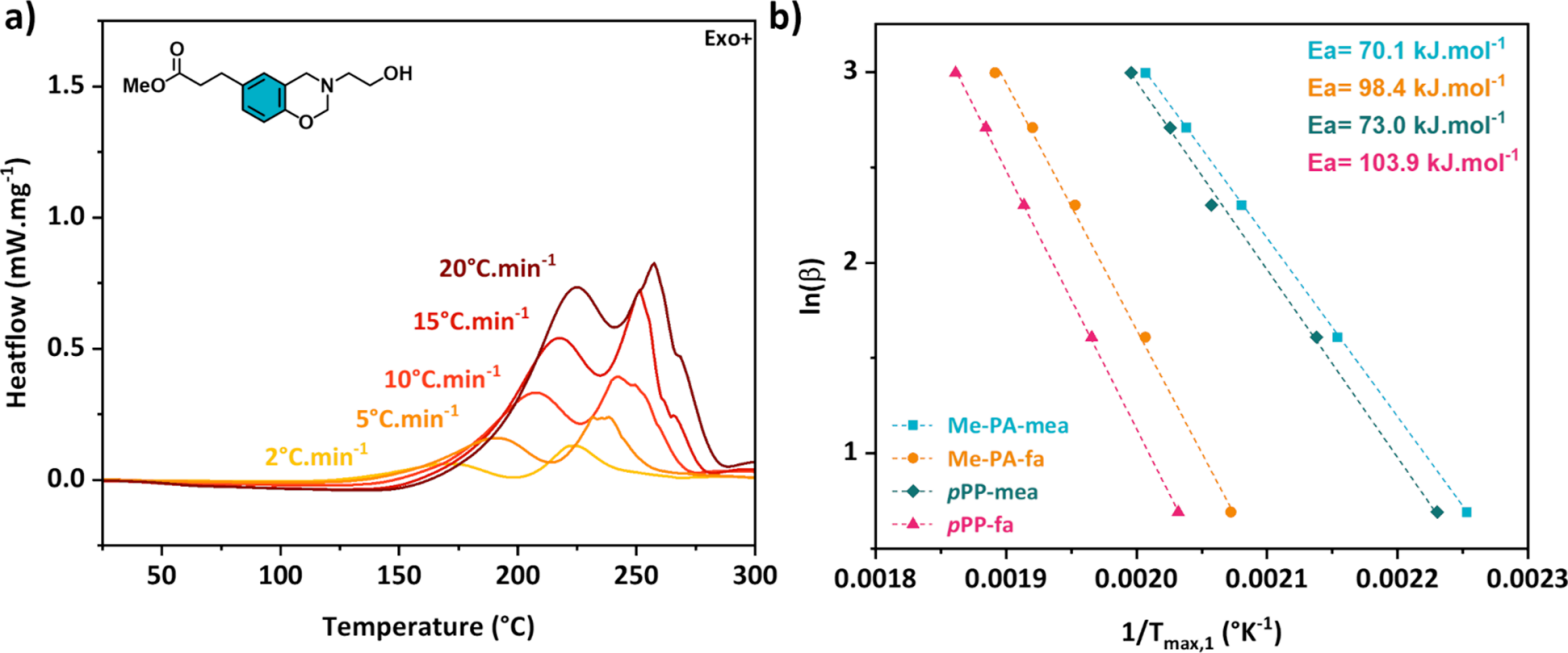
(a) DSC thermograms of Me-PA-mea with the heating rate ranging from 2 to 20 °C·min^–1^ and (b) determination of the activation energy of benzoxazine ring-opening polymerization using the FWO equation: ln(β) against 1/*T*_p_.

Figure 3a compiles the DSC thermograms of Me-PA-mea performed at different heating rates (from 2 to 20 °C min^–1^). The DSC thermograms for other molecules are displayed from Figures S20–S26. The linear fitting extrapolated from Kissinger^45^ and Flynn–Wall–Ozawa^46,47^ (FWO) equations (using the peak temperature, *T*_p_) is reported in Figure 3b and Figure S27, respectively. The average activation energies of benzoxazine ROP (*E*_aROP_) were found to be 82.0 and 85.1 kJ·mol^–1^ for Me-PA-mea and *p*PP-mea, respectively. The higher *E*_aROP_ determined for Me-*p*HBA-mea and Me-*p*HBA-fa (*E*_aROP_= 93.6 and 98.9 kJ·mol^–1^, respectively) can be explained by the mesomeric effect of the aromatic ester stabilizing the oxazine ring. Finally, the activation energies for the model molecules without aliphatic −OH groups are 112.3 and 118.3 kJ·mol^–1^ for Me-PA-fa and *p*PP-fa, respectively. In summary, molecules containing aliphatic −OH require lower *E*_a_ as they catalyze the polymerization mechanism. The acceleration of the benzoxazine ring-opening reaction is still more pronounced for aliphatic −OH involved in an NGP effect (i.e., Me-PA-mea) as evidenced by the lower *E*_aROP_ (Table S3, lines 1–3). Additionally, by comparing the *E*_aROP_ of Me-PA-mea and *p*PP-mea together (or Me-PA-fa and *p*PP-fa), it is clear that ester bonds do not affect the kinetics of polymerization. Numerical optimization of the isoconversional Friedman method was also performed on the deconvoluted exothermic peaks.^48^ The conversion rates as a function of the temperature are reported from Figures S28–S35. The apparent activation energy of benzoxazine ROP (*E*_αROP_), a function of the conversion rate, follows a similar trend as the *E*_aROP_ determined from the Kissinger and FWO equations (Table S3, column 5).

### Monitoring of Transesterification Reactions

The thermally induced transesterification reaction is a process that generates a new ester and new alcohol. The dynamicity of the bond exchanges in CANs is generally characterized by thermomechanical analysis, specifically stress-relaxation experiments. This method provides insight solely into the macroscopic flow and molecular mobility of the polymer chains.^9^

**Scheme 1:**
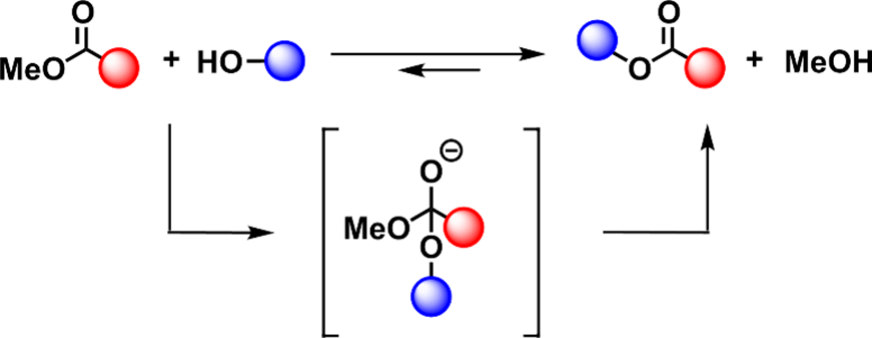
Transesterification Reactions between an Alcohol and a Methyl Ester

The direct probe of organic reactivity has been highlighted by ^1^H NMR spectroscopy^16,18^ or GC coupled with a mass spectroscopy (MS) detector.^3−5^ An attempt to prove the occurrence of transesterification between the phenolic groups of a bisphenol F-based polybenzoxazine matrix and dimethyl phthalate has been previously investigated by thermogravimetric analysis (TGA).^49^ The gradual decrease in weight was attributed to the release of methanol as an expected product of transesterification externally catalyzed by zinc acetate. In this work, the model molecules were designed to release methanol during TER as shown in Scheme 1.

The formation of methanol can be proved and quantified as the temperature range of transesterification reactions (*T* > 100 °C) is higher than its boiling point (64.7 °C). In the conditions where the exchanges take place, the evaporation of methanol shifts the degenerative equilibrium toward a quantitative reaction. In-depth characterization of the mass-loss event could explicitly confirm the formation of methanol as transesterification adduct.

#### Tracking Methanol Release in Transesterification through Evolved Gas Analysis

Thermogravimetric analysis coupled with a microgas chromatography detector (TGA-μGC^50^) stands for a powerful hyphenated technique for the qualitative and quantitative identification of methanol released from the TER of the model molecules.

**Figure 4:**
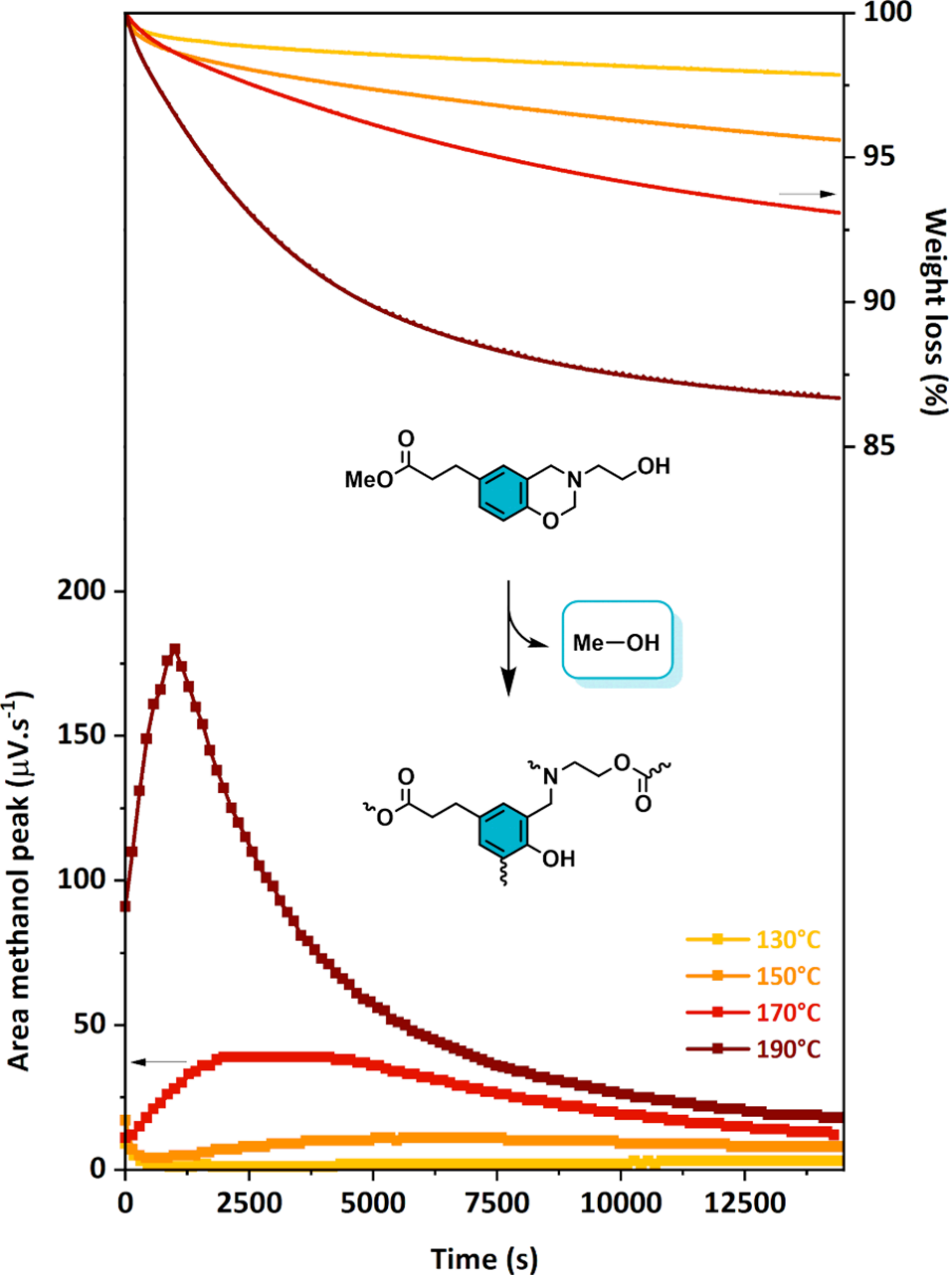
Isothermal TGA curves of Me-PA-mea (top of the figure) and corresponding area of the methanol peak determined on the μGC spectrum (bottom of the figure).

Figure 4 depicts the mass loss of Me-PA-mea (TGA) and the amount of methanol release (μGC) from 130 to 190 °C as a function of time. Within this temperature range, the numerous intramolecular hydrogen bonds provided by the −OH groups in β-hydroxyethyl-terminated monofunctional benzoxazine serve to stabilize Mannich bridges and prevent thermodegradation. Below 130 °C, the release of methanol is not detected by μGC. The weight loss observed in the TGA experiment at 150 °C could be assigned only to the evaporation of methanol as an occurrence of transesterification reactions as evidenced by the corresponding μGC curve. The thermal dependence of the dynamic exchange is highlighted by the higher methanol detection with increasing temperature. At 190 °C, an equilibrium is reached at longer times, as the experimental weight loss of 13.3% fits with the theoretical weight loss if all methyl esters were consumed (12.1%). It is worth noting that the rate of release of methanol at the different temperatures may be underestimated, as it needs to diffuse through the monomer after its formation, and the accurate determination of the rate of release would require more investigation. However, these analyses are enough to conclude that methanol is indeed released at temperatures above 150 °C. Further experiments were performed on other model molecules to identify the structural features affecting the rate of transesterification reactions (Figures S36–S43). The involvement of the phenoxy −OH group was ruled out by comparing Me-PA-mea and Me-PA-fa. It was observed that no methanol release occurred during the experiment for the latter compound (Figures S36 and S37). The significant weight loss observed in this alcohol-free monofunctional compound further evidences the crucial role of the wealth of hydrogen bonding in improving the thermal stability of the monomer.^51−53^ The difunctional precursor Me-DPA-mea behaves similarly to its monofunctional counterpart, albeit with a lower release of methanol (Figures S38 and S39). It is attributed to the lower concentration of methyl ester in this molecule. Methanol is hardly detected from analysis done on Me-*p*HBA-mea at 190 °C (Figures S40 and S41), suggesting that the aromatic ester is less reactive toward transesterification than its aliphatic counterpart, presumably due to the mesomeric effect of the aromatic ring stabilizing the tetrahedral intermediate in associative transesterification exchange.

Model molecules synthesized with amino-alcohols with increased distance between the −OH and NR_3_ groups were also studied. Figures S42 and S43 illustrate the importance of the relative position of the tertiary amine in the kinetics of transesterification reactions. Despite having a lower concentration of dynamic functionalities in Me-PA-dga and Me-PA-tga precursors, the exchange reaction still occurred with a methanol release that was three times lower than for Me-PA-mea. This suggests that the length of the amino-alcohol had a significant effect on the rate of the dynamic exchange. This can be attributed to the rate enhancement of transesterification reactions through the participation of the neighboring β-amino-alcohol group in the mea-based precursor. In contrast, dga- or tga-based precursors only benefit solely from the internal catalysis of the tertiary amines. Therefore, the structural design of benzoxazine vitrimer precursors drives the rate exchange of transesterification. To maximize the rate of transesterification reactions, the optimal design of the benzoxazine vitrimer precursor should include aliphatic esters and aliphatic −OH groups, with the latter participating in an NGP with a spatially close tertiary amine.

#### Tracking Methanol Release in Transesterification through Spectroscopic Analysis

**Figure 5:**
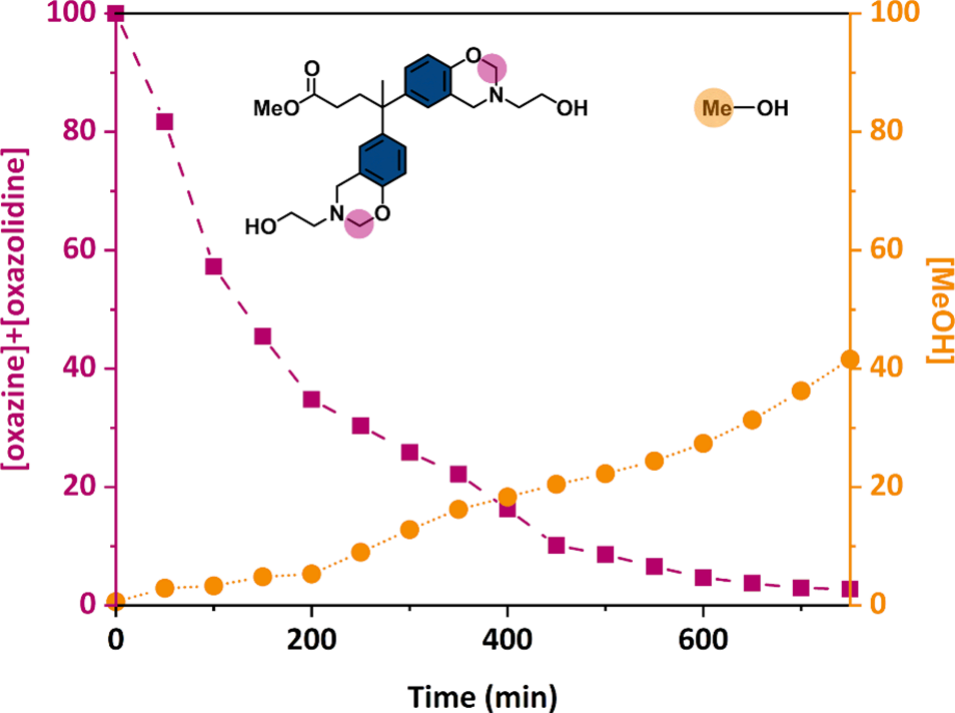
^1^H kinetic NMR experiment: evolution of the concentration of benzoxazine (oxazine and oxazolidine) and methanol at 140 °C (0.6 mL of DMSO-*d*_6_ and 200 mg of Me-DPA-mea).

A study of the kinetic behavior of benzoxazine ring-opening and transesterification reactions was carried out at 140 °C using solution state ^1^H NMR spectroscopy. The initial model molecule Me-PA-mea was not suitable for this study as it does not react below 140 °C, which is the maximum temperature analysis of the spectroscope. Therefore, the fully similar molecule Me-DPA-mea, which has higher functionality, was employed instead of Me-PA-mea to increase the possibility of observing structural alterations. The ^1^H kinetic NMR spectra of Me-DPA-mea can be found in Figure S44. Over time, the methylene peaks associated with oxazolidine and oxazine structures (δ = 4.23 and 4.79 ppm, respectively) gradually decrease, allowing for the appearance of new peaks. When the benzoxazine rings open, a new downfield signal appears and is assigned to the methyl peak of methanol (CH_3_*–OH, δ = 3.26 ppm). This confirms that transesterification reactions occur, resulting in the production of methanol. Figure 5 shows the evolution of the concentration of benzoxazine and methanol signals with time. The opening of benzoxazine rings is almost quantitative as the methylene signals almost steadily disappear as the benzoxazine ROP progresses. Briefly, the experiments show that the benzoxazine ROP and TER occur at the same temperature range, but TER does not seem to play a role in the ROP process. However, the presence of −OH groups enhances ROP, and this effect is more pronounced when the −OH groups are close to NR_3_, leading to an NGP effect.

### Mechanism of Self-Catalyzed Transesterification in Benzoxazine Vitrimers

**Figure 6:**
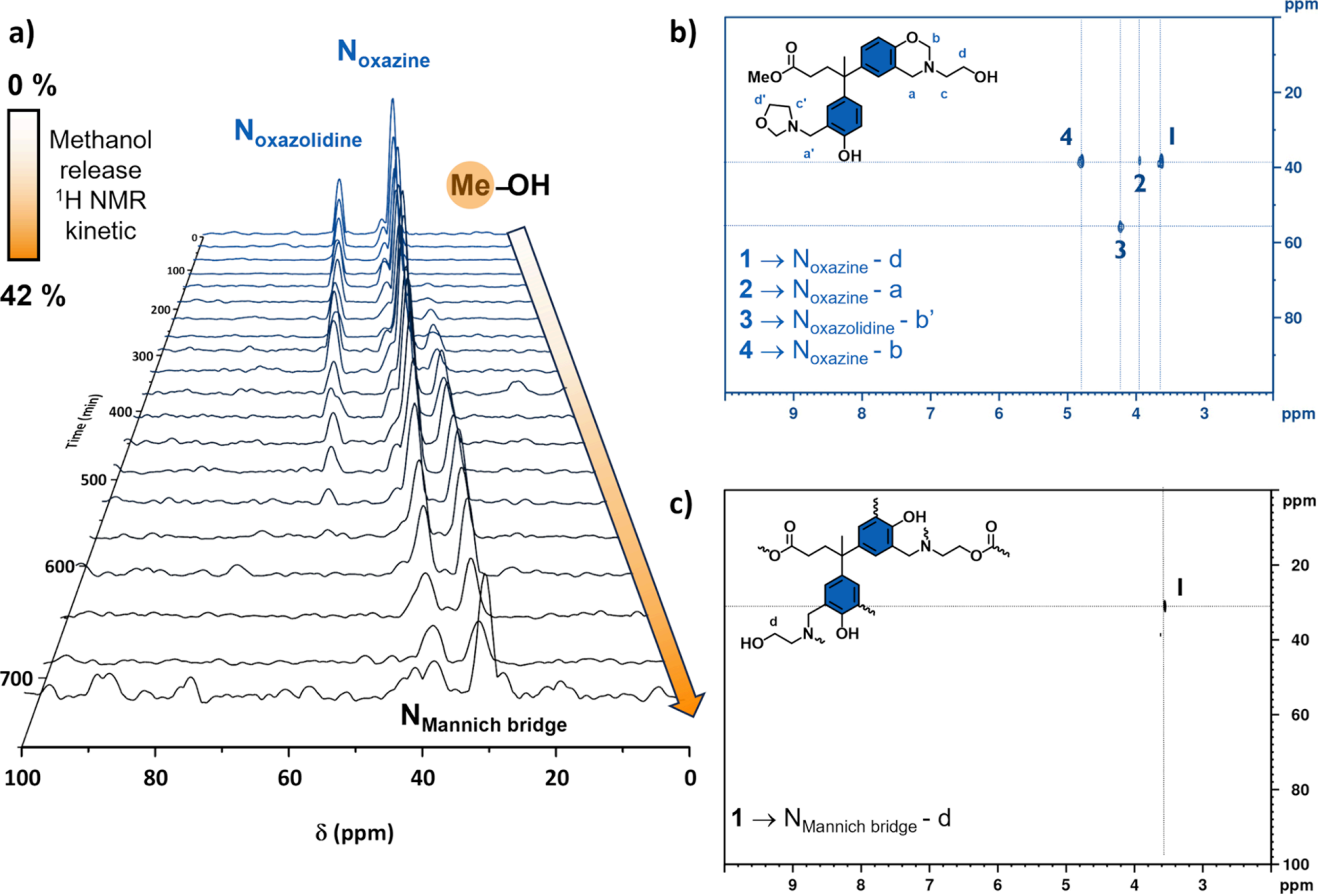
^1^H–^15^N HMBC kinetic NMR of Me-DPA-mea at 140 °C: (a) evolution of individual ^15^N dimension as a function of time (correlated with methanol release determined by ^1^H kinetic NMR experiment). (b) Initial and (c) final ^1^H–^15^N HMBC spectrum.

The ^1^H NMR kinetic analysis provides direct evidence of transesterification reactions and indicates that the ring-opening reaction of benzoxazine precedes these reactions. Covalently bonded NR_3_ may promote dynamic exchange through an internal catalysis effect. The occurrence of thermally induced transesterification reactions without the addition of an external catalyst suggests that the NR_3_ generated in situ during benzoxazine ROP may catalyze the exchange. To support this hypothesis, ^1^H–^15^N heteronuclear multiple bond correlation kinetic analysis was performed to understand the role of NR_3_ on transesterification reactions. The evolution of the ^15^N projection as shown in Figure 6 is depicted as a function of time. As the ring-opening reaction of benzoxazine progresses, the nitrogen signals originating from oxazine and oxazolidine structures gradually disappear (δ = 38.1 and 55.6 ppm, respectively), and a single peak emerges at a lower chemical shift (δ = 30.8 ppm). This new peak corresponds to the Mannich bridge in polybenzoxazine, and its shift to a lower chemical shift indicates that NR_3_ becomes less hindered and more basic. Notably, the emergence of this new form of NR_3_ coincides with the detection of methanol by ^1^H NMR (Figure 5). The results suggest that the release of MeOH, and thus TER, is more likely to occur when the benzoxazine ring is opened, as NR_3_ is less hindered and more basic.

An extended Hückel calculation was carried out on Me-DPA-mea in both closed and opened benzoxazine forms, i.e., as a precursor or polymerized, respectively.^54^ NR_3_ in Me-DPA-mea benzoxazine monomers has a less negative charge for the nitrogen atom compared to its opened form in the polymerized system (Table S4). The differences can be attributed to the repulsive interactions and steric effect within a closed benzoxazine ring. The lower electron density of the nitrogen atom in opened benzoxazine structures makes the electron lone pair more favorable to form intramolecular hydrogen bonds with hydroxyl groups. For instance, the proximity between the phenolic −OH and NR_3_ groups is suggested, by the cross-peak in the ^1^H–^15^N HMBC spectrum (δ_N/H_ = 12.4/5.85 ppm), to form a six-membered ring intramolecular hydrogen bond (Figure S45). It is also worth mentioning that the involvement of the substantial number of hydroxyl groups in hydrogen bonds enhances the system’s dynamicity by augmenting the electrophilicity of ester bonds and their trend to exchange with −OH groups.^33,55^ While this finding offers additional spectroscopic evidence of one of the most prevalent hydrogen-bonding interactions in benzoxazine,^56^ comprehensive computational studies need to be conducted to understand their prominent role in the polybenzoxazine vitrimer. The role of NR_3_ in transesterification reactions can be demonstrated through chemical interactions. This can be distinguished by the role of immobilized NR_3_ catalysts in model molecules, such as monoethanolamine and di- or triethylene glycol amine. Both types of model molecules release methanol, as shown in Figure S43. However, their kinetic behavior differs. The observed rate enhancement in Me-PA-mea for TER arises from the NGP of the tertiary amine in the β-position relative to the aliphatic −OH group, as evidenced by TGA-μGC analysis in Figure S43.

**Scheme 2:**
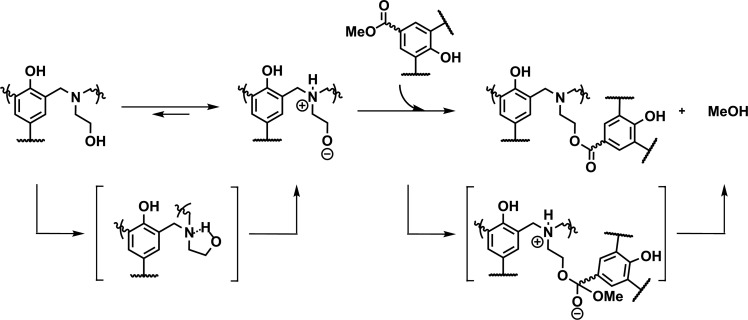
Proposed Mechanism for the NGP-Assisted Transesterification Reactions of the Benzoxazine Vitrimer Precursor Prepared with β-Amino-alcohol

In terms of a proton transfer exchange mechanism, transesterification in these systems is illustrated in Scheme 2 and can be explained as follows:

1. The less hindered and more basic NR_3_ in polybenzoxazine acts as a base catalyst, forming a zwitterionic intermediate through the internal cyclization process, resulting in an intermediate 5-membered cyclic N,O-acetal structure.
2. The conjugated alkoxide anion then adds to the carbonyl bond through an associative process.
3. Proton transfer exchange takes place during the elimination step, leading to the regeneration of an ester bond and methanol.

**Figure 7:**
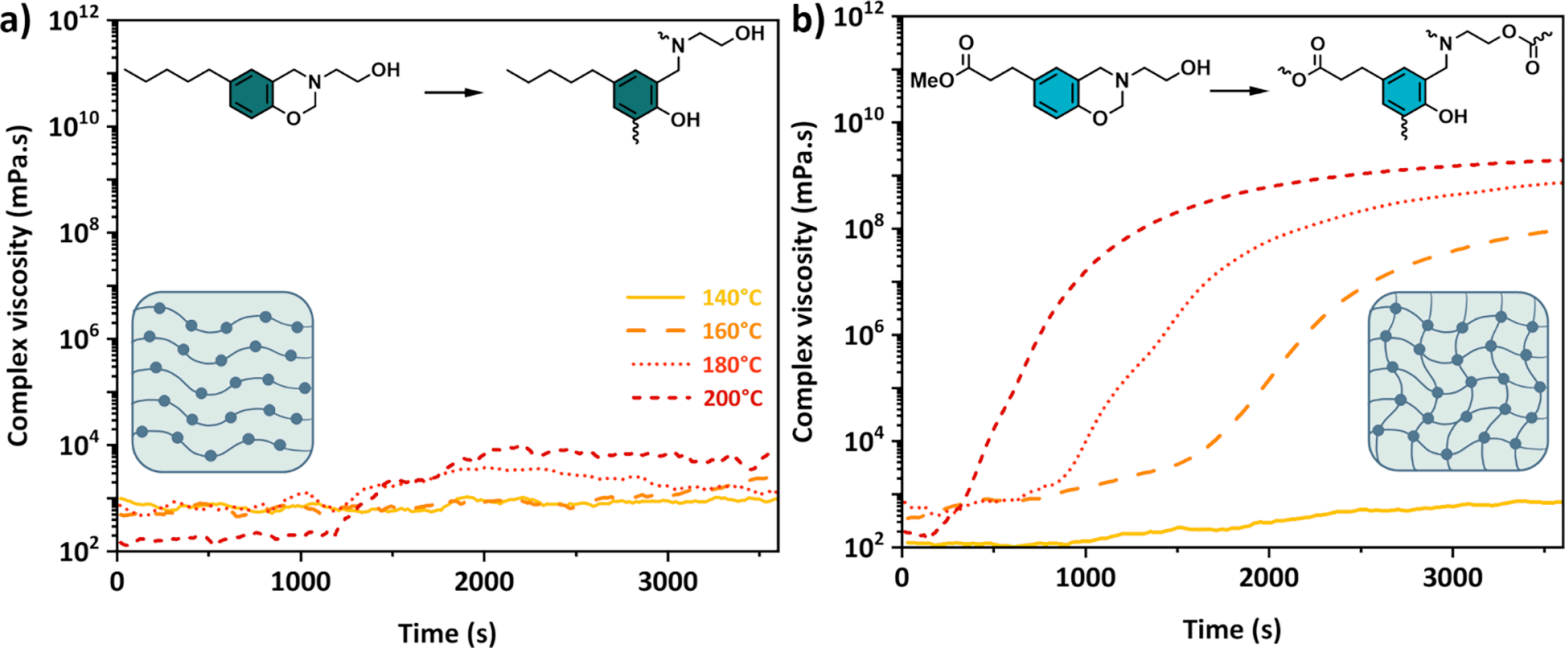
Isothermal evolution of the complex viscosity as a function of time of (a) *p*PP-mea (linear oligomer) and (b) Me-PA-mea (three-dimensional network).

In summary, NR_3_ within closed-benzoxazine structures is hindered and remains dormant until it was subjected to thermal treatment that triggers their opening. As illustrated in Figure 5, the release of methanol, which probes the occurrence of the reaction of transesterification in the model molecules, only occurs after the opening of the benzoxazine rings. Eventually, this process results in the generation of a stronger amine base compared to its closed-form counterpart. If the tertiary amine is inactive (e.g., weak base in a closed benzoxazine monomer), transesterification reactions do not occur, even at high temperatures (Figure 4, *T* = 130 °C). Similar thermal-latency findings were reported by Schlögl and colleagues.^57^ The two model molecules *p*PP-mea and Me-PA-mea effectively demonstrate the mechanism of cross-linking and structural changes through TER, as illustrated in Figure 7. Although the ROP of *p*PP-mea is accelerated by aliphatic −OH groups (Figure S21), its complex viscosity remains constant across the range of tested temperatures (Figure 7a). This is a known phenomenon, as the polymerization of monofunctional benzoxazine results in oligomers.^35,58^ In contrast, the temperature dependence of the complex viscosity for Me-PA-mea differs from that of *p*PP-mea, as shown in Figure 7b. Me-PA-mea remains unchanged below 140 °C, but above 150 °C, the benzoxazine ring opens, generating stronger and less hindered NR_3_ that catalyzes the TER. The improvement in complex viscosity from 160 °C indicates the formation of a network, which is only possible if TER occurs because of polycondensation between its methyl ester and −OH groups. This phenomenon only occurs after the opening of the benzoxazine rings. In contrast, Me-PA-fa and *p*PP-fa, which cannot undergo the transesterification reaction, do not cross-link (Figures S46 and S47, respectively). Their increase in complex viscosity at 200 °C is due to the involvement of furan rings in the cross-linking reaction.^58^ By the use of a monofunctional benzoxazine precursor containing ester bonds and aliphatic −OH groups, a cross-linked network can be constructed. The accelerated rate of polymerization of the benzoxazine monomer arises from the synergetic NGP of aliphatic −OH groups, which decreases the *E*_a_ of the benzoxazine ROP, and the involvement of transesterification reactions in building the three-dimensional network. This irreversible process results in the formation of a permanent poly(benzoxazine-*co*-ester) network devoid of aliphatic −OH groups.

## Conclusions

Benzoxazine-based vitrimers relying on TERs are characterized by the high thermal and mechanical properties of benzoxazine, combined with the molecular dynamicity of the covalent adaptable network. These systems are composed of aliphatic −OH, ester bonds, and tertiary amines, which interact with each other during the polymerization and transesterification reactions. Although the role of some functional groups, such as the tertiary amine of benzoxazine, is predictable, the underlying mechanisms are not thoroughly understood, which restricts the options to fine-tune the chemical structures of these systems. This research examines the intricate details of benzoxazine ring-opening polymerization and transesterification reactions within benzoxazine vitrimers. The aliphatic −OH of the β-amino-alcohol, which is crucial for TER to occur, also plays a catalytic role in benzoxazine ROP. The DSC kinetic studies in this research show a decrease in the barrier energy required for this reaction to take place. Moreover, TGA-μGC experiments and ^1^H–^15^N spectroscopic correlations reveal that transesterification reactions occur only when benzoxazine rings are opened on the model molecules.

These reactions are accelerated by the tertiary amine generated from ROP, which reduces the energy barrier needed for its activation. This is particularly noteworthy when the tertiary amine is involved in NGP with aliphatic −OH. In summary, the main findings of this study are as follows:

1. The aliphatic −OH in the β-position of an amino-alcohol accelerates benzoxazine ROP.
2. Transesterification does not occur when the benzoxazine rings are closed in these model molecules.
3. The β-amino-alcohol is involved in NGP during TER, leveraging the expected catalytic effect of the tertiary amine.
4. Benzoxazine rings act as a thermally latent self-catalyst for TER.

The invaluable insights into the mechanisms governing transesterification reactions internally catalyzed by tertiary amines shed more light on their crucial role in the kinetics of the dynamic exchanges. The present findings and method are not limited to the chemistry of benzoxazines but can be extrapolated to any kind of vitrimer.

## Supporting Information Available

Additional structural characterizations, DSC monitoring, conversion rates, TGA-μGC thermograms, and rheology analysis (PDF)

## Abbreviations

CANs: covalent adaptable networks
NMR: nuclear magnetic resonance
DSC: differential scanning calorimetry
TGA-μGC: thermogravimetric analysis coupled with microcomputed gas chromatography
NGP: neighboring group participation
TER: transesterification reaction
NR_3_: tertiary amine
PBZ: polybenzoxazine
ROP: ring-opening polymerization
HMBC: heteronuclear multiple bond correlation
PA: phloretic acid
DPA: diphenolic acid
*p*HBA: *para*-hydroxy benzoic acid
mea: monoethanolamine
dga: diethylene glycol amine
tga: triethylene glycol amine
fa: furfurylamine
*p*PP: 4-pentylphenol
*T*_onset_: onset temperature
FWO: Flynn–Wall–Ozawa
MS: mass spectroscopy
–OH: hydroxyl
*E*_aROP_: average activation energy of ring-opening polymerization
*E*_αROP_: apparent activation energy of ring-opening polymerization

## References

[ref1] Zou W.; Dong J.; Luo Y.; Zhao Q.; Xie T. Dynamic Covalent Polymer Networks: from Old Chemistry to Modern Day Innovations. Adv. Mater. 2017, 29 (14), 1606100 10.1002/adma.201606100. 28221707

[ref2] Denissen W.; Winne J. M.; Du Prez F. E. Vitrimers: permanent organic networks with glass-like fluidity. Chem. Sci. 2016, 7 (1), 30–38. 10.1039/C5SC02223A. 28757995 PMC5508697

[ref3] Montarnal D.; Capelot M.; Tournilhac F.; Leibler L. Silica-Like Malleable Materials from Permanent Organic Networks. Science (New York, N.Y.) 2011, 334, 965–968. 10.1126/science.1212648. 22096195

[ref4] Capelot M.; Montarnal D.; Tournilhac F.; Leibler L. Metal-Catalyzed Transesterification for Healing and Assembling of Thermosets. J. Am. Chem. Soc. 2012, 134 (18), 7664–7667. 10.1021/ja302894k. 22537278

[ref5] Capelot M.; Unterlass M. M.; Tournilhac F.; Leibler L. Catalytic Control of the Vitrimer Glass Transition. ACS Macro Lett. 2012, 1 (7), 789–792. 10.1021/mz300239f. 35607118

[ref6] Liu T.; Zhao B.; Zhang J. Recent development of repairable, malleable and recyclable thermosetting polymers through dynamic transesterification. Polymer 2020, 194, 122392 10.1016/j.polymer.2020.122392.

[ref7] Niu X.; Wang F.; Li X.; Zhang R.; Wu Q.; Sun P. Using Zn2+ Ionomer To Catalyze Transesterification Reaction in Epoxy Vitrimer. Ind. Eng. Chem. Res. 2019, 58 (14), 5698–5706. 10.1021/acs.iecr.9b00090.

[ref8] Van Zee N. J.; Nicolaÿ R. Vitrimers: Permanently crosslinked polymers with dynamic network topology. Prog. Polym. Sci. 2020, 104, 101233 10.1016/j.progpolymsci.2020.101233.

[ref9] Van Lijsebetten F.; Holloway J. O.; Winne J. M.; Du Prez F. E. Internal catalysis for dynamic covalent chemistry applications and polymer science. Chem. Soc. Rev. 2020, 49 (23), 8425–8438. 10.1039/D0CS00452A. 33112323

[ref10] Cuminet F.; Caillol S.; Dantras É.; Leclerc É.; Ladmiral V. Neighboring Group Participation and Internal Catalysis Effects on Exchangeable Covalent Bonds: Application to the Thriving Field of Vitrimer Chemistry. Macromolecules 2021, 54 (9), 3927–3961. 10.1021/acs.macromol.0c02706.

[ref11] Han J.; Liu T.; Hao C.; Zhang S.; Guo B.; Zhang J. A Catalyst-Free Epoxy Vitrimer System Based on Multifunctional Hyperbranched Polymer. Macromolecules 2018, 51 (17), 6789–6799. 10.1021/acs.macromol.8b01424.

[ref12] Liu T.; Zhang S.; Hao C.; Verdi C.; Liu W.; Liu H.; Zhang J. Glycerol Induced Catalyst-Free Curing of Epoxy and Vitrimer Preparation. Macromol. Rapid Commun. 2019, 40 (7), 1800889 10.1002/marc.201800889. 30721569

[ref13] Altuna F. I.; Pettarin V.; Williams R. J. J. Self-healable polymer networks based on the cross-linking of epoxidised soybean oil by an aqueous citric acid solution. Green Chem. 2013, 15 (12), 3360–3366. 10.1039/c3gc41384e.

[ref14] Berne D.; Cuminet F.; Lemouzy S.; Joly-Duhamel C.; Poli R.; Caillol S.; Leclerc E.; Ladmiral V. Catalyst-Free Epoxy Vitrimers Based on Transesterification Internally Activated by an α–CF3 Group. Macromolecules 2022, 55 (5), 1669–1679. 10.1021/acs.macromol.1c02538.

[ref15] Berne D.; Quienne B.; Caillol S.; Leclerc E.; Ladmiral V. Biobased catalyst-free covalent adaptable networks based on CF3-activated synergistic aza-Michael exchange and transesterification. Journal of Materials Chemistry A 2022, 10 (47), 25085–25097. 10.1039/D2TA05067F.

[ref16] Delahaye M.; Winne J. M.; Du Prez F. E. Internal Catalysis in Covalent Adaptable Networks: Phthalate Monoester Transesterification As a Versatile Dynamic Cross-Linking Chemistry. J. Am. Chem. Soc. 2019, 141 (38), 15277–15287. 10.1021/jacs.9b07269. 31469270

[ref17] Zhang H.; Majumdar S.; van Benthem R. A. T. M.; Sijbesma R. P.; Heuts J. P. A. Intramolecularly Catalyzed Dynamic Polyester Networks Using Neighboring Carboxylic and Sulfonic Acid Groups. ACS Macro Lett. 2020, 9 (2), 272–277. 10.1021/acsmacrolett.9b01023. 35638690

[ref18] Delahaye M.; Tanini F.; Holloway J. O.; Winne J. M.; Du Prez F. E. Double neighbouring group participation for ultrafast exchange in phthalate monoester networks. Polym. Chem. 2020, 11 (32), 5207–5215. 10.1039/D0PY00681E.

[ref19] Taplan C.; Guerre M.; Du Prez F. E. Covalent Adaptable Networks Using β-Amino Esters as Thermally Reversible Building Blocks. J. Am. Chem. Soc. 2021, 143 (24), 9140–9150. 10.1021/jacs.1c03316. 34121401

[ref20] Altuna F. I.; Hoppe C. E.; Williams R. J. J. Epoxy vitrimers with a covalently bonded tertiary amine as catalyst of the transesterification reaction. Eur. Polym. J. 2019, 113, 297–304. 10.1016/j.eurpolymj.2019.01.045.

[ref21] Li Y.; Liu T.; Zhang S.; Shao L.; Fei M.; Yu H.; Zhang J. Catalyst-free vitrimer elastomers based on a dimer acid: robust mechanical performance, adaptability and hydrothermal recyclability. Green Chem. 2020, 22 (3), 870–881. 10.1039/C9GC04080C.

[ref22] Hayashi M. Dominant Factor of Bond-Exchange Rate for Catalyst-Free Polyester Vitrimers with Internal Tertiary Amine Moieties. ACS Applied Polymer Materials 2020, 2 (12), 5365–5370. 10.1021/acsapm.0c01099.

[ref23] Xu Y.; Dai S.; Bi L.; Jiang J.; Zhang H.; Chen Y. Catalyst-free self-healing bio-based vitrimer for a recyclable, reprocessable, and self-adhered carbon fiber reinforced composite. Chemical Engineering Journal 2022, 429, 132518 10.1016/j.cej.2021.132518.

[ref24] Wang H.; Guo S.; Zhang X.; Liu Y.; Liu T.; Yu H. Insight into the structure-property relationships of intramolecularly-catalyzed epoxy vitrimers. Materials & Design 2022, 221, 110924 10.1016/j.matdes.2022.110924.

[ref25] Wanghofer F.; Wolfberger A.; Oreski G.; Schlögl S. Assessment of Epoxy Functionalized Poly(dimethylsiloxane) Vitrimers Catalyzed with Covalently Attached Amines as Reversible Adhesives. Macromol. Mater. Eng. 2022, 307 (9), 2200237 10.1002/mame.202200237.

[ref26] Zhang S.; Liu T.; Hao C.; Mikkelsen A.; Zhao B.; Zhang J. Hempseed Oil-Based Covalent Adaptable Epoxy-Amine Network and Its Potential Use for Room-Temperature Curable Coatings. ACS Sustainable Chem. Eng. 2020, 8 (39), 14964–14974. 10.1021/acssuschemeng.0c05223.

[ref27] Cao Q.; Li J.; Liu B.; Zhao Y.; Zhao J.; Gu H.; Wei Z.; Li F.; Jian X.; Weng Z. Toward versatile biobased epoxy vitrimers by introducing aromatic N-heterocycles with stiff and flexible segments. Chemical Engineering Journal 2023, 469, 143702 10.1016/j.cej.2023.143702.

[ref28] Hao C.; Liu T.; Zhang S.; Liu W.; Shan Y.; Zhang J. Triethanolamine-Mediated Covalent Adaptable Epoxy Network: Excellent Mechanical Properties, Fast Repairing, and Easy Recycling. Macromolecules 2020, 53 (8), 3110–3118. 10.1021/acs.macromol.9b02243.

[ref29] Hayashi M.; Inaba T. Achievement of a Highly Rapid Bond Exchange for Self-Catalyzed Polyester Vitrimers by Incorporating Tertiary Amino Groups on the Network Strands. ACS Applied Polymer Materials 2021, 3 (9), 4424–4429. 10.1021/acsapm.1c00724.

[ref30] Stricker L.; Taplan C.; Du Prez F. E. Biobased, Creep-Resistant Covalent Adaptable Networks Based on β-Amino Ester Chemistry. ACS Sustainable Chem. Eng. 2022, 10 (42), 14045–14052. 10.1021/acssuschemeng.2c04822.

[ref31] Jeon D.; Yoon Y.; Kim D.; Lee G.; Ahn S.-k.; Choi D.; Kim C. B. Fully Recyclable Covalent Adaptable Network Composite with Segregated Hexagonal Boron Nitride Structure for Efficient Heat Dissipation. Macromolecules 2023, 56 (2), 697–706. 10.1021/acs.macromol.2c01927.

[ref32] Bories-Azeau X.; Armes S. P. Unexpected Transesterification of Tertiary Amine Methacrylates during Methanolic ATRP at Ambient Temperature: A Cautionary Tale. Macromolecules 2002, 35 (27), 10241–10243. 10.1021/ma021388g.

[ref33] Adjaoud A.; Puchot L.; Verge P. Polybenzoxazine-based covalent adaptable networks: A mini-review. Polymer 2023, 287, 126426 10.1016/j.polymer.2023.126426.

[ref34] Adjaoud A.; Boina D. A.; Boulic V.; Hesse C.; Jehl C.; Ziane C.; Puchot L.; Shaplov A. S.; Schmidt D. F.; Verge P. Enhancing Sustainable Plastics: Introducing Bio-based Benzoxazines with Dynamic Bonds for Exceptional Performance and Circularity. In Sustainable Green Chemistry in Polymer Research. Vol. 2. Sustainable Polymers and Applications, ACS Symposium Series, Vol. 1451; American Chemical Society, 2023; pp 49–84.

[ref35] Ishida H. Chapter 1 - Overview and Historical Background of Polybenzoxazine Research. In Handbook of Benzoxazine Resins, Ishida H.; Agag T., Eds.; Elsevier, 2011; pp 3–81.

[ref36] Baqar M.; Agag T.; Qutubuddin S.; Ishida H. Chapter 10 - Effect of Neighboring Groups on Enhancing Benzoxazine Autocatalytic Polymerization. In Handbook of Benzoxazine Resins, Ishida H.; Agag T., Eds.; Elsevier, 2011; pp 193–210.

[ref37] Kudoh R.; Sudo A.; Endo T. A Highly Reactive Benzoxazine Monomer, 1-(2-Hydroxyethyl)-1,3-Benzoxazine: Activation of Benzoxazine by Neighboring Group Participation of Hydroxyl Group. Macromolecules 2010, 43, 1185–1187. 10.1021/ma902416h.

[ref38] Monisha M.; Sahu S.; Lochab B. Self-Polymerization Promoting Monomers: In Situ Transformation of Disulfide-Linked Benzoxazines into the Thiazolidine Structure. Biomacromolecules 2021, 22 (10), 4408–4421. 10.1021/acs.biomac.1c00981. 34582169

[ref39] Adjaoud A.; Trejo-Machin A.; Puchot L.; Verge P. Polybenzoxazines: a sustainable platform for the design of fast responsive and catalyst-free vitrimers based on trans-esterification exchanges. Polym. Chem. 2021, 12 (22), 3276–3289. 10.1039/D1PY00324K.

[ref40] Adjaoud A.; Puchot L.; Verge P. High-Tg and Degradable Isosorbide-Based Polybenzoxazine Vitrimer. ACS Sustainable Chem. Eng. 2022, 10 (1), 594–602. 10.1021/acssuschemeng.1c07093.

[ref41] Adjaoud A.; Puchot L.; Federico C. E.; Das R.; Verge P. Lignin-based benzoxazines: A tunable key-precursor for the design of hydrophobic coatings, fire resistant materials and catalyst-free vitrimers. Chemical Engineering Journal 2023, 453, 139895 10.1016/j.cej.2022.139895.

[ref42] Ning X.; Ishida H. Phenolic materials via ring-opening polymerization of benzoxazines: Effect of molecular structure on mechanical and dynamic mechanical properties. J. Polym. Sci., Part B: Polym. Phys. 1994, 32 (5), 921–927. 10.1002/polb.1994.090320515.

[ref43] Kiskan B.; Koz B.; Yagci Y. Synthesis and characterization of fluid 1,3-benzoxazine monomers and their thermally activated curing. J. Polym. Sci., Part A: Polym. Chem. 2009, 47 (24), 6955–6961. 10.1002/pola.23735.

[ref44] Wen Z.; Bonnaud L.; Mincheva R.; Dubois P.; Raquez J.-M. Development of Low-Viscosity and High-Performance Biobased Monobenzoxazine from Tyrosol and Furfurylamine. Materials 2021, 14 (2), 440 10.3390/ma14020440. 33477447 PMC7829698

[ref45] Kissinger H. E. Reaction Kinetics in Differential Thermal Analysis. Anal. Chem. 1957, 29 (11), 1702–1706. 10.1021/ac60131a045.

[ref46] Flynn J. H.; Wall L. A. General Treatment of the Thermogravimetry of Polymers. Journal of research of the National Bureau of Standards. Section A, Physics and chemistry 1966, 70A (6), 487–523. 10.6028/jres.070A.043. PMC6624709 31824016

[ref47] Ozawa T. Kinetic analysis of derivative curves in thermal analysis. Journal of thermal analysis 1970, 2 (3), 301–324. 10.1007/BF01911411.

[ref48] Friedman H. L. Kinetics of thermal degradation of char-forming plastics from thermogravimetry. Application to a phenolic plastic. Journal of Polymer Science Part C: Polymer Symposia 1964, 6 (1), 183–195. 10.1002/polc.5070060121.

[ref49] Fu F.; Huang M.; Zhang W.; Zhao Y.; Liu X. Thermally assisted self-healing behavior of anhydride modified polybenzoxazines based on transesterification. Sci. Rep. 2018, 8 (1), 10325 10.1038/s41598-018-27942-9. 29985408 PMC6037739

[ref50] Fedelich N. TGA-Micro GC/MS. In Evolved Gas Analysis 2019, 40−44 10.3139/9781569908105.

[ref51] Zhang W.; Froimowicz P.; Arza C. R.; Ohashi S.; Xin Z.; Ishida H. Latent Catalyst-Containing Naphthoxazine: Synthesis and Effects on Ring-Opening Polymerization. Macromolecules 2016, 49 (19), 7129–7140. 10.1021/acs.macromol.6b01177.

[ref52] Lin R.; Zhu Y.; Zhang Y.; Wang L.; Yu S. Pyrogallol-based benzoxazines with latent catalytic characteristics: The temperature-dependent effect of hydrogen bonds on ring-opening polymerization. Eur. Polym. J. 2018, 102, 141–150. 10.1016/j.eurpolymj.2018.03.015.

[ref53] Zhang K.; Han L.; Nie Y.; Szigeti M. L.; Ishida H. Examining the effect of hydroxyl groups on the thermal properties of polybenzoxazines: using molecular design and Monte Carlo simulation. RSC Adv. 2018, 8 (32), 18038–18050. 10.1039/C8RA02033G. 35542113 PMC9080532

[ref54] Neal E. A.; Werling A. Y. R.; Jones C. R. A simple Hückel model-driven strategy to overcome electronic barriers to retro-Brook silylation relevant to aryne and bisaryne precursor synthesis. Chem. Commun. 2021, 57 (13), 1663–1666. 10.1039/D0CC08283J. 33463642

[ref55] Wang S.; Teng N.; Dai J.; Liu J.; Cao L.; Zhao W.; Liu X. Taking advantages of intramolecular hydrogen bonding to prepare mechanically robust and catalyst-free vitrimer. Polymer 2020, 210, 123004 10.1016/j.polymer.2020.123004.

[ref56] Kim H.-D.; Ishida H. A Study on Hydrogen-Bonded Network Structure of Polybenzoxazines. J. Phys. Chem. A 2002, 106 (14), 3271–3280. 10.1021/jp010606p.

[ref57] Reisinger D.; Kriehuber M. U.; Bender M.; Bautista-Anguís D.; Rieger B.; Schlögl S. Thermally Latent Bases in Dynamic Covalent Polymer Networks and their Emerging Applications. Adv. Mater. 2023, 35 (24), 2300830 10.1002/adma.202300830. 36916976

[ref58] Trejo-Machin A.; Adjaoud A.; Puchot L.; Dieden R.; Verge P. Elucidating the thermal and polymerization behaviours of benzoxazines from lignin derivatives. Eur. Polym. J. 2020, 124, 109468 10.1016/j.eurpolymj.2019.109468.

