# Supplementary material for: Deciphering the Self-Catalytic Mechanisms of Polymerization and Transesterification in Polybenzoxazine Vitrimers
Source: J Am Chem Soc. 2024 May 2;146(19):13367–76. doi: 10.1021/jacs.4c02153 (PMC11100009; doi:10.1021/jacs.4c02153)
Supplement: Supplementary file 1 — ja4c02153_si_001.pdf [file ja4c02153_si_001.pdf]

ELECTRONIC SUPPORTING INFORMATION

Deciphering the self-catalytic mechanisms of polymerization and transesterification in polybenzoxazine vitrimers

Antoine Adjaoud [†], *Benoit Marcolini* [†], *Reiner Dieden* [†], *Laura Puchot* [†], *and Pierre Verge* [†, *]

[†] Luxembourg Institute of Science and Technology, Materials Research and Technology Department, 5 Avenue des Hauts-Fourneaux, L-4362 Esch-sur-Alzette (Luxembourg)

* Corresponding author: pierre.verge@list.lu

Experimental

Materials and chemicals: 3-(4-hydroxyphenyl) propionic acid (98 %, phloretic acid, PA), 4,4-bis(4-hydroxyphenyl)valeric acid (95 %, diphenolic acid, DPA), 4-hydroxybenzoic acid (99 %, *p*HBA), 4-pentylphenol (≥ 98 %, pPP), *para*-toluene sulfonic acid monohydrate (≥ 98.5 %, *p*TSA), 2-aminoethanol (> 98 %, monoethanolamine, mea), 2-(2-aminoethoxy)ethanol (98 %, diethylene glycolamine, dga), 2-aminomethylfuran (99 %, furfurylamine, fa), and paraformaldehyde (95 %, PFA) were purchased from Sigma-Aldrich®. Extra pure methanol (≥ 99.5 %, Me) was purchased from Fischer Chemical™. 2-[2-(2-aminoethoxy)ethoxy]ethanol (97%, triethylene glycolamine, tga) was purchased from AK Scientific. All solvents and chemicals were used as received without any purification.

Synthetic procedure: A typical procedure for the synthesis of model molecules is reported in Scheme 1. First, methyl-ester derivatives were synthesized *via* the Fischer esterification of phenolic acid precursors (PA, DPA, pHBA) with an excess of methanol in the presence of *p*TSA as an acid catalyst (0.5 wt.%). After 12 h of reaction at the reflux temperature of methanol, the reaction media was cooled to room temperature and concentrated under reduced pressure. Then, the extract was redissolved in chloroform (Me-PA) or butanone (Me-DPA and Me-*p*HBA) and purified by three liquid−liquid extractions with 5% sodium bicarbonate ($NaHCO_3$), followed by three liquid−liquid extractions with ultrapure water (H_2O, Merck Millipore Milli-Q™). The organic layer was dried over magnesium sulfate ($MgSO_4$), evaporated under reduced pressure, and the product was recovered as fine powder. In a second step, methyl-ester precursors (Me-PA, Me-DPA, Me-*p*HBA, 1.0 equiv) were reacted solventless with paraformaldehyde (PFA, 2.0 or 4.0 equiv), and an amino-alcohol (mea, dga, tga, 1.0 or 2.0 equiv) or an amino-furan (fa, 1.0 or 2.0 equiv) derivative at 70°C for 3−24 h under mechanical stirring (Ministar 20 Digital, 250 rpm). The synthesis of ester-free model molecules from *p*PP follows a similar procedure without reacting through Fischer esterification. All model molecules were thoroughly dried over $MgSO_4$ and used without any purification. All these model molecules were obtained in high yields as viscous liquid (Me-PA-mea/dga/tga/fa and pPP-mea/fa), wax (Me-PHBA-mea/fa), or solid powder (Me-DPA-mea/fa).

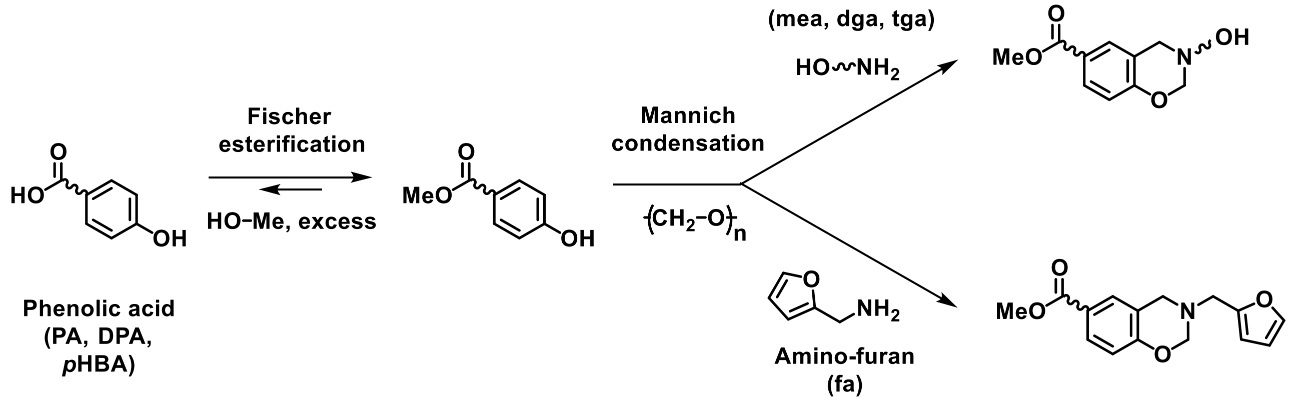

Scheme S1 *Synthesis of benzoxazine vitrimer model molecules through consecutive Fischer esterification and Mannich-like condensation.*

Kinetic of benzoxazine ring-opening polymerization: The activation energy of benzoxazine ROP was evaluated through non-isothermal differential scanning calorimetry (DSC) experiments at the following heating rates: 2/5/10/15/20°C·min^{-1}. The average Ea of benzoxazine ROP (Ea_{ROP}) was determined using Kissinger[1] (eq. 1) and Flynn−Wall−Ozawa[2, 3] (FWO, eq. 2) methods:

$$(1)\ \ln\left(\frac{\beta}{Tp^2}\right) = \ln\left(\frac{A*R}{Ea}\right) - \frac{Ea}{R*Tp} \qquad (2)\ \ln(\beta) = C - 1.052*\left(\frac{Ea}{R*Tp}\right)$$

where Ea corresponds to the average activation energy, Tp is the peak of the first exothermic transition, A is the frequency factor, β the heating rate of the curing reaction, R is the gas constant, and c a constant. The Ea can be obtained from the slope of the linear regression of $\ln(\frac{\beta}{Tp^2})$ versus $\frac{1}{Tp}$ (eq.1) or $\ln(\beta)$ versus $\frac{1}{Tp}$ (eq.2).

The apparent Ea of benzoxazine ROP ($E\alpha_{ROP}$) was determined using a numerical optimization of the isoconversional Friedman analysis[4]. As this method is closely related to the conversion rate, a deconvolution of the first exothermic peak was performed using a Gaussian fitting function on OriginPro version 2019b (eq.3):

$$(3)\ y = y_0 + \frac{Ae^\wedge\left(\frac{-4\ln(2)(x-x_c)^2}{w^2}\right)}{w\sqrt{\frac{\pi}{4ln(2)}}}$$

The isoconversional Freidman method calculates the dependence of activation energy on the degree of conversion (α, eq.4).

$$(4)\ \ln\left(\frac{d\alpha}{dT}\right) = \ln\left(Af(\alpha)\right) - \frac{E\alpha}{R*Tp}$$

The Eα can be obtained from the slope of the linear regression of $\ln(\frac{d\alpha}{dT})$ versus $\frac{1}{Tp}$ (eq.4).The model-free numerical optimization implemented by NETZSCH Kinetics Neo (Professional Edition, product version 1.2.6.2.) was used for the accurate calculations of $E\alpha_{ROP}$ from the deconvoluted peaks. The method is based on the optimization of the results of the Friedman analysis (sum of squares of deviations between measured and simulated value) and intends to minimize the optimization function while the iterations are repeated until no any numerical improvements happens. The $E\alpha_{ROP}$ was calculated as the average mean between the conversion rate of 0.2 and 0.8.

TGA-µGC: A thermogravimetric analysis coupled with micro–gas chromatography apparatus (TGA-µGC) was used to identify and quantify the gaseous methanol stream adduct of transesterification reactions[3]. The crucible containing 10 mg of sample is directly inserted at the isotherm temperature and 50 micro GC scans of 120 s were acquired spread over the entire TGA test (4 h). For the quantitative analysis, the analyser was calibrated using a standard methanol sample. The integration of the area of the alcohol peak is normalized to the methanol standard and is used to monitor the progress of transesterification reactions. Details on the retention time of the carrier gas and standard gas sample used for the calibration are provided in Table S2.

^{1}H and ^{1}H-^{15}N HMBC NMR kinetic experiment: The sample was mixed in deuterated dimethyl sulfoxide at a concentration of 0.9 M (200 mg in 0.6 mL of DMSO-d_6). The mixture was homogenized by vortex stirring and

550 mL were transferred into an NMR tube. The kinetic of thermally-induced reactions was monitored at 140ºC overnight using an AVANCE III HD Bruker spectrometer operating at a proton frequency of 600 MHz. Conditions for ^{1}H NMR experiment: spectrum recorded every 5 minutes, 16 scans, 2.72 s acquisition time, 1 s relaxation delay. Conditions for ^{1}H-^{15}N HMBC NMR experiment: spectrum recorded every 39 minutes, 8 scans, 0.13 s acquisition time in ^{1}H dimension, 5.27 µs acquisition time in ^{15}N dimension, 1 s relaxation delay).

Computational calculations: Model structures were drawn in ChemDraw® software (PerkinElmer Informatics, version 19.0.0.22). The molecular modelling was visualized in the Chem3D plugin and energy was minimized using the implementation of MM2. The calculation of the partial charge density was performed using the Extended Hückel method and the algorithm provided therein.

Equipment and characterizations

Nuclear Magnetic Resonance (NMR) spectroscopy was performed on an AVANCE III HD Bruker spectrometer equipped with a 5 mm BBO-probe operating at a proton frequency of 600 MHz. All chemical shifts are given as δ value (ppm) referenced to tetramethyl silane (TMS) as an internal standard. Assignments were performed using a combination of COSY, HSQC, and HMBC spectra. Peak multiplicity was indicated as follows: singlet (s); doublet (d); triplet (t) or multiplet (m). The coupling constants (J) were reported in Hertz (Hz).

Differential scanning calorimetry (DSC) thermograms were recorded on a Netzsch DSC 204 F1 Phoenix device in standard pierced aluminum crucibles (40 µL) and a sample mass of 5 mg. A linear heating ramp at a constant heating rate was applied from 25 to 300°C under a nitrogen flow rate (N_2, 40 $mL.min^{-1}$).

Thermogravimetric analysis coupled with micro-gas chromatography (TGA- micro GC) was completed on the Mettler Toledo TGA 2 device coupled with SOLIA 490 micro-GC. TGA experiment was conducted in a ceramic alumina pan in isothermal conditions under an inert atmosphere (N_2, 20 $mL.min^{-1}$). The micro GC apparatus includes three analytical modules equipped with a micro thermal conductivity detector (10 µL injection time). The relative integration of the methanol peak' area was performed on module C using Soprane II software. Each data point corresponds to an individual micro-GC injection.

Table S1 *Retention time of the carrier gas and methanol standard on the micro GC column.*

Module	Retention time carrier gas (s)	Retention time methanol standard (s)
A	39-39.2 / 39.7-40.2	35.18
B	17.8 / 63.1-63.4	105-107
C	16.3-16.4 / 19.3-19.5	21.9-22.1

Rheological measurements were recorded using an Anton Paar Physica MCR 302 rheometer equipped with a CTD 450 temperature control device. The isothermal rheo-kinetic measurements were performed using small quantities of the samples loaded in a parallel plate-plate geometry (Ø= 25 mm, gap 0.5 mm). The polymerization measurements were recorded in the oscillation mode at a controlled strain of 0.1% (1 Hz). Heating ramps of 20°C·min^{-1} were applied to reach the targeted temperature. The sample deformation was ramped linearly from 1% to 0.2% to remain within the instrument's limitation and to maintain a linear viscoelastic behavior as the moduli (G' storage modulus, G'' loss modulus) increase by several orders of magnitude upon curing.

1. Synthesis and structural characterization of the model molecules

➢ *Me-PA (Y= 73 % after purification)*

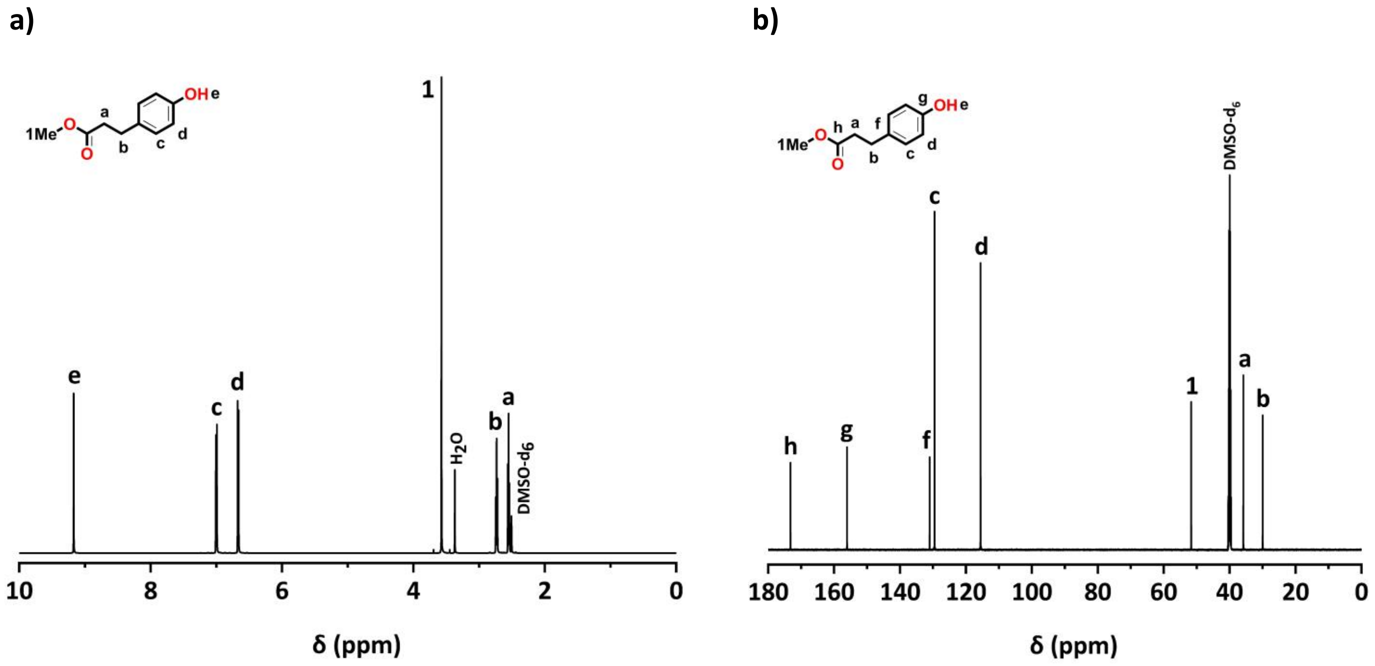

Figure S1 *a) ^{1}H and b) ^{13}C NMR spectra of Me-PA.*

^{1}H NMR (DMSO-d_6, 600 MHz, 298°K): δ (ppm) = (assignment, multiplicity (coupling constant), [attribution], experimental integration, theoretical integration). δ= 2.55 (CH_2-CH_2*-C=O, t (J = 7.70 Hz), [a], exp 2.00H, th 2.00H); δ= 2.73 (CH_2-CH_2*-Ar, t (J = 7.61 Hz), [b], exp 2.01H, th 2.00H); δ= 3.57 (CH_3*-O-C=O, s, [1], exp 3.00H, th 3.00H); δ= 6.66 (CH=CH*-C-OH, d (J = 8.45 Hz), [d], exp 1.98H, th 2.00H); δ= 6.99 (C-C-CH*=CH, d (J = 8.45 Hz), [c], exp 1.98H, th 2.00H); δ= 9.17 (Ar-OH*, s, [e], exp 0.99H, th 1.00H).

^{13}C NMR (DMSO-d_6, 600 MHz, 298°K): δ (ppm) = 30.0 [b]; 35.8 [a]; 51.7 [1]; 115.6 [d]; 129.5 [c]; 131.0 [f]; 156.1 [g]; 173.2 [h].

- *<u>Me-DPA (Y= 86 % after purification)</u>*

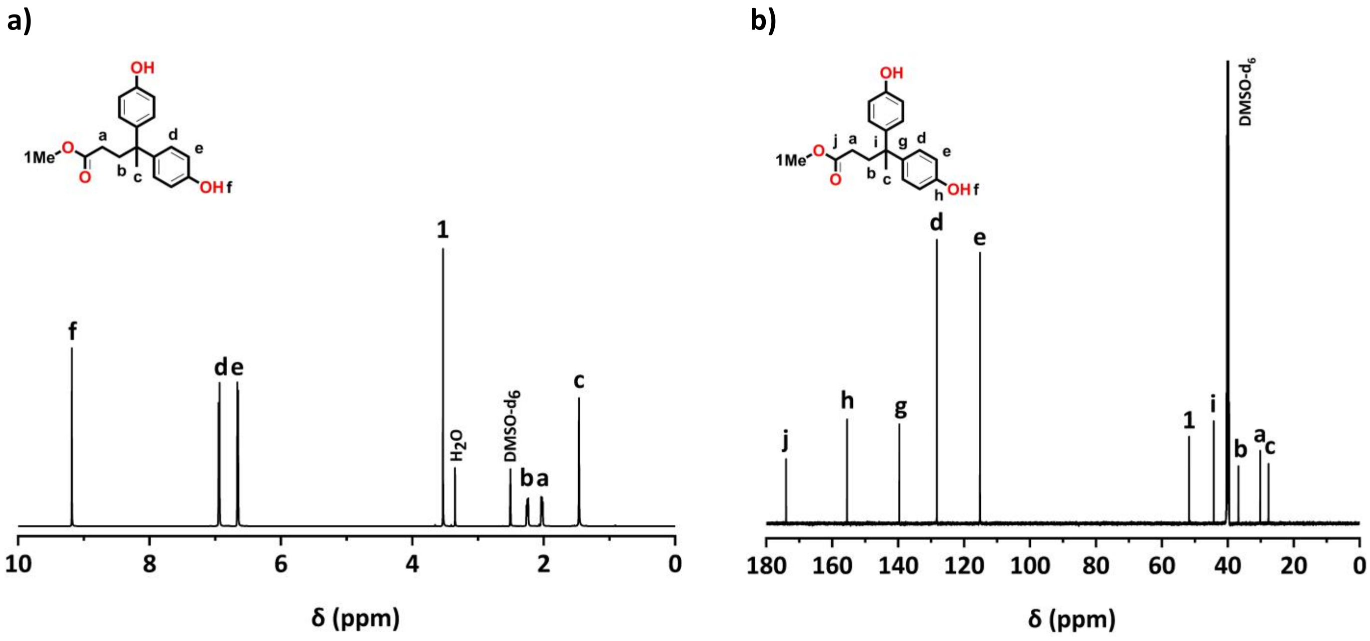

Figure S2 *a) ¹H and b) ¹³C NMR spectra of Me-DPA.*

¹H NMR (DMSO-d6, 600 MHz, 298°K): δ (ppm) = (assignment, multiplicity (coupling constant), [attribution], experimental integration, theoretical integration). δ= 1.46 (CH_3*-C, s, [c], exp 3.00H, th 3.00H); δ= 2.03 (CH_2-CH_2*-C=O, t (J = 8.19 Hz), [a], exp 2.02H, th 2.00H); δ= 2.25 (CH_2-CH_2*-C, t (J = 8.18 Hz), [b], exp 2.00H, th 2.00H); δ= 3.53 (CH_3*-O-C=O, s, [1], exp 3.00H, th 3.00H); δ= 6.65 (CH=CH*-C-OH, d (J = 8.72 Hz), [e], exp 4.00H, th 4.00H); δ= 6.94 (C-C-CH*=CH, d (J = 8.72 Hz), [d], exp 4.02H, th 4.00H); δ= 9.18 (Ar-OH*, s, [f], exp 2.00H, th 2.00H).

¹³C NMR (DMSO-d6, 600 MHz, 298°K): δ (ppm) = 27.7 [c]; 30.2 [a]; 36.8 [b]; 44.3 [i]; 51.7 [1]; 115.2 [e]; 128.2 [d]; 139.6 [g]; 155.5 [h]; 174.0 [j].

- *Me-pHBA (Y= 84 % after purification)*

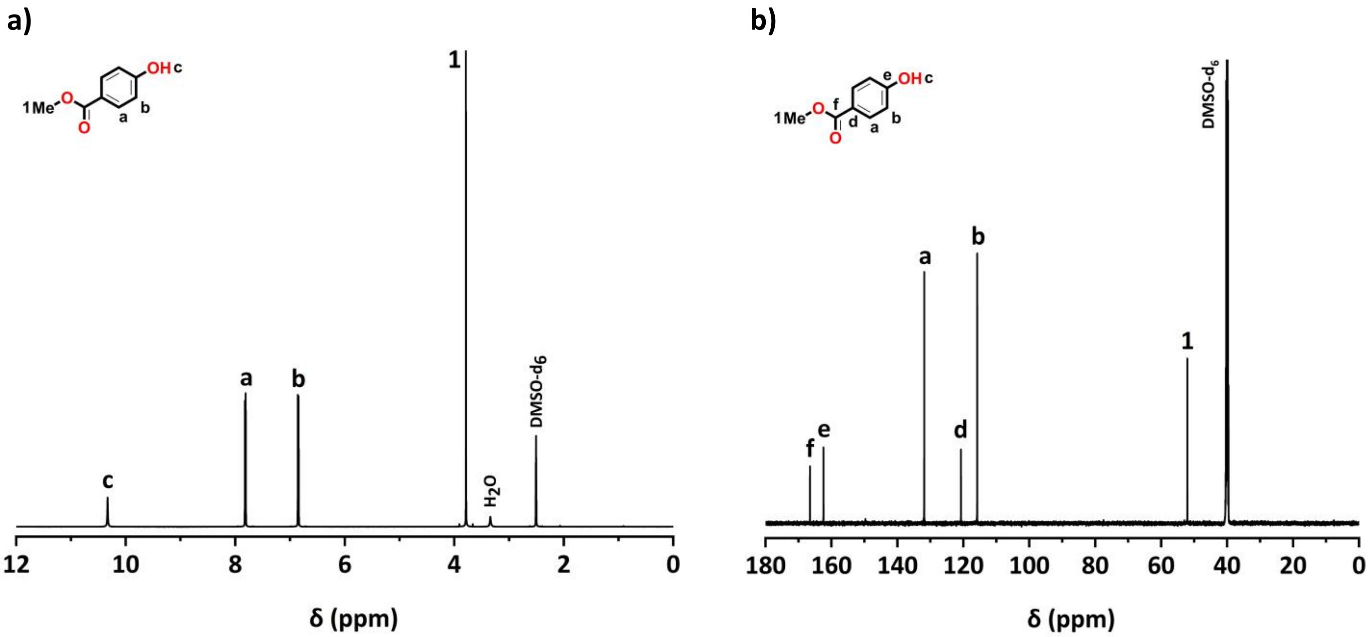

Figure S3 *a) ^{1}H and b) ^{13}C NMR spectra of Me-pHBA.*

^{1}H NMR (DMSO-d$_6$, 600 MHz, 298°K): δ (ppm) = (assignment, multiplicity (coupling constant), [attribution], experimental integration, theoretical integration). δ= 3.79 (CH$_3$*-O-C=O, s, [1], exp 3.00H, th 3.00H); δ= 6.85 (CH=CH*-C-OH, d (J= 8.71 Hz), [a], exp 2.00H, th 2.00H); δ= 7.81 (C-C-CH*=CH, d (J= 8.76 Hz), [b], exp 1.98H, th 2.00H); δ= 10.33 (Ar-OH*, s, [c], exp 0.94H, th 1.00H).

^{13}C NMR (DMSO-d$_6$, 600 MHz, 298°K): δ (ppm) = 52.1 [1]; 115.8 [b]; 120.7 [d]; 131.9 [a]; 162.4 [e]; 166.5 [f].

➢ <u>*Me-PA-mea*</u>

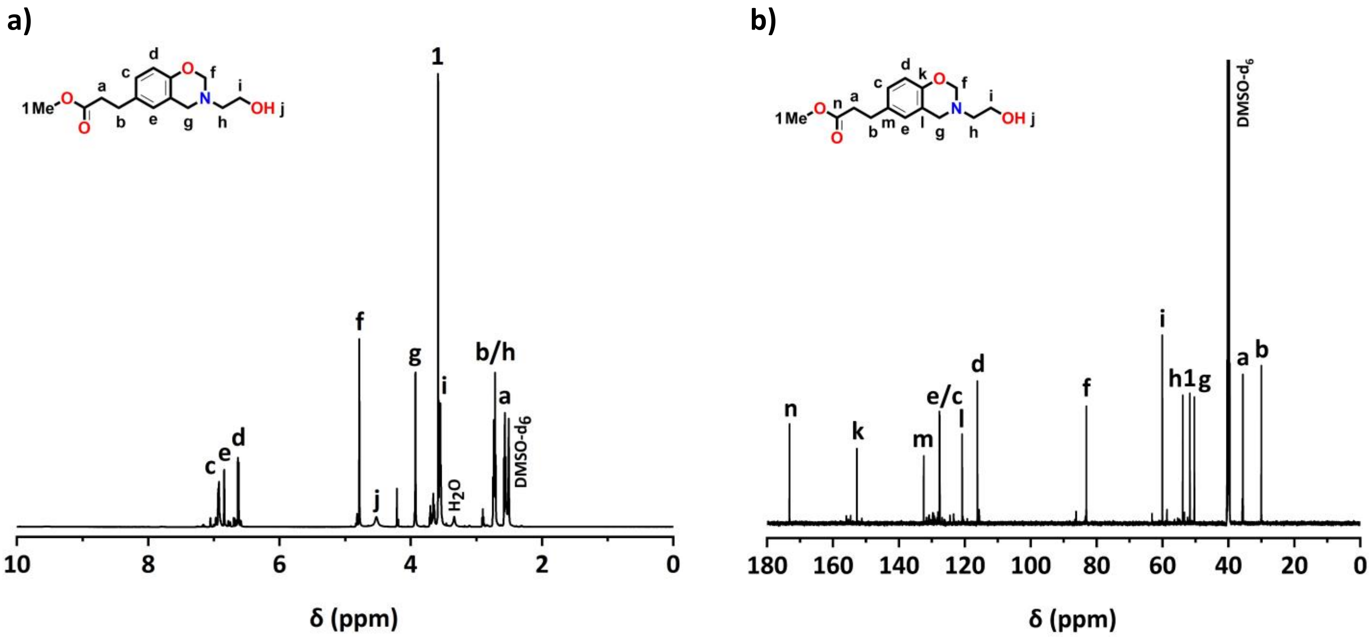

Figure S4 *a) ^{1}H and b) ^{13}C NMR spectra of Me-PA-mea.*

^{1}H NMR (DMSO-d_6, 600 MHz, 298°K): δ (ppm) = (assignment, multiplicity (coupling constant), [attribution], experimental integration, theoretical integration). δ= 2.57 (CH_2-CH_2*-C=O, t (J = 7.69 Hz), [a], exp 2.00H, th 2.00H); δ= 2.72 (CH_2-CH_2*-Ar & CH_2-CH_2*-N, m, [b&h], exp 3.73H, th 4.00H); δ= 3.55-3.58 (CH_3*-C=O & CH_2-CH_2*-OH, [1&i], exp 4.98H, th 5.00H); δ= 3.93 (Ar-CH_2*-N-, s, [g], exp 1.49H, th 2.00H); δ= 4.52 (CH_2-OH*, s, [j], exp 0.79H, th 1.00H); δ= 4.78 (O-CH_2*-N, s, [f], exp 1.50H, th 2.00H); δ= 6.62-6.93 (CH=CH*-C-O & C-CH*=C & C-CH*=CH, m, [d&e&c], exp 2.94H, th 3.00H).

^{13}C NMR (DMSO-d_6, 600 MHz, 298°K): δ (ppm) = 30.1 [b]; 35.7 [a]; 50.5 [g]; 51.8 [1]; 54.0 [h]; 60.1 [i]; 83.2 [f]; 116.3 [d]; 120.9 [l]; 127.7 [c]; 127.8 [e]; 132.6 [m]; 152.8 [k]; 173.3 [n].

- *Me-PA-dga*

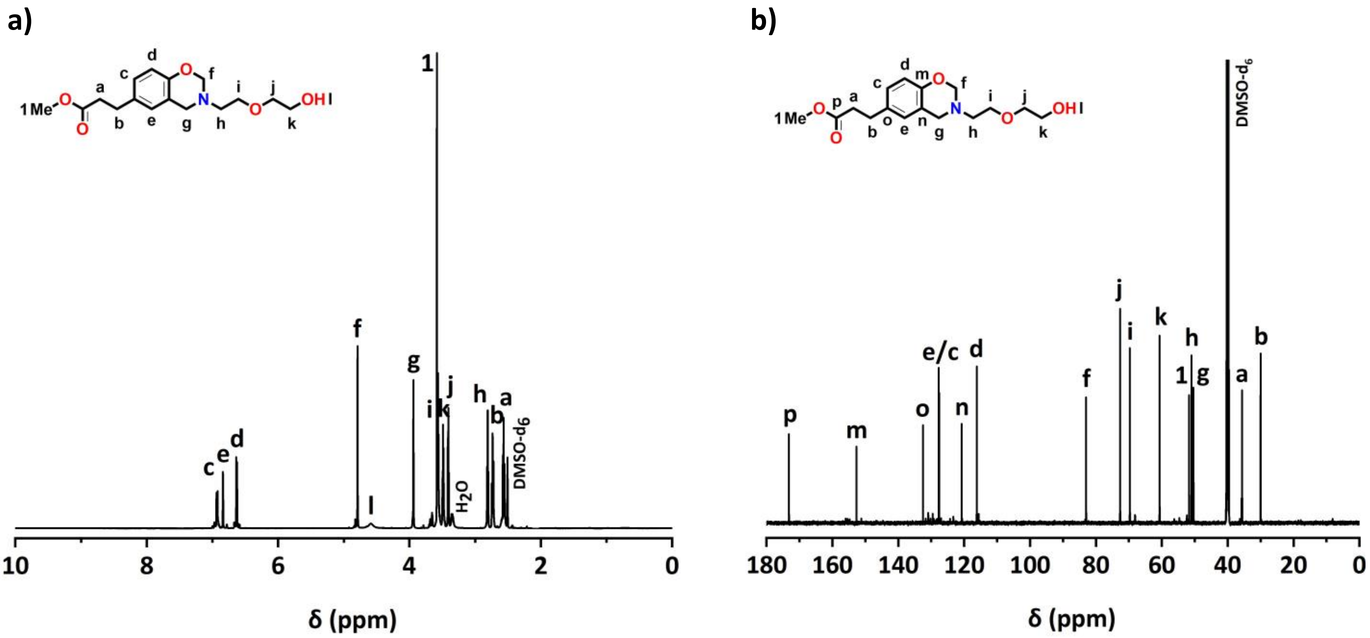

Figure S5 *a) ^{1}H and b) ^{13}C NMR spectra of Me-PA-dga.*

^{1}H NMR (DMSO-d_6, 600 MHz, 298°K): δ (ppm) = (assignment, multiplicity (coupling constant), [attribution], experimental integration, theoretical integration). δ= 2.57 (CH_2-CH_2*-C=O, t (J = 7.68 Hz), [a], exp 2.00H, th 2.00H); δ= 2.72 (CH_2-CH_2*-Ar, t (J= 7.67 Hz), [b], exp 2.00H, th 2.00H); δ= 2.80 (N-CH_2*-CH_2-O, t (J= 5.85 Hz), [h], exp 1.68H, th 2.00H); δ= 3.41 (O-CH_2*-CH_2-O, t (J= 5.18 Hz), [j], exp 1.81H, th 2.00H); δ= 3.49 (O-CH_2-CH_2*-OH, t (J= 5.18 Hz), [k], exp 2.02H, th 2.00H); δ= 3.56-3.58 (N-CH_2-CH_2*-O & CH_3*-C=O, [i&1], exp 4.90H, th 5.00H); δ= 3.94 (Ar-CH_2*-N-, s, [g], exp 1.59H, th 2.00H); δ= 4.59 (CH_2-OH*, s, [l], exp 0.77H, th 1.00H); δ= 4.79 (O-CH_2*-N, s, [f], exp 1.62H, th 2.00H); δ= 6.62-6.94 (CH=CH*-C-O & C-CH*=C & C-CH*=CH, m, [d&e&c], exp 2.96H, th 3.00H).

^{13}C NMR (DMSO-d_6, 600 MHz, 298°K): δ (ppm) = 30.0 [b]; 35.6 [a]; 50.4 [g]; 51.0 [h]; 51.7 [1]; 60.7 [k]; 69.7 [i]; 72.7 [j]; 83.0 [f]; 116.2 [d]; 120.8 [n]; 127.6 [c]; 127.7 [e]; 132.5 [o]; 152.7 [m]; 173.2 [p].

➢ *Me-PA-tga*

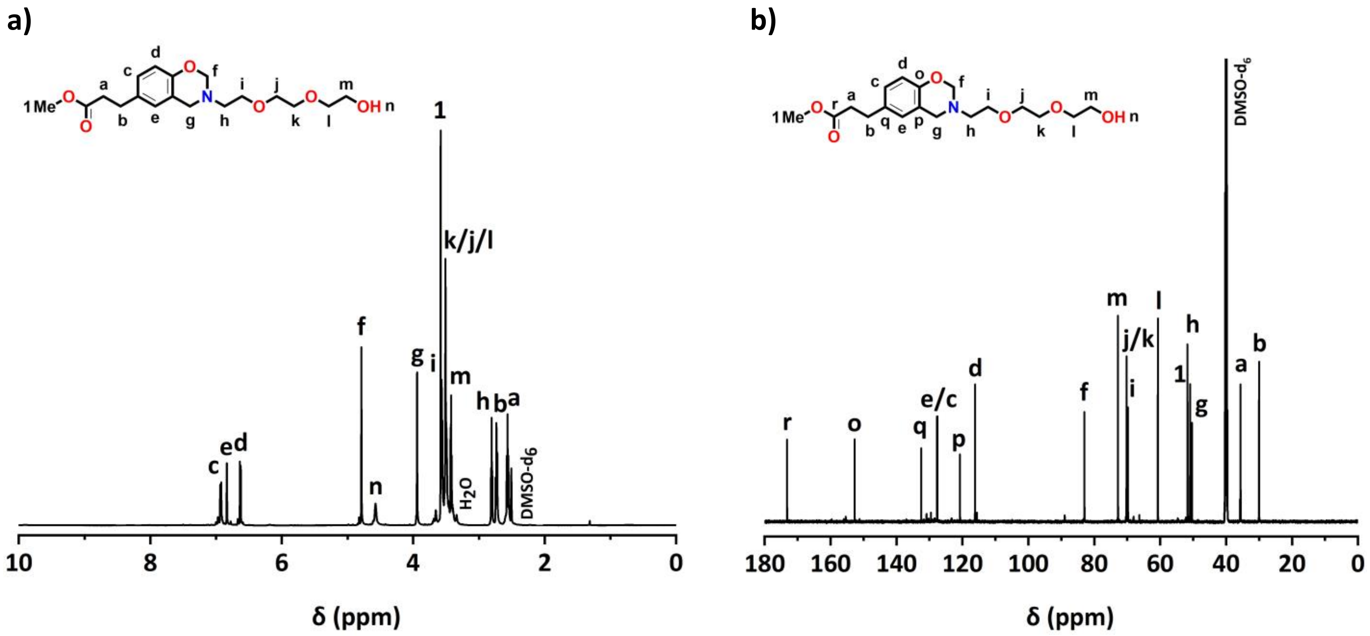

***Figure S6** a) ^{1}H and b) ^{13}C NMR spectra of Me-PA-tga.*

^{1}H NMR (DMSO-d_6, 600 MHz, 298°K): δ (ppm) = (assignment, multiplicity (coupling constant), [attribution], experimental integration, theoretical integration). δ= 2.56 (CH_2-CH_2*-C=O, t (J = 7.64 Hz), [a], exp 2.00H, th 2.00H); δ= 2.73 (CH_2-CH_2*-Ar, t (J= 7.64 Hz), [b], exp 2.02H, th 2.00H); δ= 2.81 (N-CH_2*-CH_2-O, t (J= 5.81 Hz), [h], exp 1.55H, th 2.00H); δ= 3.42 (O-CH_2-CH_2*-OH, t (J= 5.24 Hz), [m], exp 1.93H, th 2.00H); δ= 3.49 (O-CH_2*-CH_2-OH, m, [l], exp 1.94H, th 2.00H); δ= 3.51 (O-CH_2*-CH_2-O & O-CH_2-CH_2*-O, m, [j&k], exp 3.97H, th 4.00H); δ= 3.56-3.58 (N-CH_2-CH_2*-O & CH_3*-C=O, [i&1], exp 4.96H, th 5.00H); δ= 3.94 (Ar-CH_2*-N-, s, [g], exp 1.50H, th 2.00H); δ= 4.57 (CH_2-OH*, s, [n], exp 0.95H, th 1.00H); δ= 4.78 (O-CH_2*-N, s, [f], exp 1.50H, th 2.00H); δ= 6.62-6.93 (CH=CH*-C-O & C-CH*=C & C-CH*=CH, m, [d&e&c], exp 3.00H, th 3.00H).

^{13}C NMR (DMSO-d_6, 600 MHz, 298°K): δ (ppm) = 30.0 [b]; 35.6 [a]; 50.4 [g]; 50.9 [h]; 51.7 [1]; 60.7 [l]; 69.8 [i]; 70.1 [k]; 70.2 [j]; 72.8 [m]; 83.0 [f]; 116.2 [d]; 120.7 [p]; 127.6 [c]; 127.7 [e]; 132.5 [q]; 152.7 [o]; 173.2 [r].

- *Me-PA-fa*

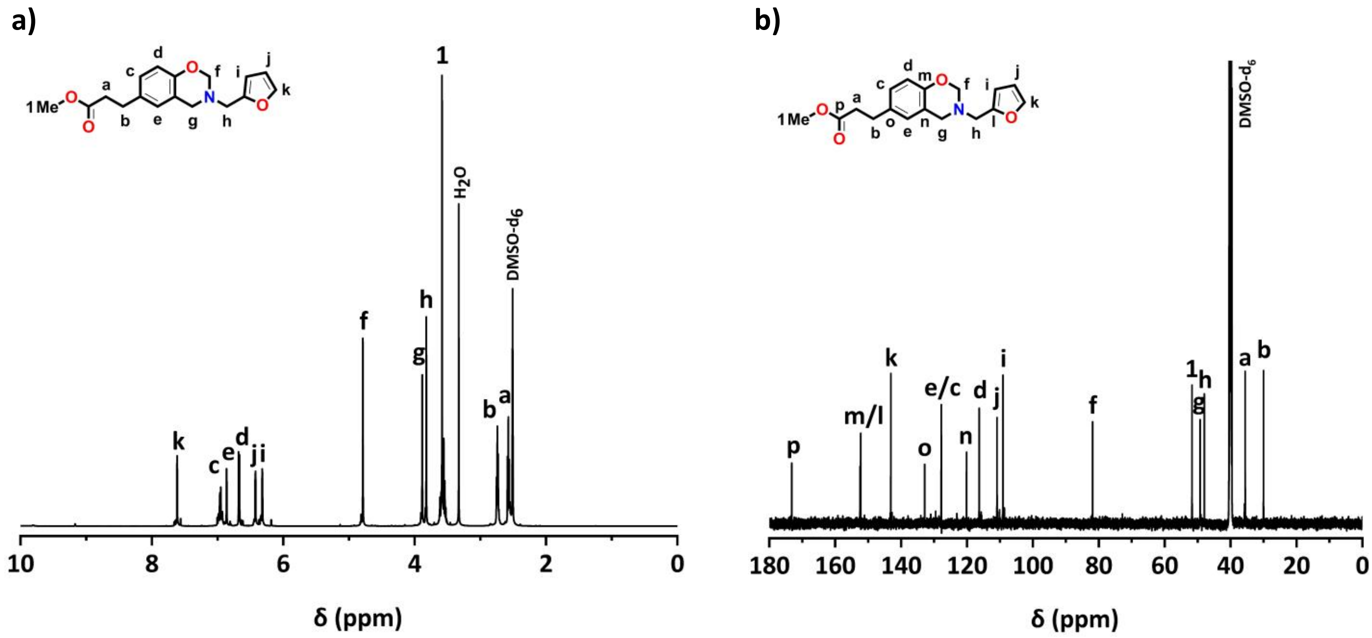

***Figure S7** a) ^{1}H and b) ^{13}C NMR spectra of Me-PA-fa.*

^{1}H NMR (DMSO-d_6, 600 MHz, 298°K): δ (ppm) = (assignment, multiplicity (coupling constant), [attribution], experimental integration, theoretical integration). δ= 2.57 (CH_2-CH_2*-C=O, t (J = 7.77 Hz), [a], exp 2.00H, th 2.00H); δ= 2.74 (CH_2-CH_2*-C, t (J = 7.61 Hz), [b], exp 2.02H, th 2.00H); δ= 3.58 (CH_3*-O-C=O, s, [1], exp 2.89H, th 3.00H); δ= 3.82 (*fa*-CH_2*-N, s, [h], exp 1.52H, th 2.00H); δ= 3.88 (Ar-CH_2*-N, s, [g], exp 1.52H, th 2.00H); δ= 4.79 (O-CH_2*-N, s, [f], exp 1.49H, th 2.00H); δ= 6.31-6.43 (*fa*-C-CH*=CH & *fa*-CH-CH*=CH, m, [i&j], exp 1.96H, th 2.00H); δ= 6.67-6.97 (CH=CH*-C-O & C-CH*=C & C-CH*=CH, m, [d&e&c], exp 3.07H, th 3.00H); δ= 7.62 (*fa*- CH=CH*-C-O, m, [k], exp 0.93H, th 1.00H).

^{13}C NMR (DMSO-d_6, 600 MHz, 298°K): δ (ppm) = 30.0 [b]; 35.6 [a]; 48.0 [h]; 49.2 [g]; 51.7 [1]; 81.9 [f]; 109.1 [i]; 110.9 [j]; 116.3 [d]; 120.1 [n]; 126.7 [c]; 126.8 [e]; 132.8 [o]; 143.1 [k]; 152.3 [l]; 152.5 [m]; 173.2 [p].

- *Me-DPA-mea*

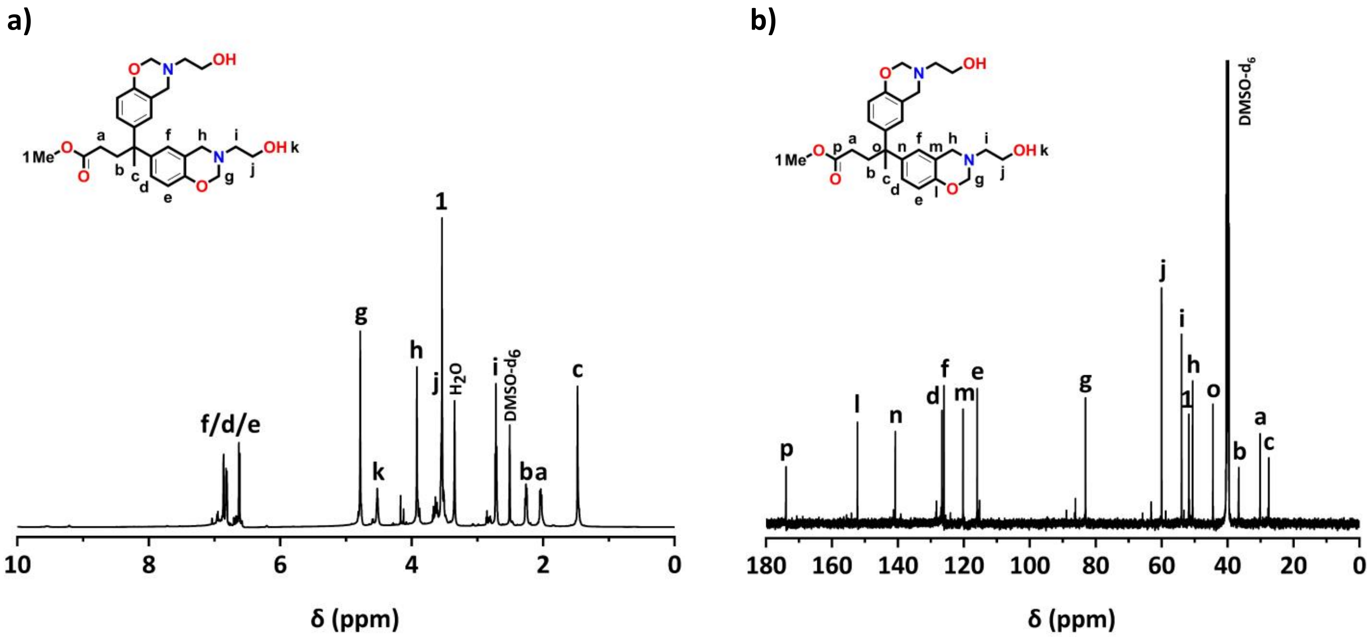

Figure S8 *a) ^{1}H and b) ^{13}C NMR spectra of Me-DPA-mea.*

^{1}H NMR (DMSO-d$_6$, 600 MHz, 298°K): δ (ppm) = (assignment, multiplicity (coupling constant), [attribution], experimental integration, theoretical integration). δ= 1.48 (CH_3*-C, s, [c], exp 3.00H, th 3.00H); δ= 2.03 (CH_2-CH_2*-C=O, t (J = 7.80 Hz), [a], exp 1.94H, th 2.00H); δ= 2.26 (CH_2-CH_2*-C, t (J = 7.95 Hz), [b], exp 1.95H, th 2.00H); δ= 2.72 (CH_2-CH_2*-N, t (J = 6.18 Hz), [i], exp 2.94H, th 4.00H); δ= 3.54 (CH_3*-O-C=O, m, [1&j], exp 6.35H, th 7.00H); δ= 3.92 (Ar-CH_2*-N, s, [h], exp 3.14H, th 4.00H); δ= 4.52 (CH_2-OH*, m, [k], exp 1.50H, th 2.00H); δ= 4.78 (O-CH_2*-N, s, [g], exp 3.21H, th 4.00H); δ= 6.62-6.86 (CH=CH*-C-O & C-CH*=CH & C-CH*=C, m, [e&d&f], exp 5.80H, th 6.00H).

^{13}C NMR (DMSO-d$_6$, 600 MHz, 298°K): δ (ppm) = 27.5 [c]; 30.1 [a]; 36.6 [b]; 44.5 [o]; 50.6 [h]; 51.8 [1]; 54.0 [i]; 60.0 [j]; 83.1 [g]; 115.9 [e]; 120.3 [m]; 126.0 [f]; 126.6 [d]; 140.8 [n]; 152.3 [l]; 173.9 [p].

➢ <u>*Me-DPA-fa*</u>

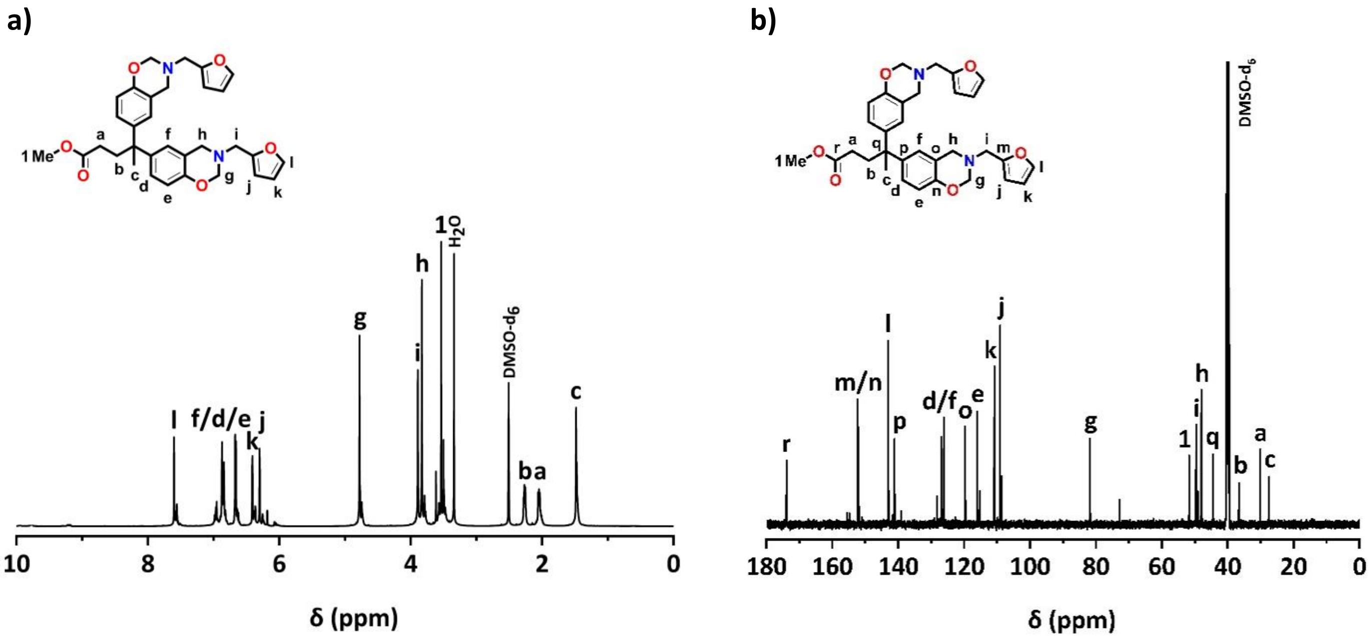

Figure S9 *a)* ^{1}*H and b)* ^{13}*C NMR spectra of Me-DPA-fa.*

^{1}H NMR (DMSO-d_6, 600 MHz, 298°K): δ (ppm) = (assignment, multiplicity (coupling constant), [attribution], experimental integration, theoretical integration). δ= 1.48 (CH_3*-C, s, [c], exp 3.00H, th 3.00H); δ= 2.05 (CH_2-CH_2*-C=O, t (J = 7.86 Hz), [a], exp 2.06H, th 2.00H); δ= 2.27 (CH_2-CH_2*-C, t (J = 7.85 Hz), [b], exp 2.00H, th 2.00H); δ= 3.54 (CH_3*-O-C=O, s, [1], exp 3.08H, th 3.00H); δ= 3.82-3.89 (Ar-CH_2*-N & *fa*-CH_2*-N, m, [h&i], exp 5.96H, th 8.00H); δ= 4.78 (O-CH_2*-N, s, [g], exp 2.96H, th 4.00H); δ= 6.29-6.41 (*fa*-C-CH*=CH & *fa*-CH-CH*=CH, m, [j&k], exp 3.43H, th 4.00H); δ= 6.66-6.97 (CH=CH*-C-O & C-CH*=CH & C-CH*=C, m, [e&d&f], exp 6.01H, th 6.00H); δ= 7.60 (*fa*- CH=CH*-C-O, m, [l], exp 1.66H, th 2.00H).

^{13}C NMR (DMSO-d_6, 600 MHz, 298°K): δ (ppm) = 27.5 [c]; 30.1 [a]; 36.6 [b]; 44.5 [q]; 48.0 [h]; 49.6 [i]; 51.7 [1]; 81.8 [g]; 109.1 [j]; 110.8 [k]; 116.0 [e]; 119.6 [o]; 126.2 [f]; 126.8 [d]; 141.1 [p]; 143.1 [l]; 152.0 [n]; 152.3 [m]; 173.9 [r].

- *Me-pHBA-mea*

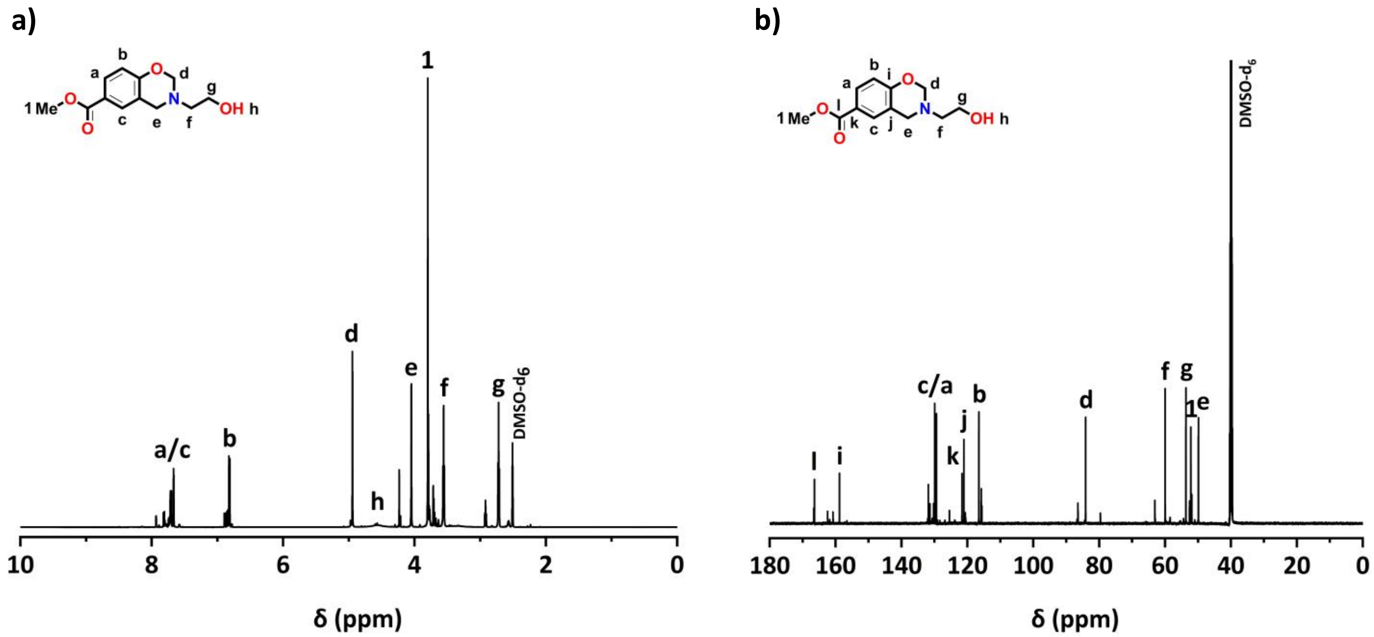

Figure S10 *a) ¹H and b) ¹³C NMR spectra of Me-pHBA-mea.*

¹H NMR (DMSO-d_6, 600 MHz, 298°K): δ= 2.72 (CH_2-CH_2*-N, t (J= 6.06 Hz), [g], exp 1.42H, th 2.00H); δ= 3.56 (CH_2-CH_2*-OH, t (J= 6.05 Hz), [f], exp 1.70H, th 2.00H); δ= 3.80 (CH_3*-C=O, s, [1], exp 3.00H, th 3.00H); δ= 4.05 (Ar-CH_2*-N-, s, [e], exp 1.41H, th 2.00H); δ= 4.57 (CH_2-OH*, s, [h], exp 0.71H, th 1.00H); δ= 4.94 (O-CH_2*-N, s, [d], exp 1.42H, th 2.00H); δ= 6.81 (CH=CH*-C-O, d (J= 8.52 Hz), [b], exp 1.01H, th 1.00H); δ= 7.66-7.72 (C-CH*=C & C-CH*=CH, m, [c&a], exp 2.06H, th 2.00H).

¹³C NMR (DMSO-d_6, 600 MHz, 298°K): δ (ppm) = 49.9 [e]; 52.2 [1]; 53.7 [g]; 60.0 [f]; 84.1 [d]; 116.5 [b]; 121.1 [j]; 121.6 [k]; 129.4 [c]; 129.9 [e]; 158.1 [i]; 166.4 [l].

- *Me-pHBA-fa*

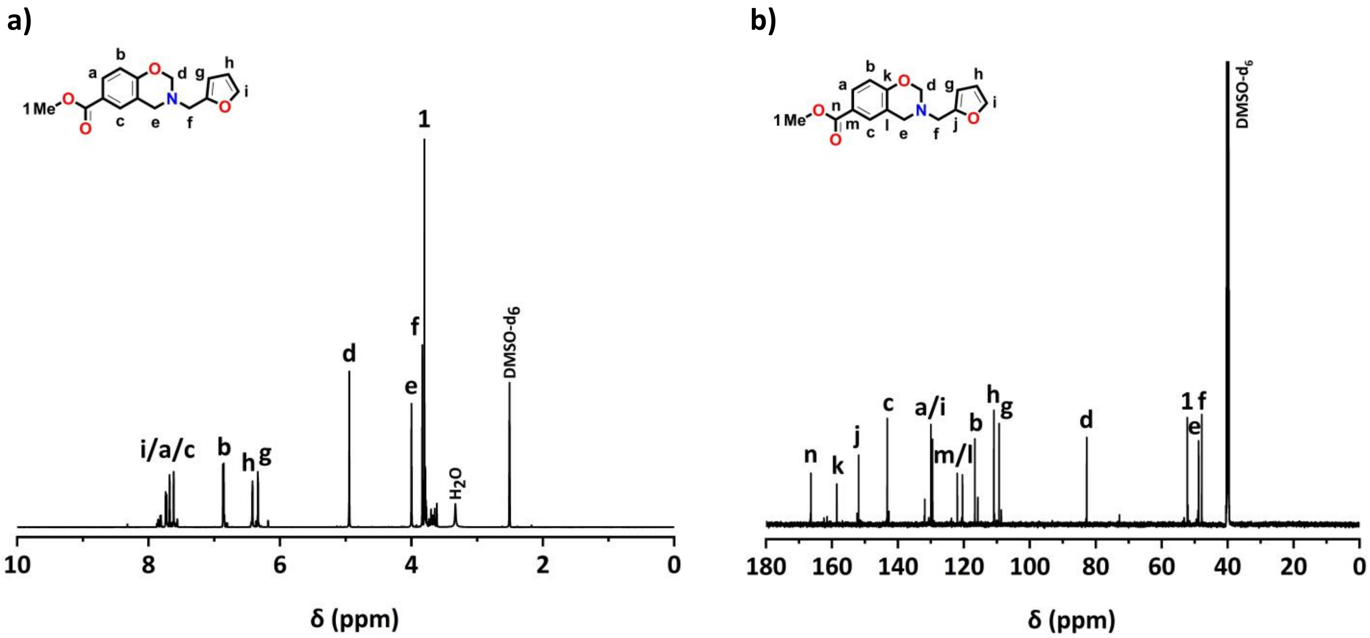

Figure S11 *a)* [1]*H and b)* [13]*C NMR spectra of Me-pHBA-fa.*

^{1}H NMR (DMSO-d_6, 600 MHz, 298°K): δ (ppm) = (assignment, multiplicity (coupling constant), [attribution], experimental integration, theoretical integration). δ= 3.80 (CH_3*-O-C=O, s, [1], exp 3.00H, th 3.00H); δ= 3.83 (*fa*-CH_2*-N, s, [f], exp 1.59H, th 2.00H); δ= 4.00 (Ar-CH_2*-N, s, [e], exp 1.64H, th 2.00H); δ= 4.95 (O-CH_2*-N, s, [d], exp 1.61H, th 2.00H); δ= 6.33 (*fa*-C-CH*=CH, d (J= 3.07 Hz), [g], exp 1.01H, th 1.00H); δ= 6.42 (*fa*-CH-CH*=CH, m, [h], exp 0.95H, th 1.00H); δ= 6.86 (CH=CH*-C-O, d (J= 8.42 Hz), [b], exp 1.02H, th 1.00H); δ= 7.62-7.68 (C-CH*=C & C-CH*=CH, m, [c&a], exp 1.83H, th 2.00H); δ= 7.73 (*fa*- CH=CH*-C-O, m, [i], exp 0.99H, th 1.00H).

^{13}C NMR (DMSO-d_6, 600 MHz, 298°K): δ (ppm) = 47.9 [f]; 49.0 [e]; 52.2 [1]; 82.8 [d]; 109.3 [g]; 110.9 [h]; 116.7 [b]; 120.4 [l]; 122.0 [m]; 129.5 [i]; 130.0 [a]; 143.2 [c]; 151.9 [j]; 158.5 [k]; 166.4 [n].

➢ *pPP-mea*

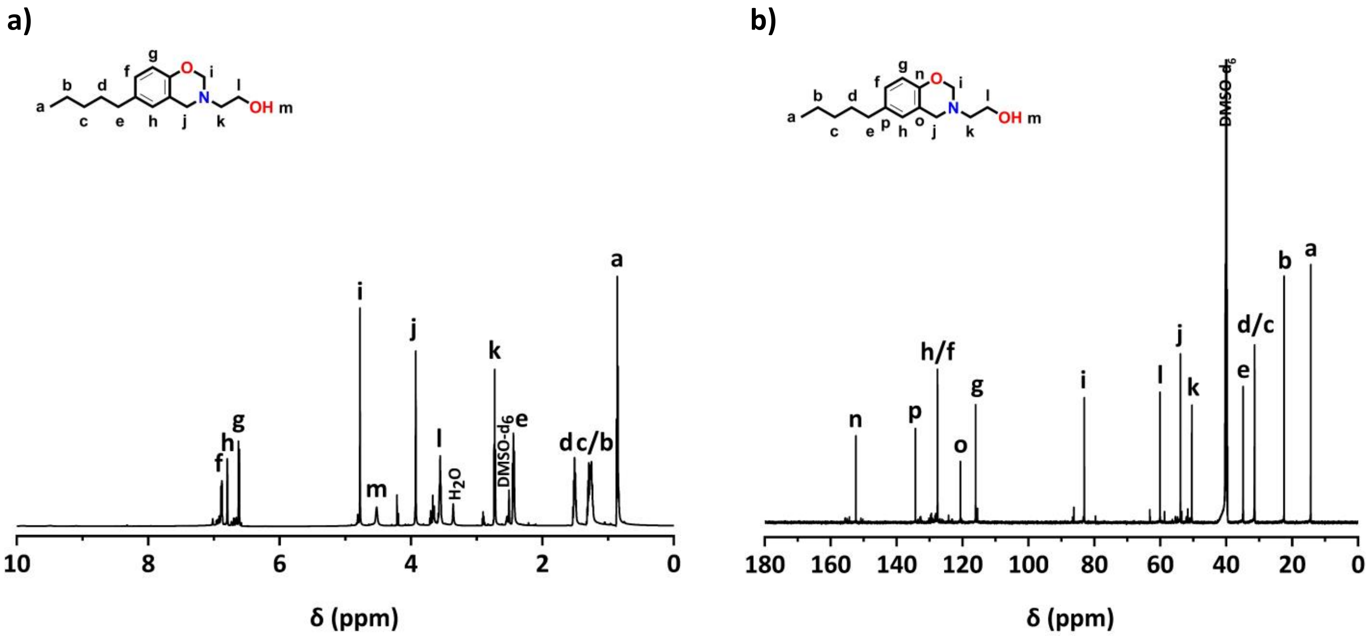

Figure S12 *a) ¹H and b) ¹³C NMR spectra of pPP-mea.*

^{1}H NMR (DMSO-d$_6$, 600 MHz, 298°K): δ (ppm) = (assignment, multiplicity (coupling constant), [attribution], experimental integration, theoretical integration). δ= 0.86 (CH_3*-CH_2, t (J = 7.14 Hz), [a], exp 3.00H, th 3.00H); δ= 1.27 (CH_3-CH_2*-CH_2 & CH_2-CH_2*-CH_2, m, [b&c], exp 4.02H, th 4.00H); δ= 1.51 (CH_2-CH_2*-CH_2, quint (J = 7.29 Hz), [d], exp 2.03H, th 2.00H); δ= 2.44 (CH_2-CH_2*-Ar, t (J = 7.66 Hz), [e], exp 1.96H, th 2.00H); δ= 2.73 (CH_2-CH_2*-N, t (J = 6.13 Hz, [k], exp 1.49H, th 2.00H); δ= 3.56 (CH_2-CH_2*-OH, t (J= 5.70 Hz), [l], exp 1.95H, th 2.00H); δ= 3.93 (Ar-CH_2*-N-, s, [j], exp 1.52H, th 2.00H); δ= 4.52 (CH_2-OH*, s, [m], exp 0.91H, th 1.00H); δ= 4.78 (O-CH_2*-N, s, [i], exp 1.50H, th 2.00H); δ= 6.62-6.92 (CH=CH*-C-O & C-CH*=C & C-CH*=CH, m, [g&h&f], exp 2.86H, th 3.00H).

^{13}C NMR (DMSO-d$_6$, 600 MHz, 298°K): δ (ppm) = 14.4 [a]; 22.4 [b]; 31.3 [c]; 31.4 [d]; 34.9 [e]; 50.4 [k]; 53.9 [j]; 60.1 [l]; 83.1 [i]; 116.0 [g]; 120.6 [o]; 127.5 [f]; 127.6 [h]; 134.3 [p]; 152.3 [n].

➢ *pPP-fa*

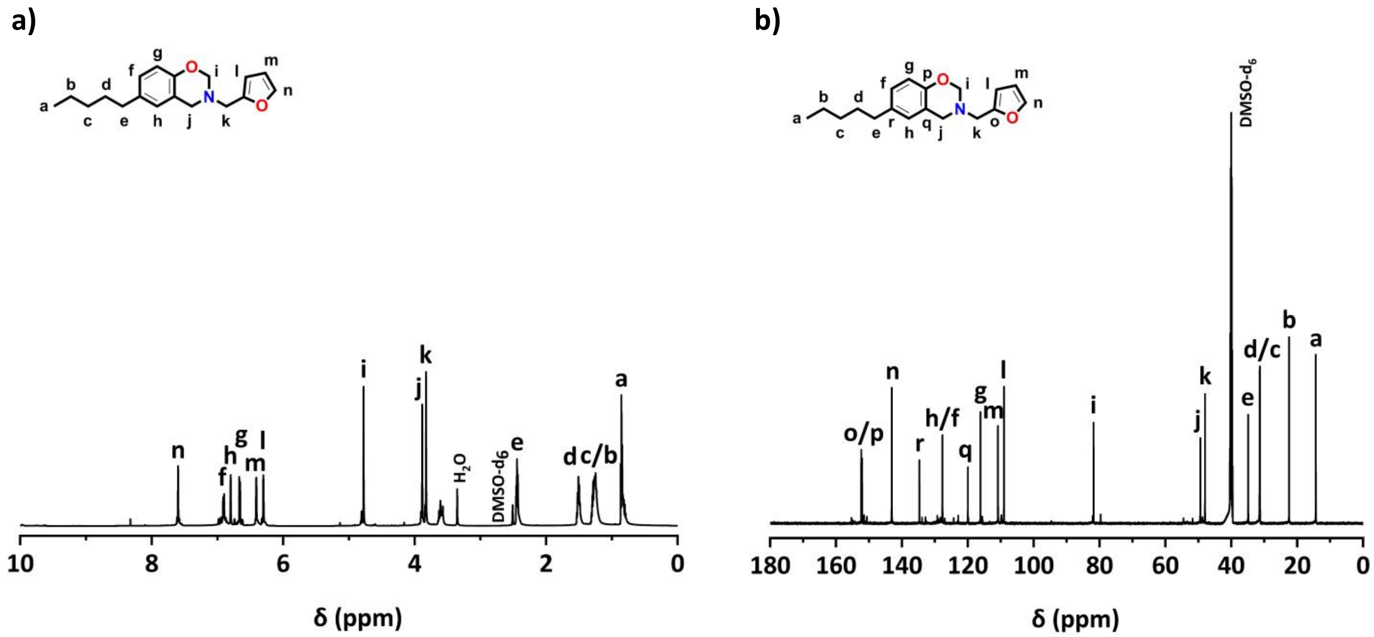

Figure S13 *a)* ^{1}*H and b)* ^{13}*C NMR spectra of pPP-fa.*

^{1}H NMR (DMSO-d$_6$, 600 MHz, 298°K): δ (ppm) = (assignment, multiplicity (coupling constant), [attribution], experimental integration, theoretical integration). δ= 0.85 (CH_3*-CH_2, t (J = 7.07 Hz), [a], exp 3.00H, th 3.00H); δ= 1.27 (CH_3-CH_2*-CH_2 & CH_2-CH_2*-CH_2, m, [b&c], exp 4.05H, th 4.00H); δ= 1.50 (CH_2-CH_2*-CH_2, quint (J = 7.16 Hz), [d], exp 2.02H, th 2.00H); δ= 2.43 (CH_2-CH_2*-Ar, t (J = 7.73 Hz), [e], exp 1.99H, th 2.00H); δ= 3.82 (*fa*-CH_2*-N, s, [11], exp 1.53H, th 2.00H); δ= 3.88 (Ar-CH_2*-N-, s, [9], exp 1.47H, th 2.00H); δ= 4.77 (O-CH_2*-N, s, [i], exp 1.50H, th 2.00H); δ= 6.29 (*fa*-C-CH*=CH, d (J= 3.22 Hz), [l], exp 0.92H, th 1.00H); δ= 6.40 (*fa*-CH-CH*=CH, m, [m], exp 0.97H, th 1.00H); δ= 6.65-6.90 (CH=CH*-C-O & C-CH*=C & C-CH*=CH, m, [g&h&f], exp 3.05H, th 3.00H); δ= 7.59 (*fa*- CH=CH*-C-O, m, [n], exp 0.92H, th 1.00H).

^{13}C NMR (DMSO-d$_6$, 600 MHz, 298°K): δ (ppm) = 14.3 [a]; 22.4 [b]; 31.3 [c]; 31.4 [d]; 34.9 [e]; 48.0 [k]; 49.3 [j]; 81.8 [i]; 109.0 [l]; 110.8 [m]; 116.1 [g]; 120.0 [q]; 127.6 [f]; 127.7 [h]; 134.7 [r]; 143.0 [n]; 152.1 [p]; 152.3 [o].

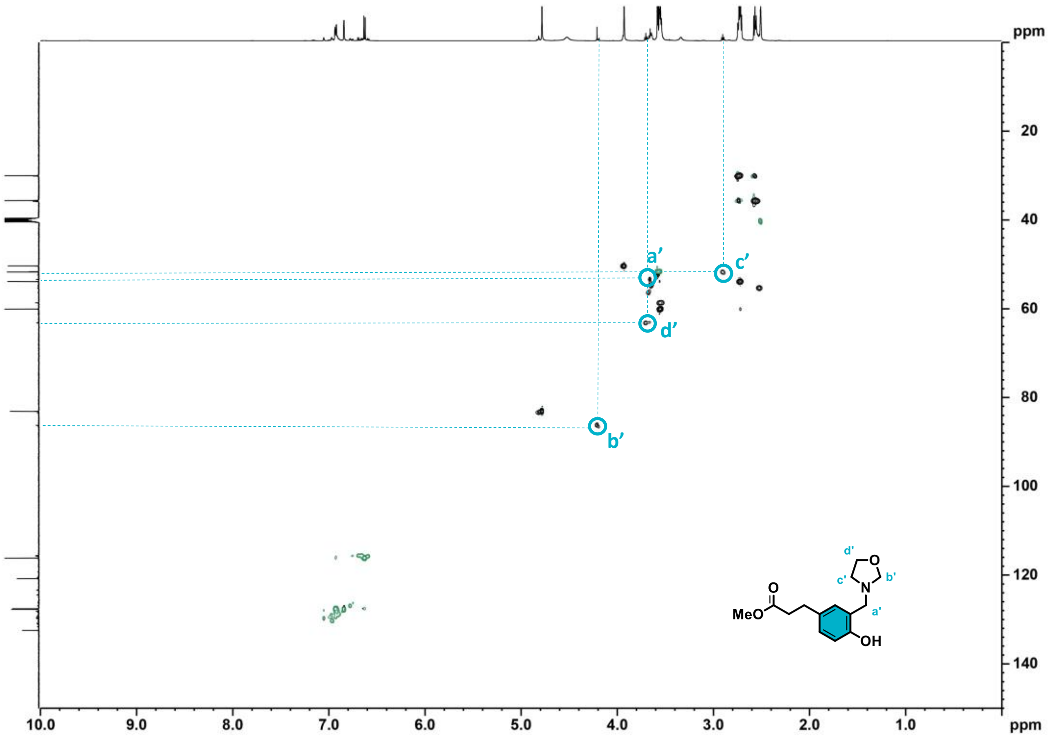

Figure S14 *HSQC spectrum of Me-PA-mea.*

Crosspeak $^{1}H/^{13}C$ (ppm)= 2.90/ 52.0 [c’], 3.66/ 53.4 [a’], 3.70/ 63.1 [d’], 4.21/ 86.3 [b’].

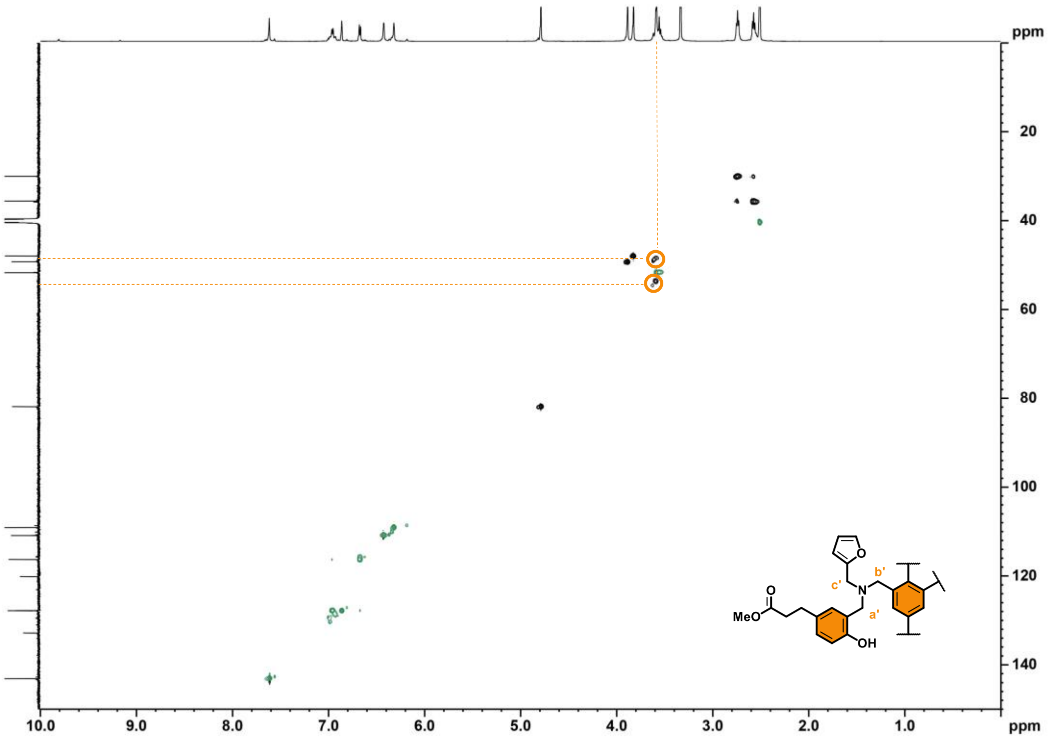

Figure S15 *HSQC spectrum of Me-PA-fa.*

Crosspeak $^{1}H/^{13}C$ (ppm)= 3.60/ 48.5 [a’], 3.60/ 53.7 [b’], 3.62/ 54.6 [c’].

Table S2 *Ratio of closed oxazine ring determined by ^{1}H NMR experiment.*

Molecule	[a] Ar–CH_2*–N (%)	[a] N–CH_2*–O (%)
Me-PA-mea	75	75
Me-PA-dga	80	81
Me-PA-tga	75	75
Me-PA-fa	76	75
Me-DPA-mea	79	80
Me-DPA-fa	75	74
Me-*p*HBA-mea	71	71
Me-*p*HBA-fa	82	81
*p*PP-mea	76	75
*p*PP-fa	74	75

[a] determined by experimental integration of the corresponding peaks in ^{1}H NMR

2. Monitoring of benzoxazine ring-opening polymerization

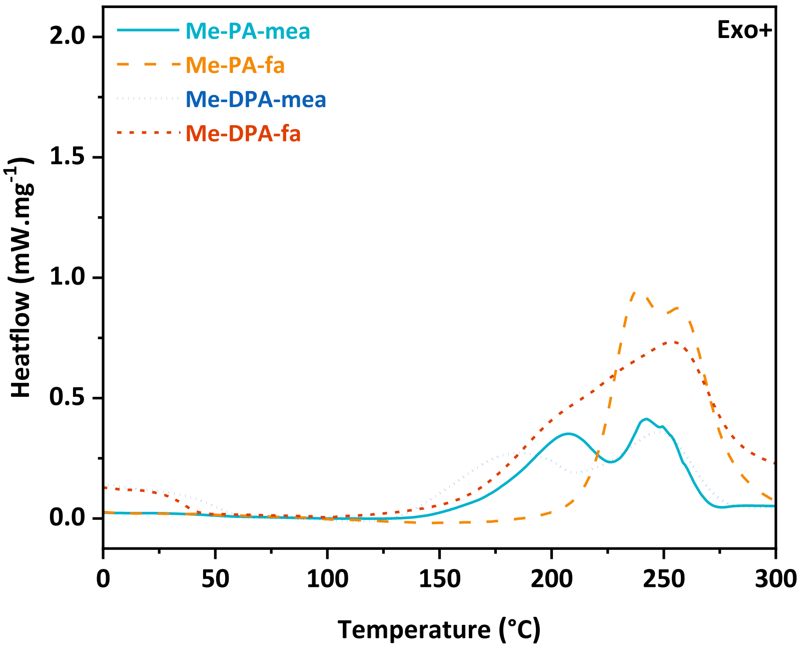

Figure S16 *DSC thermograms of Me-PA-mea, Me-PA-fa, Me-DPA-mea, and Me-DPA-fa (10°C.min^{-1}).*

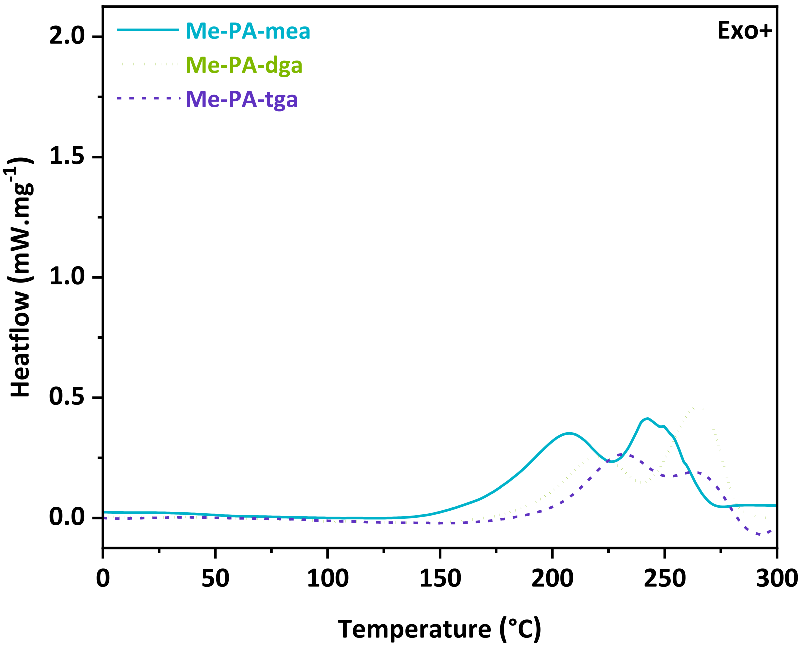

Figure S17 *DSC thermograms of Me-PA-mea, Me-PA-dga, and Me-PA-tga (10°C.min^{-1}).*

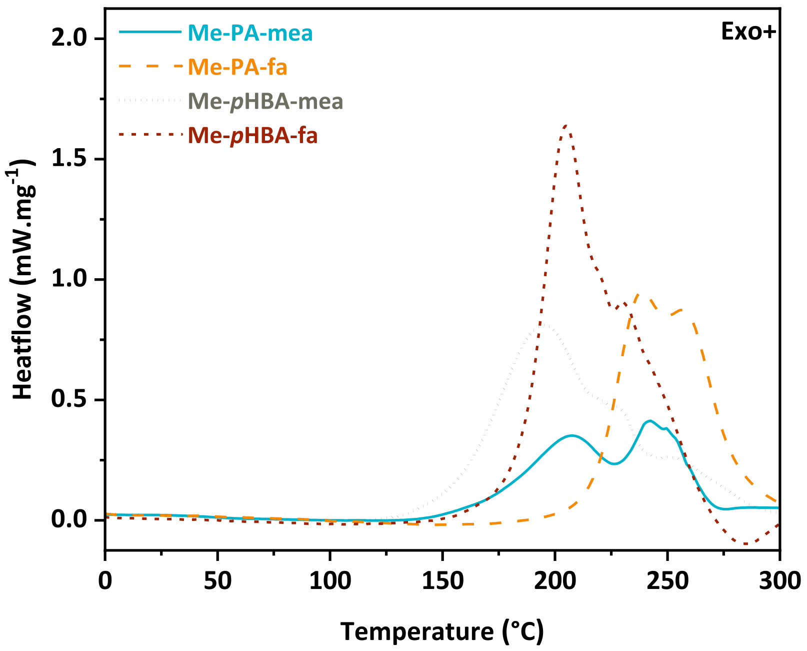

Figure S18 *DSC thermograms of Me-PA-mea, Me-PA-fa, Me-pHBA-mea, and Me-pHBA-fa (10°C.min^{-1}).*

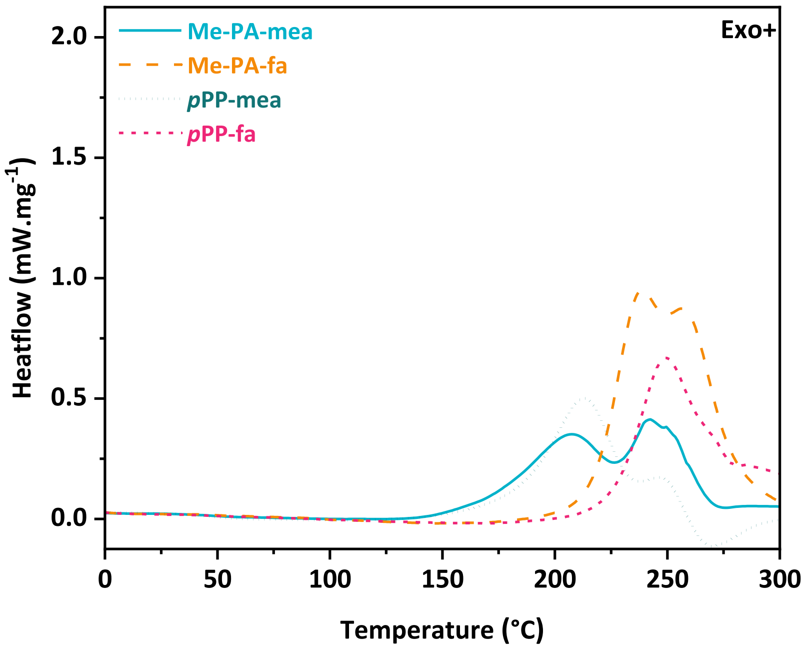

Figure S19 *DSC thermograms of Me-PA-mea, Me-PA-dga, pPP-mea, and pPP-fa (10°C.min^{-1}).*

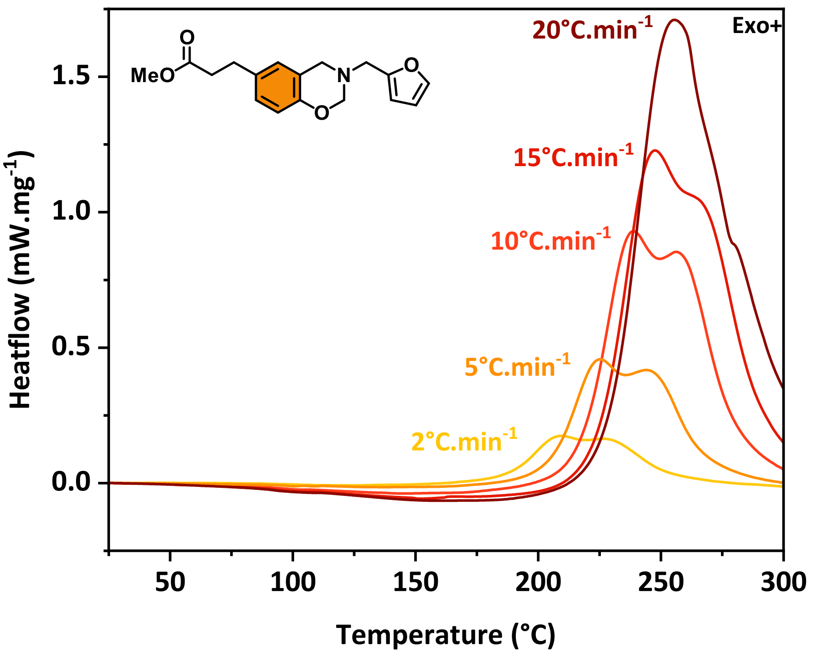

Figure S20 *DSC thermograms of Me-PA-fa with the heating rate ranging from 2 to 20°C.min^{-1}.*

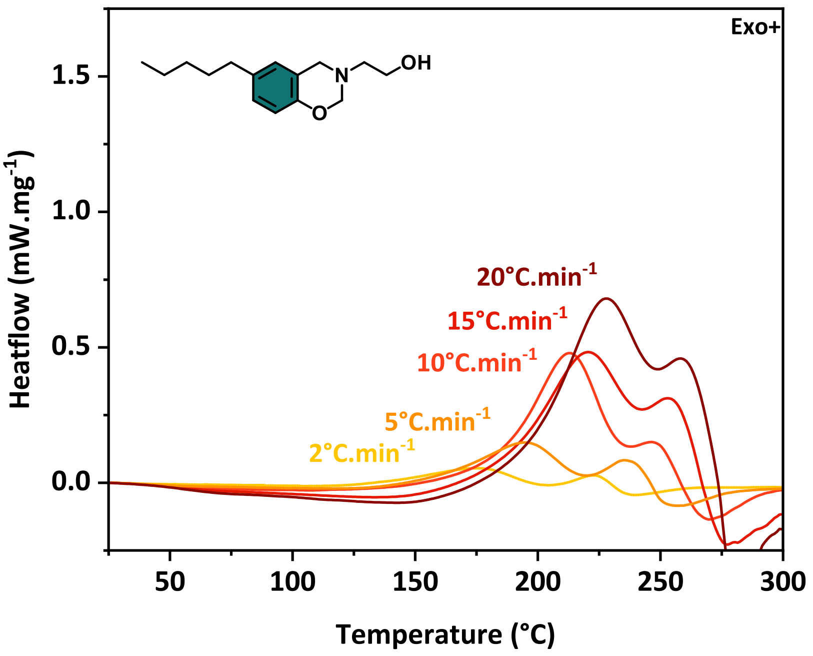

Figure S21 *DSC thermograms of pPP-mea with the heating rate ranging from 2 to 20°C.min^{-1}.*

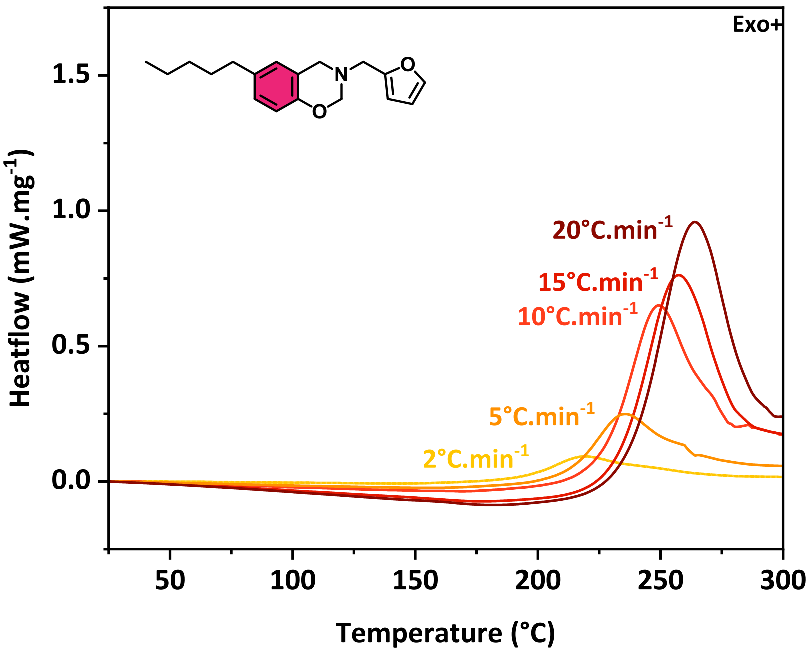

Figure S22 *DSC thermograms of pPP-fa with the heating rate ranging from 2 to 20°C.min^{-1}.*

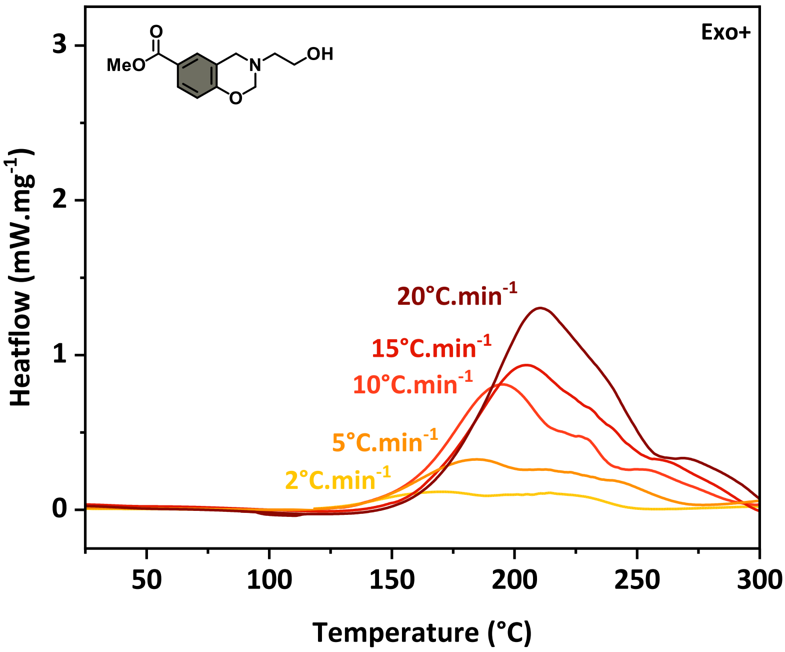

Figure S23 *DSC thermograms of Me-pHBA-mea with the heating rate ranging from 2 to 20°C.min^{-1}.*

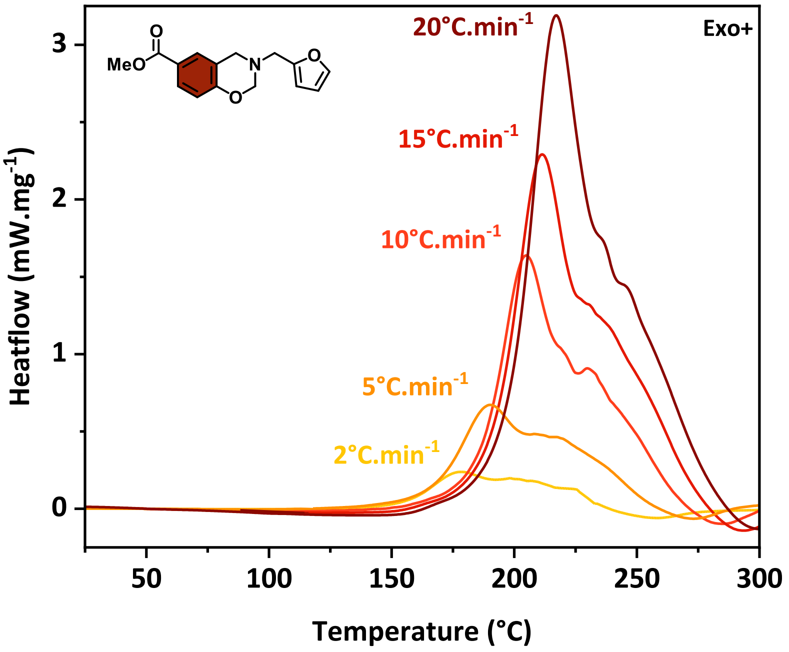

Figure S24 *DSC thermograms of Me-pHBA-fa with the heating rate ranging from 2 to 20°C.min^{-1}.*

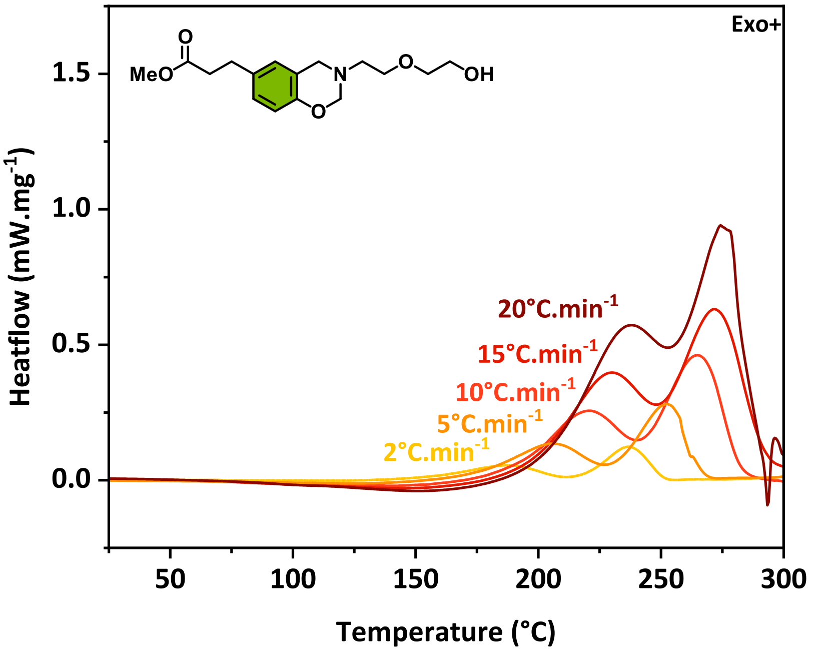

Figure S25 *DSC thermograms of Me-PA-dga with the heating rate ranging from 2 to 20°C.min^{-1}.*

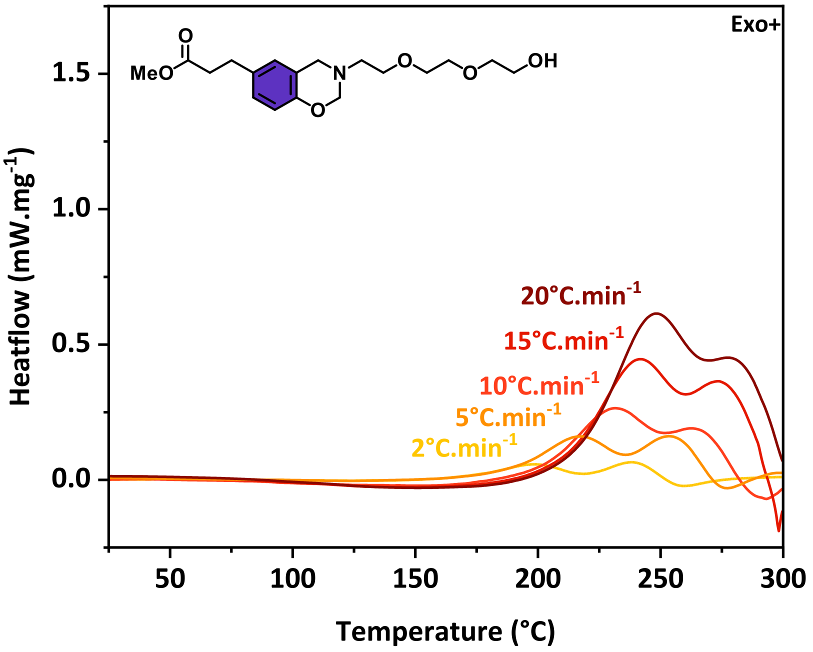

Figure S26 *DSC thermograms of Me-PA-tga with the heating rate ranging from 2 to 20°C.min^{-1}.*

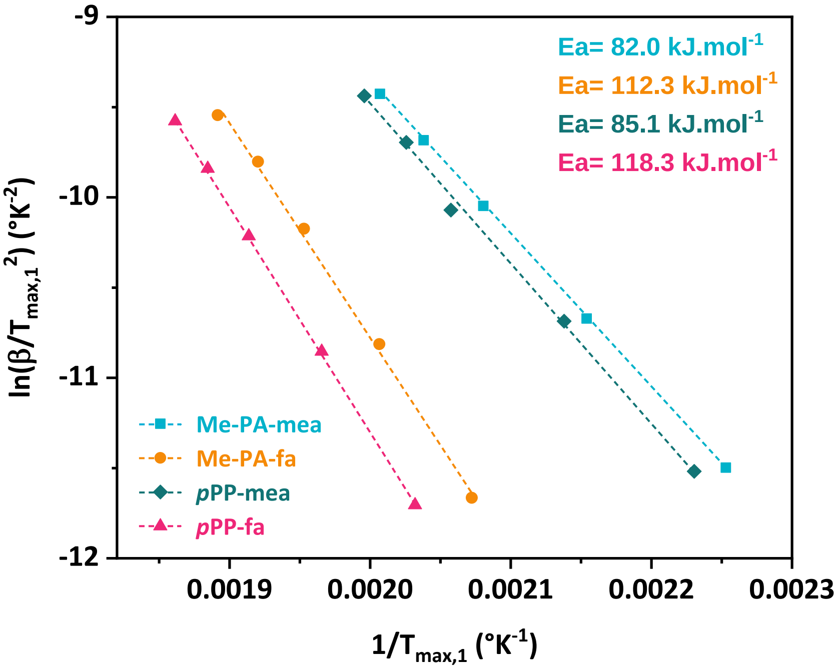

Figure S27 *Determination of the activation energy of benzoxazine ring-opening polymerization using the Kissinger equation:* $ln(\frac{\beta}{Tp^2})$ *against* $\frac{1}{Tp}$.

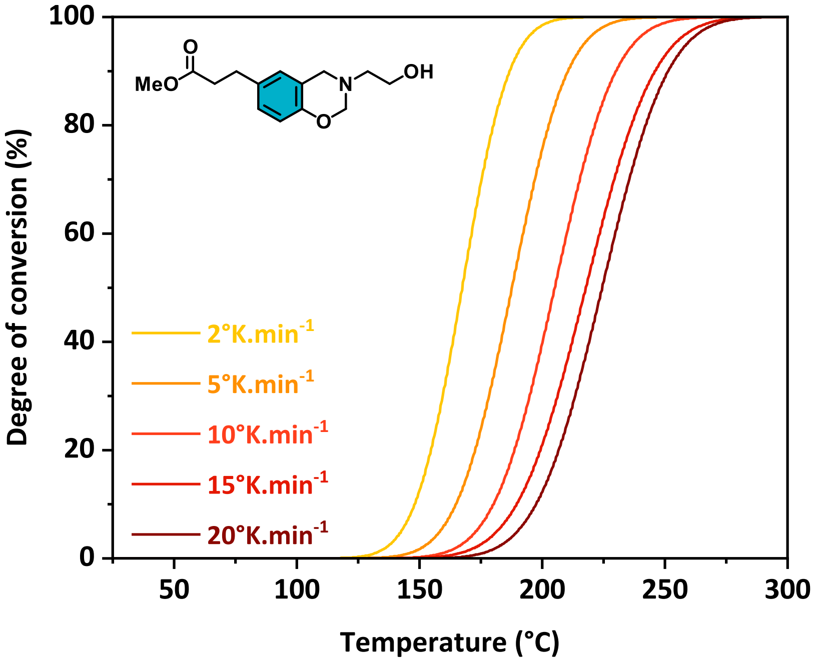

Figure S28 *Degree of conversion of Me-PA-mea at different heating rates (2, 5, 10, 15, and 20°C.min*$^{-1}$*) determined from deconvoluted exothermic peak.*

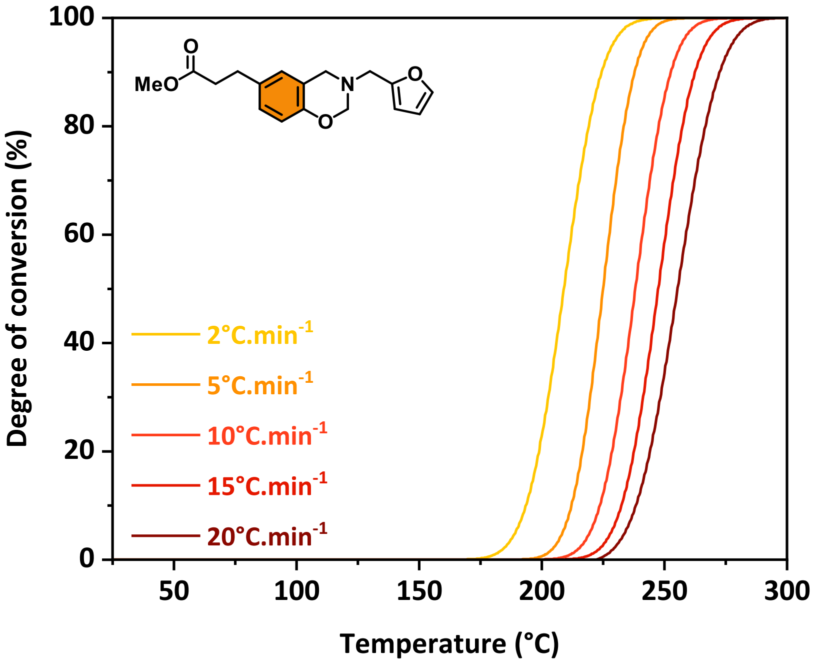

Figure S29 *Degree of conversion of Me-PA-fa at different heating rates (2, 5, 10, 15, and 20°C min*$^{-1}$*) determined from deconvoluted exothermic peak.*

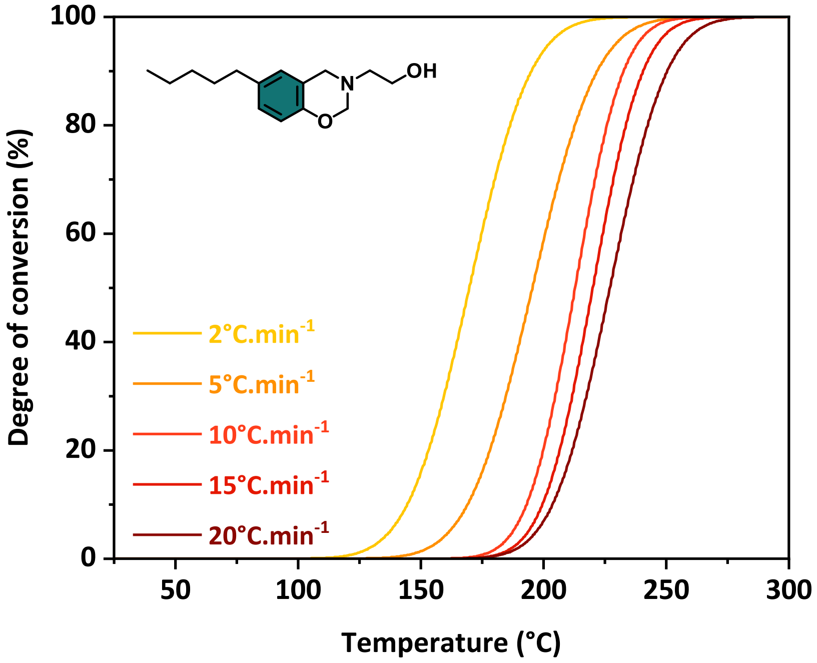

Figure S30 *Degree of conversion of pPP-mea at different heating rates (2, 5, 10, 15, and 20°C min^{-1}) determined from deconvoluted exothermic peak.*

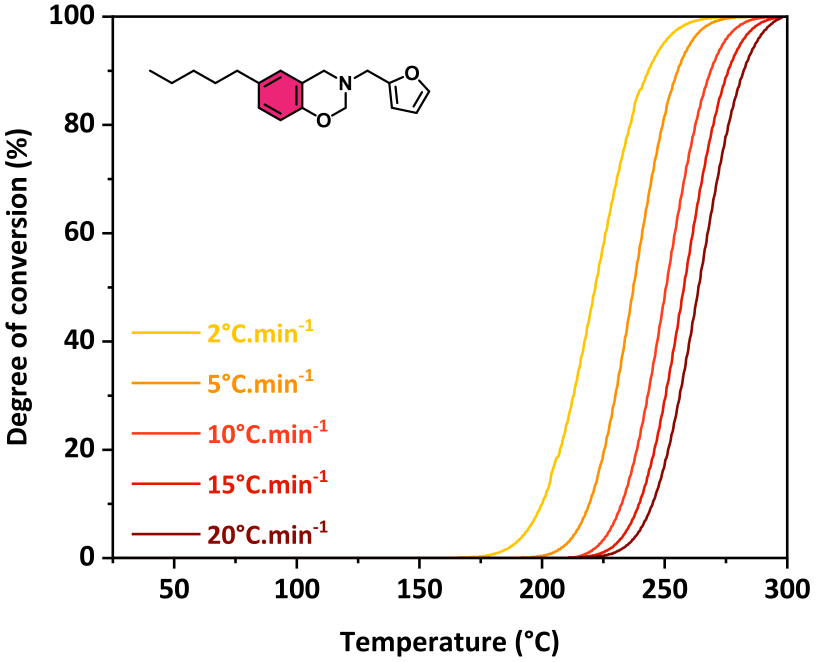

Figure S31 *Degree of conversion of pPP-fa at different heating rates (2, 5, 10, 15, and 20°C min^{-1}) determined from deconvoluted exothermic peak.*

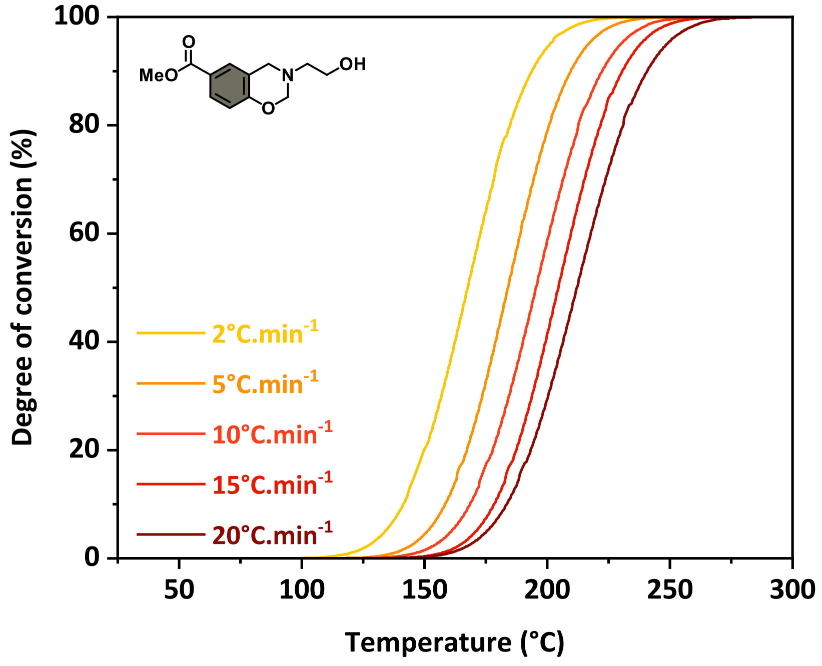

Figure S32 *Degree of conversion of Me-pHBA-mea at different heating rates (2, 5, 10, 15, and 20°C.min^{-1}) determined from deconvoluted exothermic peak.*

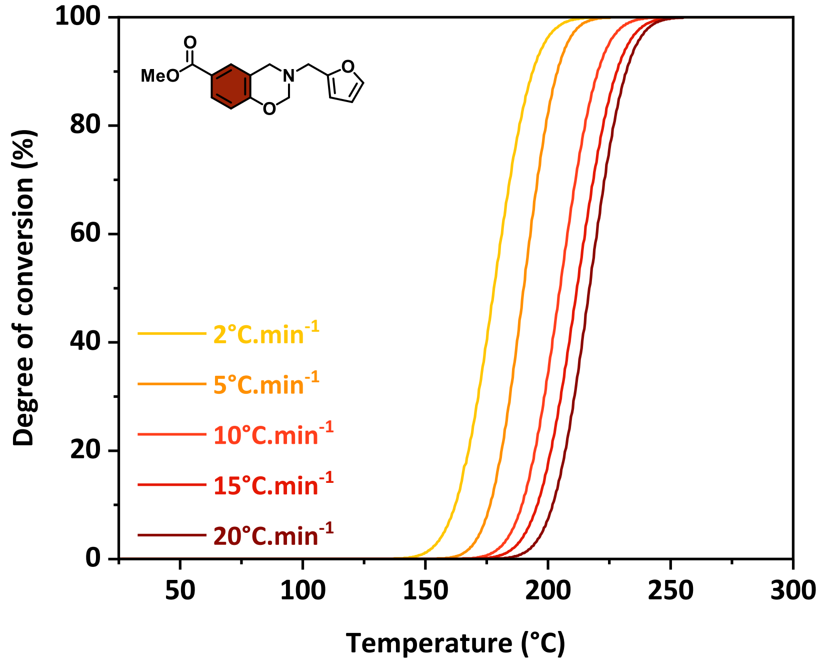

Figure S33 *Degree of conversion of Me-pHBA-fa at different heating rates (2, 5, 10, 15, and 20°C min^{-1}) determined from deconvoluted exothermic peak.*

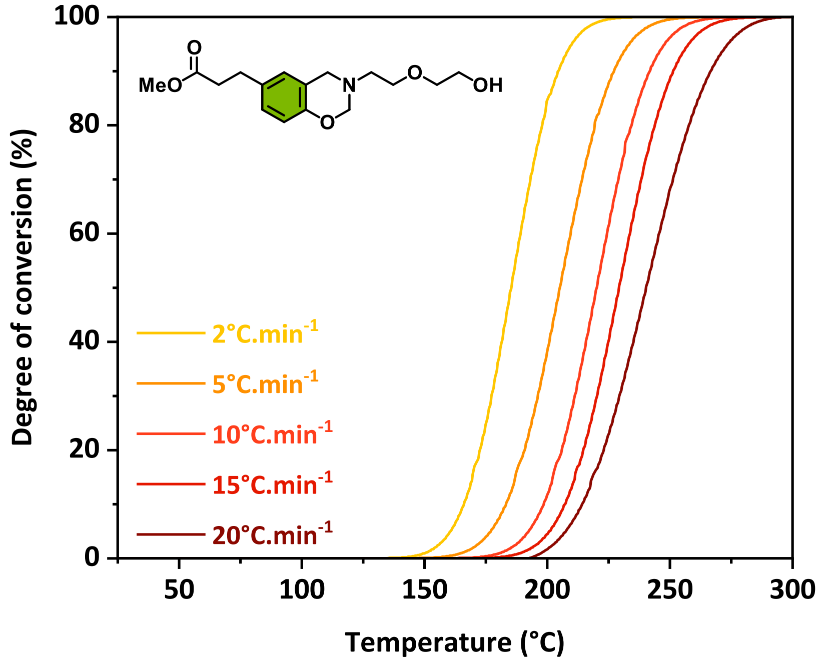

Figure S34 *Degree of conversion of Me-PA-dga at different heating rates (2, 5, 10, 15, and 20°C.min^{-1}) determined from deconvoluted exothermic peak.*

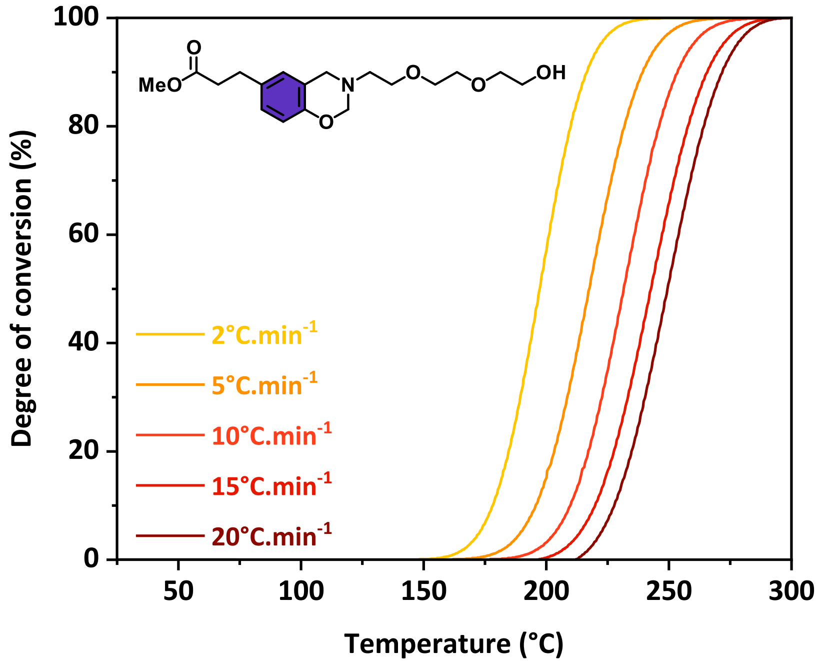

Figure S35 *Degree of conversion of Me-PA-tga at different heating rates (2, 5, 10, 15, and 20°C min^{-1}) determined from deconvoluted exothermic peak.*

Table S3 *Onset temperature of ring-opening reaction determined by DSC experiment and activation energy of benzoxazine ring-opening polymerization determined by Kissinger model, FWO equation, and numerical optimization of isoconversional Friedman method (performed on deconvoluted peaks).*

Molecule	[a] T_{onset} (°C)	Kissinger		Flynn-Wall-Ozawa		Numerical Optimization (Friedman)	
		Ea (kJ.mol^{-1})	R^2	Ea (kJ.mol^{-1})	R^2	Ea (kJ.mol^{-1})	R^2
Me-PA-mea	131.5	70.1	1	82.0	1	74.4 (± 0.7)	0.9937
Me-PA-dga	146.3	82.2	0.9944	94.9	0.9995	87.0 (± 2.5)	0.9861
Me-PA-tga	182.8	86.5	0.9995	99.7	0.9995	98.4 (± 1.1)	0.9899
Me-PA-fa	181.4	98.4	0.9972	112.3	0.9977	117.6 (± 2.6)	0.9862
Me-DPA-mea	122.8	[b] n.c.	[b] n.c.	[b] n.c.	[b] n.c.	[b] n.c.	[b] n.c.
Me-DPA-fa	162.2	[b] n.c.	[b] n.c.	[b] n.c..	[b] n.c.	[b] n.c..	[b] n.c.
Me-*p*HBA-mea	114.4	93.6	0.9956	106.6	0.9963	102.4 (± 4.8)	0.9935
Me-*p*HBA-fa	167.9	98.9	0.9934	112.3	0.9944	120.1 (± 3.3)	0.9888
*p*PP-mea	135.5	73.0	0.9977	85.1	0.9981	73.7 (± 4.9)	0.9877
*p*PP-fa	201.8	103.9	0.9998	118.3	0.9998	113.8 (± 5.2)	0.9874

[a] heating rate of 10°C.min^{-1}, [b] not fitting with Kissinger or Flynn Wall-Ozawa equations, and numerical optimization of Friedman method.

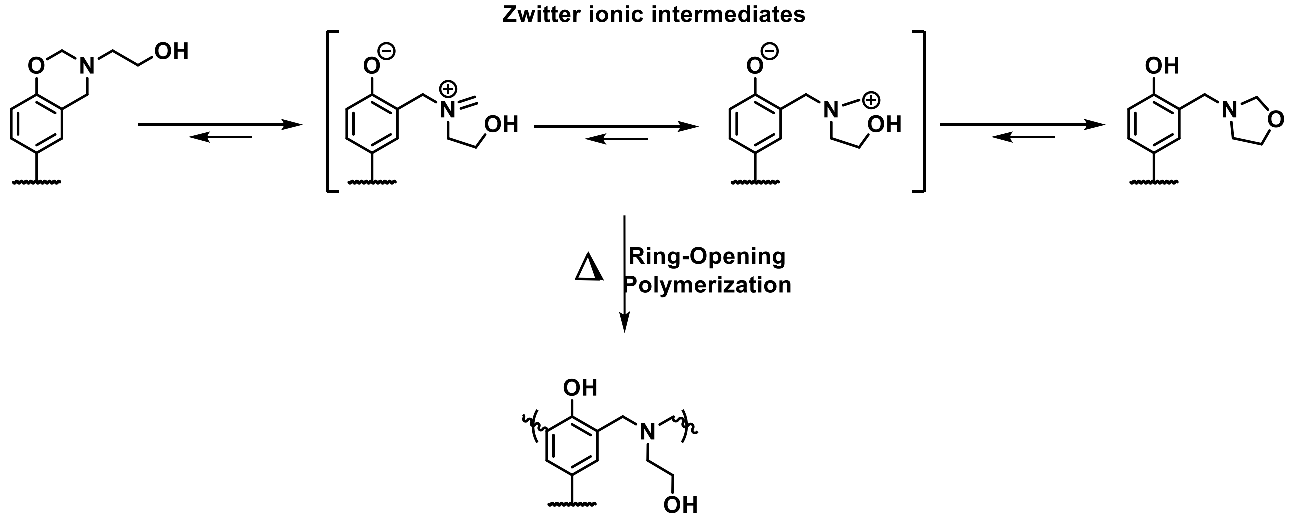

Scheme S2 *NGP-assisted ring-opening polymerization of benzoxazine containing β-amino-alcohol.*

3. Monitoring of transesterification reactions

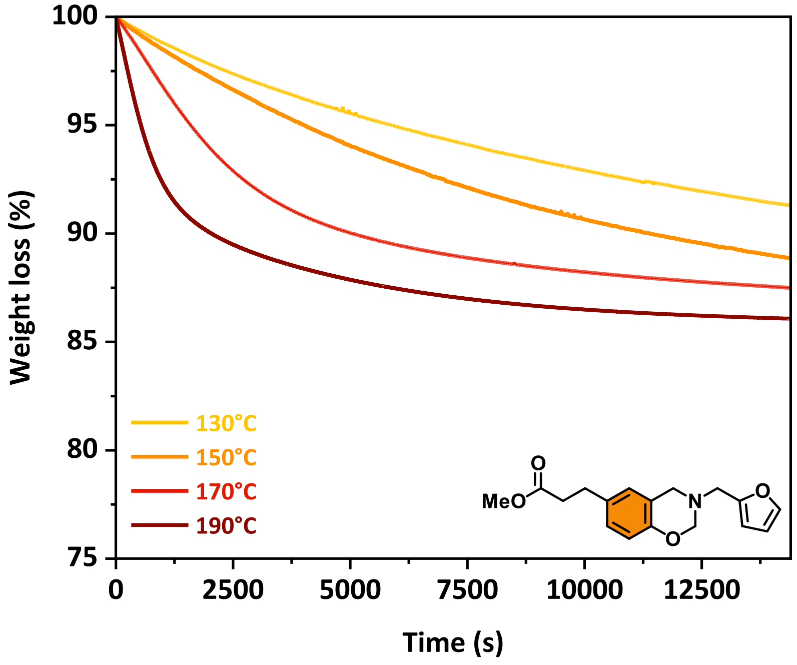

Figure S36 *Isothermal TGA curves of Me-PA-fa.*

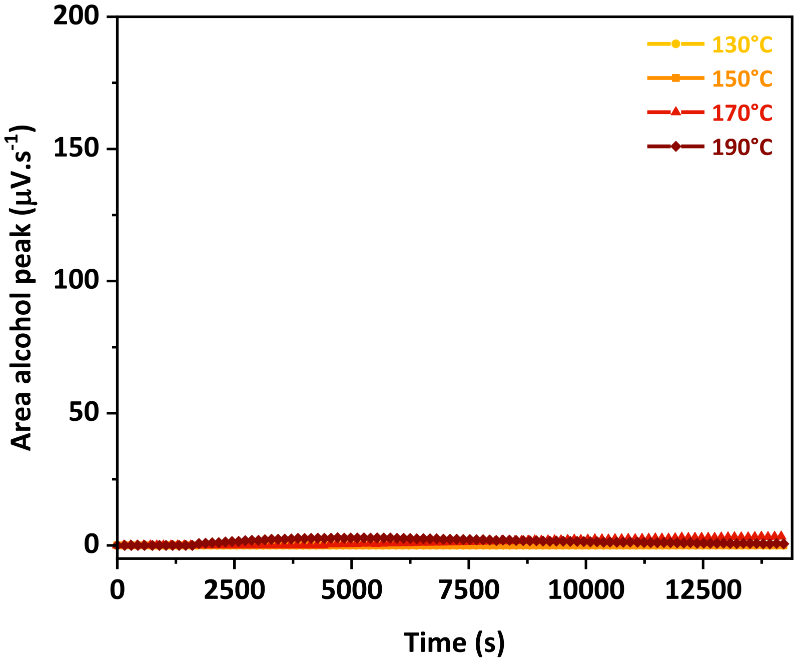

Figure S37 *Area methanol peak determined on the µGC spectrum of Me-PA-fa.*

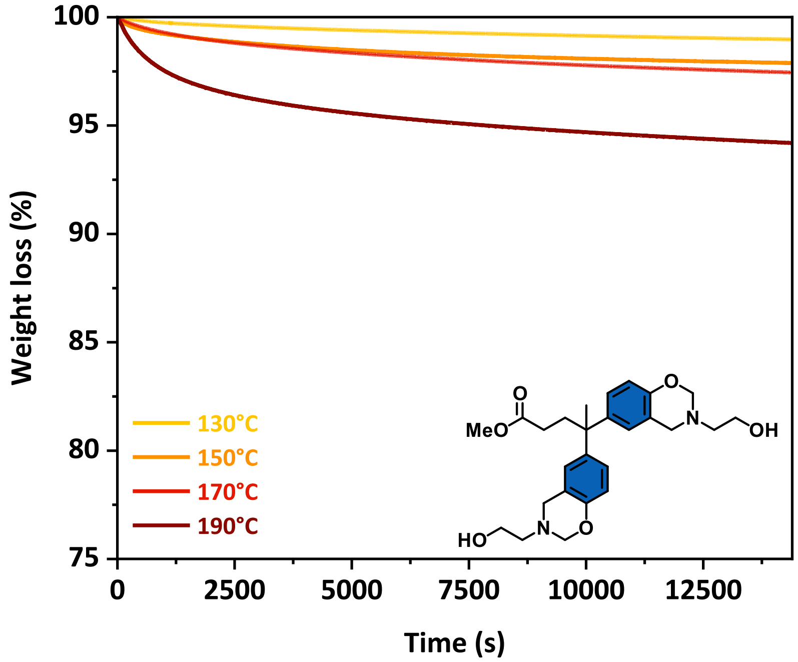

Figure S38 *Isothermal TGA curves of Me-DPA-mea.*

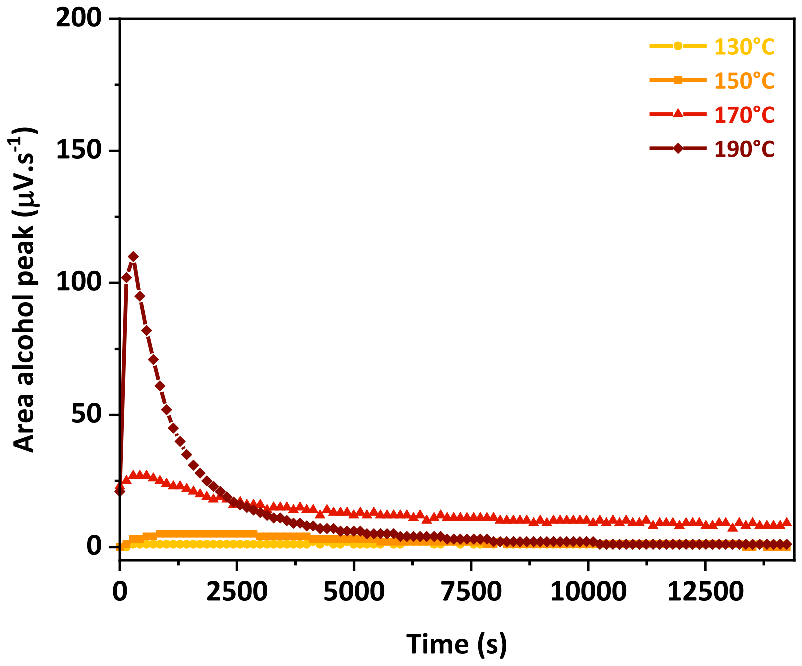

Figure S39 *Area methanol peak determined on the µGC spectrum of Me-DPA-mea.*

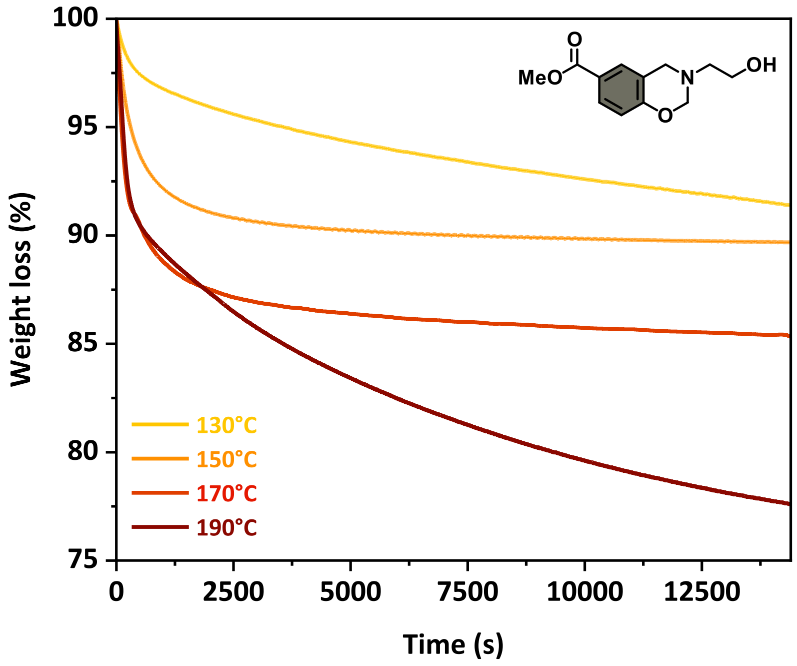

Figure S40 *Isothermal TGA curves of Me-pHBA-mea.*

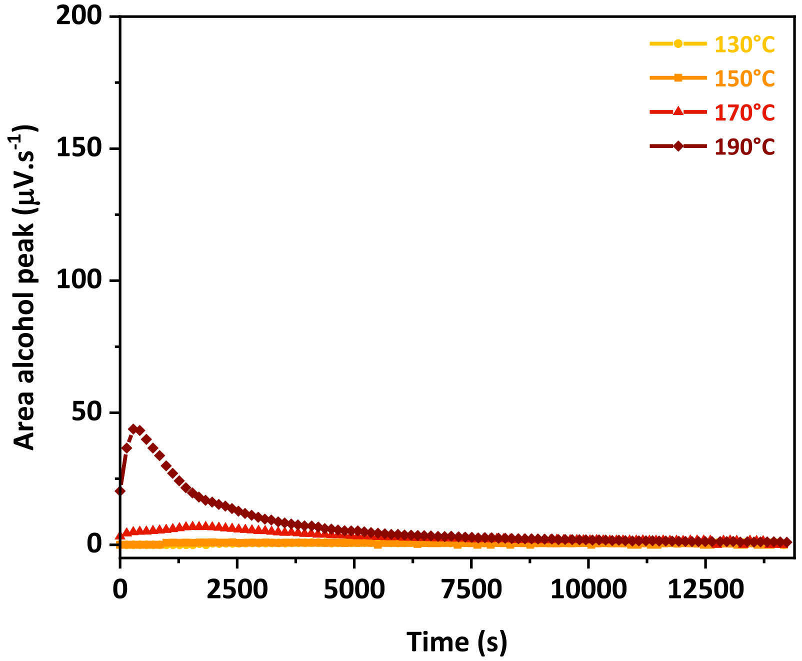

Figure S41 *Area methanol peak determined on the µGC spectrum of Me-pHBA-mea.*

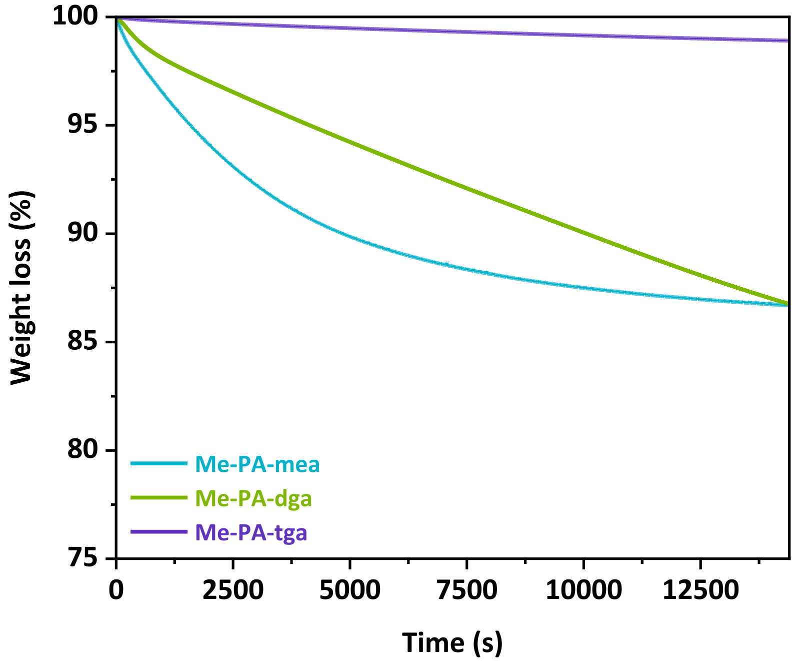

Figure S42 *Isothermal TGA curves of Me-PA-mea, Me-PA-dga, and Me-PA-dga (190°C).*

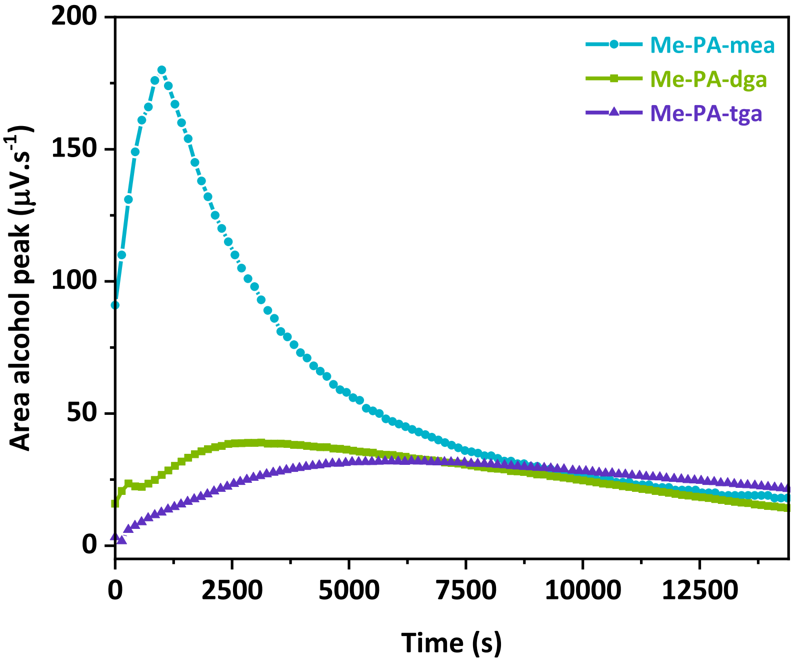

Figure S43 *Area methanol peak at 190°C determined on the µGC spectrum of Me-PA-mea, Me-PA-dga, and Me-PA-tga.*

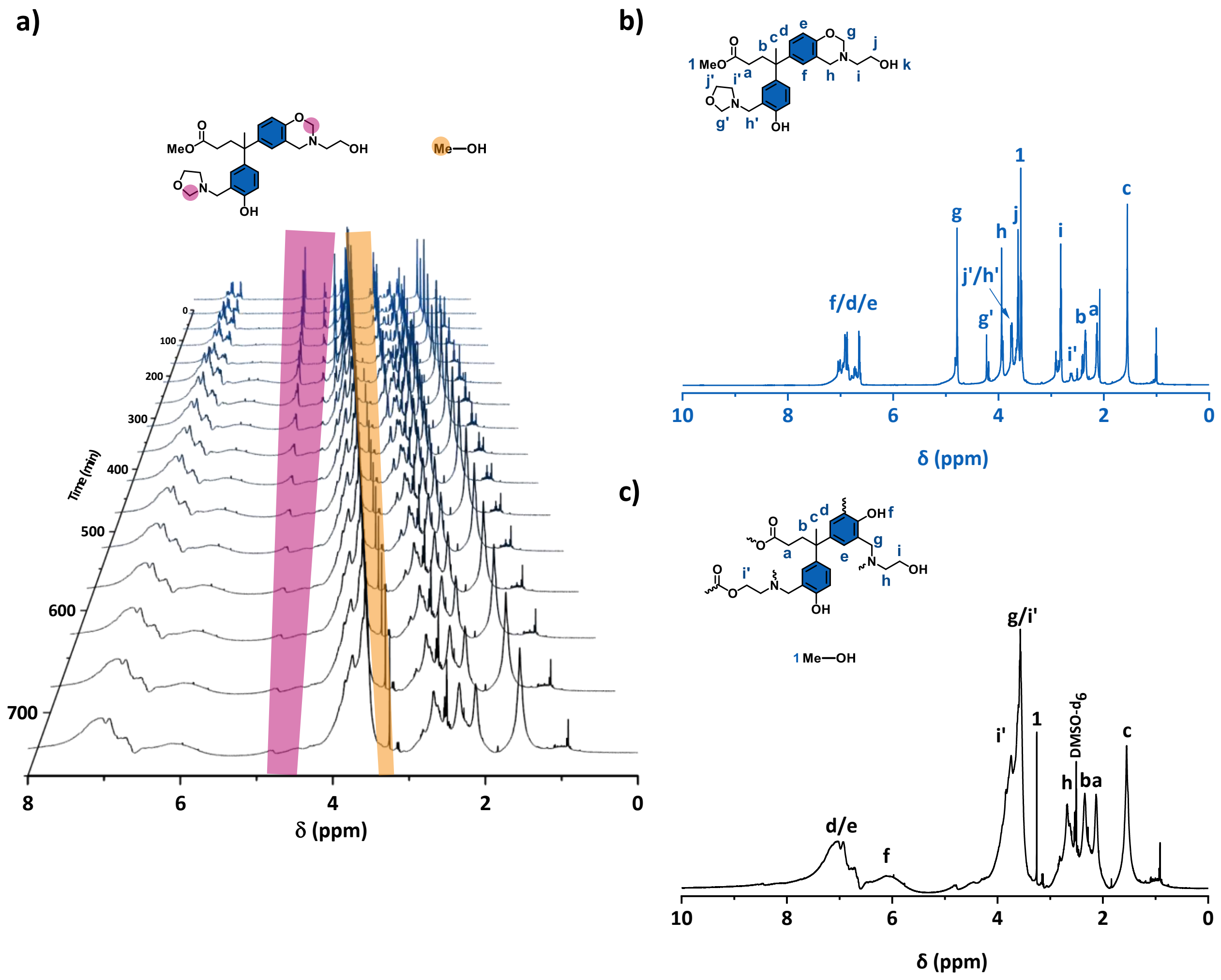

Figure S44 *a)* [1]*H kinetic NMR of Me-DPA-mea at 140 °C (DMSO-d*$_6$*): b) initial and c) final spectrum.*

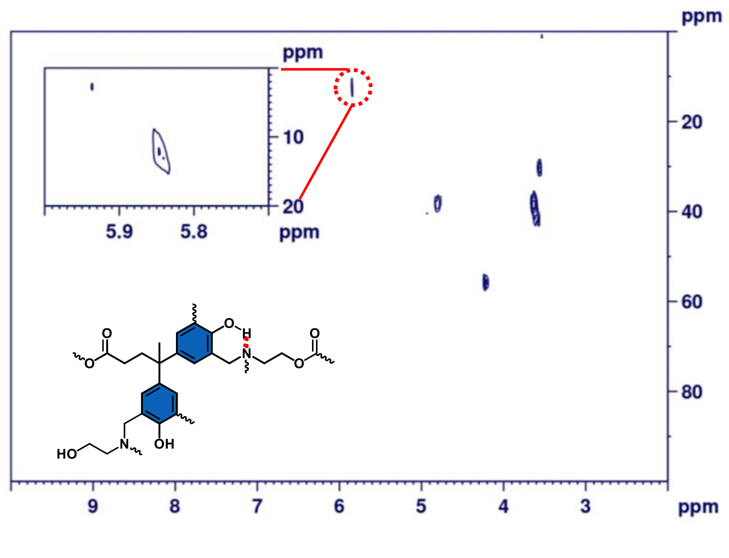

Figure S45 *Particular cross peak involving tertiary amine and phenol labile protons in* ^{15}*N-*^{1}*H HMBC kinetic of Me-DPA-mea.*

4. Mechanism of self-catalyzed transesterification in benzoxazine vitrimers

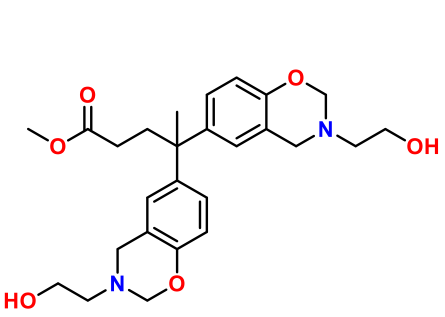

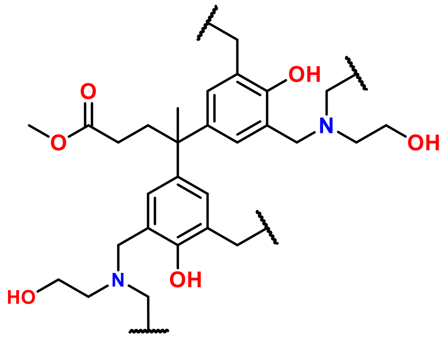

Table S4 *Extended Hückel calculation on opened and closed benzoxazine.*

Atom	Atom type (MM2)	Charge (MM2)	Charge (Hückel)
O(1)	O Alcohol	0	-0.37334
C(2)	C Alkane	0	0.143362
C(3)	C Alkane	0	0.019043
O(4)	O Alcohol	0	-0.37348
C(5)	C Alkane	0	0.143855
C(6)	C Alkane	0	0.018936
C(7)	C Alkane	0	0.234414
**N(8)**	**N Amine**	**0**	**-0.13283**
C(9)	C Alkane	0	0.023596
C(10)	C Alkane	0	0.023919
**N(11)**	**N Amine**	**0**	**-0.13239**
C(12)	C Alkane	0	0.233884
O(13)	O Enol	0	-0.27152
O(14)	O Enol	0	-0.27208
C(15)	C Alkene	0	-0.07958
C(16)	C Alkene	0	-0.02328
C(17)	C Alkene	0	0.207749
C(18)	C Alkene	0	-0.10174

Atom	Atom type (MM2)	Charge (MM2)	Charge (Hückel)
O(1)	O Alcohol	0	-0.37499
C(2)	C Alkane	0	0.146223
C(3)	C Alkane	0	0.018762
**N(4)**	**N Amine**	**0**	**-0.10977**
C(5)	C Alkane	0	0.014432
O(6)	O Enol	0	-0.28936
O(7)	O Enol	0	-0.28961
C(8)	C Alkene	0	-0.0995
C(9)	C Alkene	0	-0.00619
C(10)	C Alkene	0	0.18953
C(11)	C Alkene	0	-0.00572
C(12)	C Alkene	0	-0.10675
C(13)	C Alkene	0	-0.10082
C(14)	C Alkene	0	-0.00547
C(15)	C Alkene	0	0.186939
C(16)	C Alkene	0	-0.00537
C(17)	C Alkene	0	-0.10876
C(18)	C Alkane	0	-0.11617

C(19)	C Alkene	0	-0.07721
C(20)	C Alkene	0	-0.07928
C(21)	C Alkene	0	-0.10253
C(22)	C Alkene	0	0.206253
C(23)	C Alkene	0	-0.02238
C(24)	C Alkene	0	-0.081
C(25)	C Alkane	0	-0.11452
C(26)	C Alkene	0	0.02598
C(27)	C Alkene	0	0.023046
C(28)	C Alkane	0	0.041288
C(29)	C Alkane	0	-0.0453
C(30)	C Alkane	0	-0.12069
C(31)	C Carbonyl	0	0.588035
O(32)	O Carboxyl	0	-0.125
O(33)	O Carbonyl	0	-0.6245
C(34)	C Alkane	0	0.084011
H(35)	H Alcohol	0	0.197514
H(36)	H	0	0.015562
H(37)	H	0	0.012062
H(38)	H	0	0.024166
H(39)	H	0	0.004861
H(40)	H Alcohol	0	0.197608
H(41)	H	0	0.015617
H(42)	H	0	0.012036
H(43)	H	0	0.023799
H(44)	H	0	0.005098
H(45)	H	0	0.006326

C(19)	C Alkene	0	0.02411
C(20)	C Alkene	0	0.020379
C(21)	C Alkane	0	0.043291
C(22)	C Alkane	0	-0.01797
C(23)	C Alkane	0	-0.12276
C(24)	C Carbonyl	0	0.59013
O(25)	O Carboxyl	0	-0.12366
O(26)	O Carbonyl	0	-0.63105
C(27)	C Alkane	0	0.084056
C(28)	C Alkane	0	0.023928
C(29)	C Alkane	0	0.028408
C(30)	C Alkane	0	-0.05714
C(31)	C Alkane	0	-0.05699
**N(32)**	**N Amine**	**0**	**-0.12238**
C(33)	C Alkane	0	0.008558
C(34)	C Alkane	0	0.138559
O(35)	O Alcohol	0	-0.37397
C(36)	C Alkane	0	0.043209
C(37)	C Alkane	0	-0.141
C(38)	C Alkane	0	-0.11254
C(39)	C Alkane	0	-0.16844
C(40)	C Alkane	0	-0.11302
H(41)	H Alcohol	0	0.198442
H(42)	H	0	0.013391
H(43)	H	0	0.012457
H(44)	H	0	0.02316
H(45)	H	0	0.004373

H(46)	H	0	0.004048
H(47)	H	0	0.026655
H(48)	H	0	0.032485
H(49)	H	0	0.025955
H(50)	H	0	0.032589
H(51)	H	0	0.006434
H(52)	H	0	0.004142
H(53)	H	0	0.019544
H(54)	H	0	0.02415
H(55)	H	0	0.021866
H(56)	H	0	0.022668
H(57)	H	0	0.024076
H(58)	H	0	0.018386
H(59)	H	0	0.035593
H(60)	H	0	0.036456
H(61)	H	0	0.03758
H(62)	H	0	0.028692
H(63)	H	0	0.028791
H(64)	H	0	0.051554
H(65)	H	0	0.058801
H(66)	H	0	0.028315
H(67)	H	0	0.025903

H(46)	H	0	0.026722
H(47)	H	0	0.002249
H(48)	H Enol	0	0.208918
H(49)	H Enol	0	0.20969
H(50)	H	0	0.021437
H(51)	H	0	0.019937
H(52)	H	0	0.018443
H(53)	H	0	0.021026
H(54)	H	0	0.035899
H(55)	H	0	0.036561
H(56)	H	0	0.03756
H(57)	H	0	0.02843
H(58)	H	0	0.028218
H(59)	H	0	0.042589
H(60)	H	0	0.04399
H(61)	H	0	0.028302
H(62)	H	0	0.025933
H(63)	H	0	0.025844
H(64)	H	0	0.030019
H(65)	H	0	-0.00183
H(66)	H	0	0.023929
H(67)	H	0	0.026468
H(68)	H	0	0.036691
H(69)	H	0	0.030332
H(70)	H	0	0.03096
H(71)	H	0	0.036397
H(72)	H	0	0.031884

H(73)	H	0	0.011952
H(74)	H	0	0.015121
H(75)	H	0	0.011687
H(76)	H Alcohol	0	0.198257
H(77)	H	0	0.021518
H(78)	H	0	0.001212
H(79)	H	0	0.040017
H(80)	H	0	0.040243
H(81)	H	0	0.03991
H(82)	H	0	0.038836
H(83)	H	0	0.039345
H(84)	H	0	0.038832
H(85)	H	0	0.042059
H(86)	H	0	0.042558
H(87)	H	0	0.04131
H(88)	H	0	0.038879
H(89)	H	0	0.038845
H(90)	H	0	0.039893

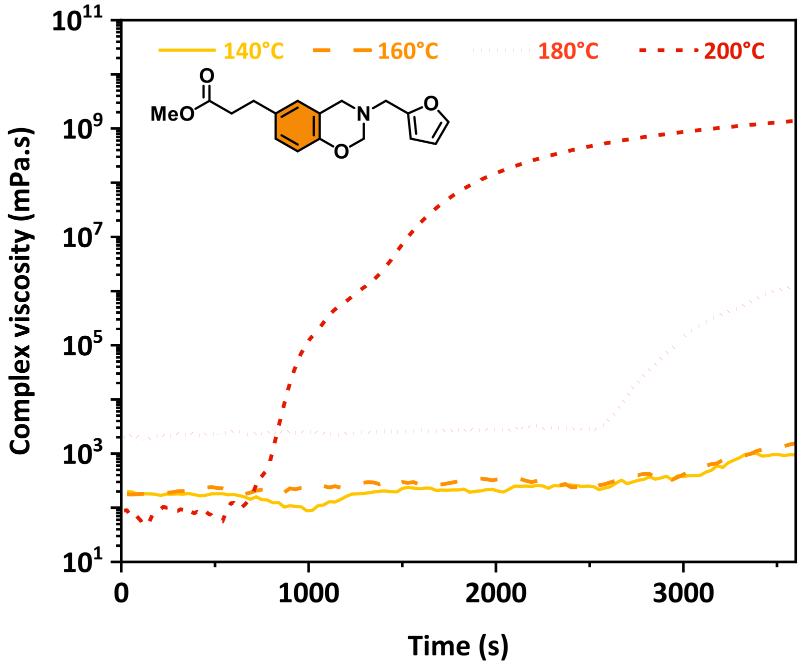

Figure S46 *Isothermal evolution of the complex viscosity as function of time of Me-PA-fa.*

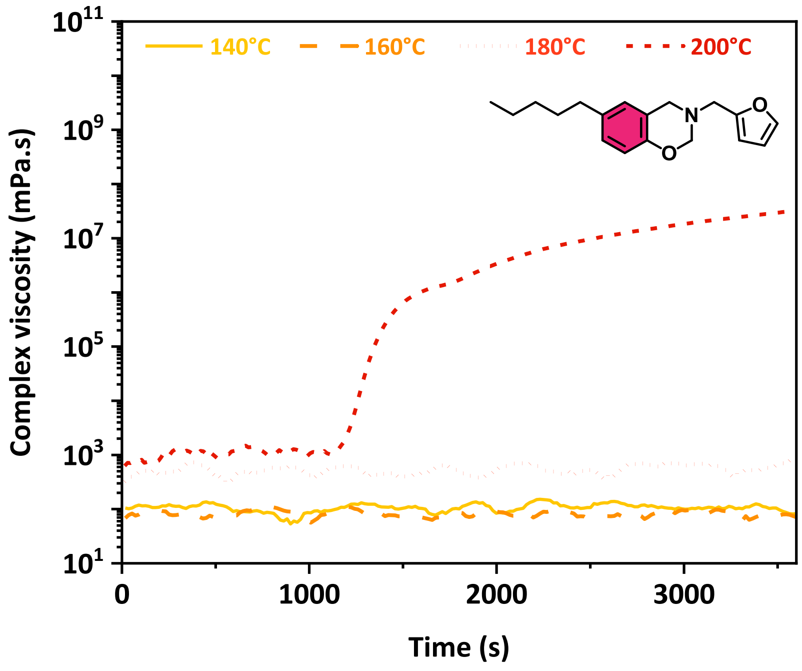

Figure S47 *Isothermal evolution of the complex viscosity as function of time of pPP-fa.*

References

1. H. E. Kissinger, Reaction Kinetics in Differential Thermal Analysis, *Analytical Chemistry*, 1957, **29**, 1702-1706.
2. J. H. Flynn and L. A. Wall, General Treatment of the Thermogravimetry of Polymers, *Journal of research of the National Bureau of Standards. Section A, Physics and chemistry*, 1966, **70A 6**, 487-523.
3. T. Ozawa, Kinetic analysis of derivative curves in thermal analysis, *Journal of thermal analysis*, 1970, **2**, 301-324.
4. H. L. Friedman, Kinetics of thermal degradation of char-forming plastics from thermogravimetry. Application to a phenolic plastic, *Journal of Polymer Science Part C: Polymer Symposia*, 1964, **6**, 183-195.
